# Supplementary material for: A national survey of antibacterial consumption in Sri Lanka
Source: PLoS One. 2021 Sep 14;16(9):e0257424. doi: 10.1371/journal.pone.0257424 (PMC8439449; doi:10.1371/journal.pone.0257424)
Supplement: S1 Table — (DOCX) [file pone.0257424.s001.docx]

| ATC code  **S1 Table: Comparing the volume of antibacterial consumption (ATC classification Level 5) between private and public sector**  Level 05 | ABM | Public sector  (%) | | Private sector  (%) | |
| --- | --- | --- | --- | --- | --- |
| J01A | Tetracycline’s | 5.94 | (6.06) | 14.89 | (6.03) |
| J01AA02 | Doxycycline | 5.94 | (6.06) | 14.36 | (5.82) |
| J01AA07 | Tetracycline | - | - | 0.53 | (0.22) |
| J01B | Amphenicols | 0 | (0.00) | 0.19 | (0.08) |
| J01BA01 | Chloramphenicol | 0 | (0.00) | 0.19 | (0.08) |
| J01C | Beta-lactam antibacterials ,Penicillins | 57.38 | (58.57) | 67.80 | (27.48) |
| J01CA01 | Ampicillin | 0.01 | (0.01) | 0.24 | (0.10) |
| J01CA04 | Amoxicillin | 21.91 | (22.37) | 22.66 | (9.18) |
| J01CE01 | Benzyl penicillin | 16.95 | (17.30) | - | - |
| J01CA08 | Pivmecillinam | - | - | 0.97 | (0.39) |
| J01CE02 | Phenoxymethyl penicillin | 1.44 | (1.47) | 0.44 | (0.18) |
| J01CE08 | Benzathine penicillin | 0 | (0.00) | - | - |
| J01CF02 | Cloxacillin | 7.96 | (8.13) | 2.49 | (1.01) |
| J01CF05 | Flucloxacillin | 0.32 | (0.33) | 1.93 | (0.78) |
| J01CR02 | Co-Amoxiclav | 8.72 | (8.90) | 39.07 | (15.83) |
| J01CR03 | Ticarcillin disodium & Clavulanate | 0.02 | (0.02) | - | - |
| J01CR05 | Piperacillin & Tazobactam | 0.06 | (0.06) | 0 | (0.00) |
| J01D | Other Beta-lactam antibacterials | 13.38 | 13.66 | 42.20 | (17.10) |
| J01DB01 | Cephalexin | 4.57 | (4.67) | 16.12 | (6.53) |
| J01DB09 | Cefradine |  |  | 0.04 | (0.02) |
| J01DC02 | Cefuroxime | 7.79 | (7.95) | 20.91 | (8.47) |
| J01DC04 | Cefaclor |  |  | 0.1 | (0.04) |
| J01DD01 | Cefotaxime | 0.14 | (0.14) | 0.03 | (0.01) |
| J01DD02 | Ceftazidime | 0.01 | (0.01) | 0.02 | (0.01) |
| J01DD04 | Ceftriaxone | 0.48 | (0.49) | 0.23 | (0.09) |
| J01DD08 | Cefixime | 0.05 | (0.05) | 4.59 | (1.86) |
| J01DD13 | Cefpodoxime Proxetil |  |  | 0.01 | (0.01) |
| J01DD62 | Cefoperazone & sulbactum | 0.02 | (0.02) | 0 | (0.00) |
| J01DE01 | Cefepime | 0 | (0.00) | 0 | (0.00) |
| J01DE02 | Cefpirome |  |  | 0 |  |
| J01DH02 | Meropenem | 0.3 | (0.31) | 0.12 | (0.05) |
| J01DH03 | Ertapenem | 0 | (0.00) | 0.01 | (0.00) |
| J01DH51 | Imipenem & Cilastatin | 0.01 | (0.01) | 0 | (0.00) |
| J01E | Sulfonamides and Trimethoprim | 0.5 | (0.51) | 1.71 | (0.69) |
| J01EC02 | Sulphadiazine | 0 | (0.00) | 0.01 | (0.00) |
| J01EE01 | Co-trimoxazole | 0.5 | (0.51) | 1.7 | (0.69) |
| J01F | Macrolide, Lincosamide and Streptogramins | 7.69 | (7.85) | 60.57 | (24.54) |
| J01FA01 | Erythromycin | 3.52 | (3.60) | 6.13 | (2.49) |
| J01FA06 | Roxithromycin | 0 | (0.00) | 0.91 | (0.37) |
| J01FA09 | Clarithromycin | 2.67 | (2.73) | 13.58 | (5.50) |
| J01FA10 | Azithromycin | 1.26 | (1.28) | 38.74 | (15.70) |
| J01FF01 | Clindamycin | 0.23 | (0.23) | 1.21 | (0.49) |
| J01G | Aminoglycoside antibacterials | 0.14 | (0.14) | 0 | (0.00) |
| J01GA01 | Streptomycin | 0 | (0.00) |  |  |
| J01GB03 | Gentamicin | 0.11 | (0.11) |  |  |
| J01GB06 | Amikacin Sulphate | 0.03 | (0.03) | 0 | (0.00) |
| J01GB07 | Netilmicin Sulfate | 0 | (0.00) | 0 | (0.00) |
| J01M | Quinolone Antibacterials | 6.66 | (6.80) | 46.57 | (18.87) |
| J01MA01 | Ofloxacin | 0.03 | (0.04) | 0.32 | (0.13) |
| J01MA02 | Ciprofloxacin | 5.81 | (5.93) | 34.93 | (14.16) |
| J01MA06 | Norfloxacin | 0.52 | (0.53) | 2.76 | (1.12) |
| J01MA12 | Levofloxacin | 0.22 | (0.22) | 8.05 | (3.26) |
| J01MA14 | Moxifloxacin |  |  | 0.35 | (0.14) |
| J01MB02 | Nalidixic acid | 0.08 | (0.08) | 0.16 | (0.06) |
| J01X | Other antibacterials | 6.28 | (6.41) | 12.84 | (5.20) |
| J01XA01 | Vancomycin | 0.04 | (0.04) | 0 | (0.00) |
| J01XA02 | Teicoplanin | 0.07 | (0.07) | 0.01 | (0.00) |
| J01XC01 | Sodium fusidate | 0.01 | (0.01) | 0.03 | (0.01) |
| J01XD01 | Metronidazole | 4.63 | (4.73) | 12.17 | (4.93) |
| J01XE01 | Nitrofurantoin | 1.52 | (1.55) | - | - |
| J01XX08 | Linezolid | 0.00 | (0.00) | 0.63 | (0.25) |
|  | Total | 97.96 | 100.00 | 246.76 | 100.00 |
