# Supplementary material for: A national survey of antibacterial consumption in Sri Lanka
Source: PLoS One. 2021 Sep 14;16(9):e0257424. doi: 10.1371/journal.pone.0257424 (PMC8439449; doi:10.1371/journal.pone.0257424)
Supplement: S2 Table — (DOCX) [file pone.0257424.s002.docx]

**S2 Table: AWaRe categorization of all the antibacterials**

| ATC Code | ABM | Public Sector  DDDs in Million | Public Sector  Percentage DDDs | Private Sector  DDDs in Million | Private Sector  Percentage DDDs | Total DDDs in Million | Total Percentage DDDs |
| --- | --- | --- | --- | --- | --- | --- | --- |
|  | **Access** | **74.34** | **75.89** | **112.42** | **45.56** | **186.76** | **54.18** |
| J01AA02 | Doxycycline | 5.94 | 6.06 | 14.36 | 5.82 | 20.30 | 5.89 |
| J01AA07 | Tetracycline |  | 0.00 | 0.53 | 0.21 | 0.53 | 0.15 |
| J01BA01 | Chloramphenicol | 0.00 | 0.00 | 0.19 | 0.08 | 0.19 | 0.06 |
| J01CA01 | Ampicillin | 0.01 | 0.01 | 0.24 | 0.10 | 0.25 | 0.07 |
| J01CA04 | Amoxicillin | 21.91 | 22.37 | 22.66 | 9.18 | 44.57 | 12.93 |
| J01CE01 | Benzyl penicillin | 16.95 | 17.30 |  | 0.00 | 16.95 | 4.92 |
| J01CA08 | Pivmecillinam |  | 0.00 | 0.97 | 0.39 | 0.97 | 0.28 |
| J01CE02 | Phenoxymethyl penicillin | 1.44 | 1.47 | 0.44 | 0.18 | 1.88 | 0.55 |
| J01CE08 | Benzathine penicillin | 0.00 | 0.00 |  | 0.00 | 0.00 | 0.00 |
| J01CF02 | Cloxacillin | 7.96 | 8.13 | 2.49 | 1.01 | 10.45 | 3.03 |
| J01CF05 | Flucloxacillin | 0.32 | 0.33 | 1.93 | 0.78 | 2.25 | 0.65 |
| J01CR02 | Co-Amoxiclav | 8.72 | 8.90 | 39.07 | 15.83 | 47.79 | 13.86 |
| J01DB01 | Cephalexin | 4.57 | 4.67 | 16.12 | 6.53 | 20.69 | 6.00 |
| J01DB09 | Cefradine |  | 0.00 | 0.04 | 0.02 | 0.04 | 0.01 |
| J01FF01 | Clindamycin | 0.23 | 0.23 | 1.21 | 0.49 | 1.44 | 0.42 |
| J01GB03 | Gentamicin | 0.11 | 0.11 |  | 0.00 | 0.11 | 0.03 |
| J01GB06 | Amikacin Sulphate | 0.03 | 0.03 | 0.00 | 0.00 | 0.03 | 0.01 |
| J01XD01 | Metronidazole | 4.63 | 4.73 | 12.17 | 4.93 | 16.80 | 4.87 |
| J01XE01 | Nitrofurantoin | 1.52 | 1.55 |  | 0.00 | 1.52 | 0.44 |
|  | **Watch** | **23.51** | **24.00** | **133.53** | **54.11** | **157.04** | **45.56** |
| J01CR05 | Piperacillin & Tazobactam | 0.06 | 0.06 | 0.00 | 0.00 | 0.06 | 0.02 |
| J01DC02 | Cefuroxime | 7.79 | 7.95 | 20.91 | 8.47 | 28.70 | 8.33 |
| J01DC04 | Cefaclor |  | 0.00 | 0.10 | 0.04 | 0.10 | 0.03 |
| J01DD01 | Cefotaxime | 0.14 | 0.14 | 0.03 | 0.01 | 0.17 | 0.05 |
| J01DD02 | Ceftazidime | 0.01 | 0.01 | 0.02 | 0.01 | 0.03 | 0.01 |
| J01DD04 | Ceftriaxone | 0.48 | 0.49 | 0.23 | 0.09 | 0.71 | 0.21 |
| J01DD08 | Cefixime | 0.05 | 0.05 | 4.59 | 1.86 | 4.64 | 1.35 |
| J01DD13 | Cefpodoxime Proxetil |  | 0.00 | 0.01 | 0.00 | 0.01 | 0.00 |
| J01DD62 | Cefoperazone & sulbactum | 0.02 | 0.02 | 0.00 | 0.00 | 0.02 | 0.01 |
| J01DE01 | Cefepime | 0.00 | 0.00 | 0.00 | 0.00 | 0.00 | 0.00 |
| J01DE02 | Cefpirome |  | 0.00 | 0.00 | 0.00 | 0.00 | 0.00 |
| J01DH02 | Meropenem | 0.30 | 0.31 | 0.12 | 0.05 | 0.42 | 0.12 |
| J01DH03 | Ertapenem | 0.00 | 0.00 | 0.01 | 0.00 | 0.01 | 0.00 |
| J01DH51 | Imipenem & Cilastatin | 0.01 | 0.01 | 0.00 | 0.00 | 0.01 | 0.00 |
| J01EE01 | Co-trimoxazole | 0.50 | 0.51 | 1.70 | 0.69 | 2.20 | 0.64 |
| J01FA01 | Erythromycin Stearate | 3.52 | 3.59 | 6.13 | 2.48 | 9.65 | 2.80 |
| J01FA06 | Roxithromycin | 0.00 | 0.00 | 0.91 | 0.37 | 0.91 | 0.26 |
| J01FA09 | Clarithromycin | 2.67 | 2.73 | 13.58 | 5.50 | 16.25 | 4.71 |
| J01FA10 | Azithromycin | 1.26 | 1.29 | 38.74 | 15.70 | 40.00 | 11.60 |
| J01GA01 | Streptomycin | 0.00 | 0.00 |  | 0.00 | 0.00 | 0.00 |
| J01GB07 | Netilmicin Sulfate | 0.00 | 0.00 | 0.00 | 0.00 | 0.00 | 0.00 |
| J01MA01 | Ofloxacin | 0.03 | 0.03 | 0.32 | 0.13 | 0.35 | 0.10 |
| J01MA02 | Ciprofloxacin | 5.81 | 5.93 | 34.93 | 14.16 | 40.74 | 11.82 |
| J01MA06 | Norfloxacin | 0.52 | 0.53 | 2.76 | 1.12 | 3.28 | 0.95 |
| J01MA12 | Levofloxacin | 0.22 | 0.22 | 8.05 | 3.26 | 8.27 | 2.40 |
| J01MA14 | Moxifloxacin |  | 0.00 | 0.35 | 0.14 | 0.35 | 0.10 |
| J01XA01 | Vancomycin | 0.04 | 0.04 | 0.00 | 0.00 | 0.04 | 0.01 |
| J01XA02 | Teicoplanin | 0.07 | 0.07 | 0.01 | 0.00 | 0.08 | 0.02 |
| J01XC01 | Sodium fusidate | 0.01 | 0.01 | 0.03 | 0.01 | 0.04 | 0.01 |
|  | **Reserve group** | **0.00** | **0.00** | **0.63** | **0.26** | **0.63** | **0.18** |
| J01XX08 | Linezolid | 0.00 | 0.00 | 0.63 | 0.26 | 0.63 | 0.18 |
|  | Not listed in AwaRe |  |  |  |  |  |  |
| J01CR03 | Ticarcillin disodium & Clavulanate | 0.02 | 0.02 |  | 0.00 | 0.02 | 0.01 |
| J01EC02 | Sulphadiazine | 0.00 | 0.00 | 0.01 | 0.00 | 0.01 | 0.00 |
| J01MB02 | Nalidixic acid | 0.08 | 0.08 | 0.16 | 0.06 | 0.24 | 0.07 |
| Total |  | 97.96 | 100.00 | 246.76 | 100.00 | 344.72 | 100.00 |
