# Supplementary material for: A national survey of antibacterial consumption in Sri Lanka
Source: PLoS One. 2021 Sep 14;16(9):e0257424. doi: 10.1371/journal.pone.0257424 (PMC8439449; doi:10.1371/journal.pone.0257424)
Supplement: Attachment — Submitted filename: PONE-D-21-11873_reviewer.pdf [file pone.0257424.s003.pdf]

PLOS ONE

National surveillance of antibacterial consumption in Sri Lanka

--Manuscript Draft--

**Manuscript Number:**	PONE-D-21-11873
**Article Type:**	Research Article
**Full Title:**	National surveillance of antibacterial consumption in Sri Lanka
**Short Title:**	National surveillence of antibacterial consumption
**Corresponding Author:**	Shalini Sri Ranganathan<br>University of Colombo Faculty of Medicine<br>Colombo, SRI LANKA
**Keywords:**	antibacterials; consumption; surveillence; antibacterial resistance; utilization
**Abstract:**	Objective<br><br>To quantify and describe the national antibacterial consumption in Sri Lanka<br><br>Methods<br><br>The methodology was adapted from WHO methodology for a global programme on surveillance of antimicrobial consumption. Aggregate data on national consumption of systemic antibacterials (J01- Anatomical Therapeutic Chemical Classification (ATC)) in 2017 were retrospectively extracted from various data sources and classified using ATC classification. Quantity of consumption was converted to Defined Daily Doses (DDDs). Data are presented as total consumption and comparison between the public and private sector. We also compared few key indicators of antibacterial consumption between these two sectors.<br><br>Findings<br><br>From the available data sources, the total ABC in 2017 was 343.46 million DDDs. Private sector consumption accounted for 246.76 million DDDs compared to 97.96 million DDDs distributed to entire public sector by the Ministry of Health. Beta-lactam antibacterials, penicillins accounted for 58.79% of public sector compared to 27.48% in the private sector while macrolides, quinolones and other beta-lactam antibacterials accounted for 60.51% in the private compared to 28.41% in public sector. Consumption of reserve group antibacterials was negligible, and limited to private sector. Reserve and watch category antibacterials accounted for 46, 24 and 54% of the total, public and private sector consumption.<br><br>Conclusions<br><br>The single most important difference observed between public and private sector was disproportionately higher use of broad spectrum and “Watch category” ABAs in the private sector. Our study has provided the evidence that antibacterial surveillance is possible in resource limited countries and it must be made mandatory.
**Order of Authors:**	Shalini Sri Ranganathan
	Chandanie Wanigatunge
	G.P.S. G Senadheera
	BVSH Beneragama
**Additional Information:**	
**Question**	**Response**
**Financial Disclosure**<br><br>Enter a financial disclosure statement that	The author(s) received no specific funding for this work.

Powered by Editorial Manager® and ProduXion Manager® from Aries Systems Corporation

describes the sources of funding for the work included in this submission. Review the submission guidelines for detailed requirements. View published research articles from *PLOS ONE* for specific examples.<br><br>This statement is required for submission and **will appear in the published article** if the submission is accepted. Please make sure it is accurate.<br><br>**Unfunded studies**<br>Enter: *The author(s) received no specific funding for this work.*<br><br>**Funded studies**<br>Enter a statement with the following details:<br>• Initials of the authors who received each award<br>• Grant numbers awarded to each author<br>• The full name of each funder<br>• URL of each funder website<br>• Did the sponsors or funders play any role in the study design, data collection and analysis, decision to publish, or preparation of the manuscript?<br>• **NO** - Include this sentence at the end of your statement: *The funders had no role in study design, data collection and analysis, decision to publish, or preparation of the manuscript.*<br>• **YES** - Specify the role(s) played.<br><br>* typeset	
**Competing Interests**<br><br>Use the instructions below to enter a competing interest statement for this submission. On behalf of all authors, disclose any competing interests that could be perceived to bias this work—acknowledging all financial support and any other relevant financial or non-financial competing interests.<br><br>This statement is required for submission and **will appear in the published article** if the submission is accepted. Please make sure it is accurate and that any funding	The authors have declared that no competing interests exist.

Powered by Editorial Manager® and ProduXion Manager® from Aries Systems Corporation

sources listed in your Funding Information later in the submission form are also declared in your Financial Disclosure statement.<br><br>View published research articles from *PLOS ONE* for specific examples.<br><br>**NO authors have competing interests**<br><br>Enter: *The authors have declared that no competing interests exist.*<br><br>**Authors with competing interests**<br><br>Enter competing interest details beginning with this statement:<br><br>*I have read the journal's policy and the authors of this manuscript have the following competing interests: [insert competing interests here]*<br><br>* typeset	
**Ethics Statement**<br><br>Enter an ethics statement for this submission. This statement is required if the study involved:<br><br>• Human participants<br>• Human specimens or tissue<br>• Vertebrate animals or cephalopods<br>• Vertebrate embryos or tissues<br>• Field research<br><br>Write "N/A" if the submission does not require an ethics statement.<br><br>General guidance is provided below. Consult the submission guidelines for detailed instructions. **Make sure that all information entered here is included in the Methods section of the manuscript.**	N/A

**Format for specific study types**<br><br>**Human Subject Research (involving human participants and/or tissue)**<br>• Give the name of the institutional review board or ethics committee that approved the study<br>• Include the approval number and/or a statement indicating approval of this research<br>• Indicate the form of consent obtained (written/oral) or the reason that consent was not obtained (e.g. the data were analyzed anonymously)<br><br>**Animal Research (involving vertebrate animals, embryos or tissues)**<br>• Provide the name of the Institutional Animal Care and Use Committee (IACUC) or other relevant ethics board that reviewed the study protocol, and indicate whether they approved this research or granted a formal waiver of ethical approval<br>• Include an approval number if one was obtained<br>• If the study involved *non-human primates*, add *additional details* about animal welfare and steps taken to ameliorate suffering<br>• If anesthesia, euthanasia, or any kind of animal sacrifice is part of the study, include briefly which substances and/or methods were applied<br><br>**Field Research**<br><br>Include the following details if this study involves the collection of plant, animal, or other materials from a natural setting:<br>• Field permit number<br>• Name of the institution or relevant body that granted permission	
**Data Availability**<br><br>Authors are required to make all data underlying the findings described fully available, without restriction, and from the time of publication. PLOS allows rare exceptions to address legal and ethical concerns. See the PLOS Data Policy and FAQ for detailed information.	No - some restrictions will apply

Powered by Editorial Manager® and ProduXion Manager® from Aries Systems Corporation

A Data Availability Statement describing where the data can be found is required at submission. Your answers to this question constitute the Data Availability Statement and **will be published in the article**, if accepted.<br><br>**Important:** Stating 'data available on request from the author' is not sufficient. If your data are only available upon request, select 'No' for the first question and explain your exceptional situation in the text box.<br><br>Do the authors confirm that all data underlying the findings described in their manuscript are fully available without restriction?	
**Describe where the data may be found in full sentences. If you are copying our sample text, replace any instances of XXX with the appropriate details.**<br><br>• If the data are **held or will be held in a public repository**, include URLs, accession numbers or DOIs. If this information will only be available after acceptance, indicate this by ticking the box below. For example: *All XXX files are available from the XXX database (accession number(s) XXX, XXX.*).<br>• If the data are all contained **within the manuscript and/or Supporting Information files**, enter the following: *All relevant data are within the manuscript and its Supporting Information files.*<br>• If neither of these applies but you are able to provide **details of access elsewhere**, with or without limitations, please do so. For example:<br><br>*Data cannot be shared publicly because of [XXX]. Data are available from the XXX Institutional Data Access / Ethics Committee (contact via XXX) for researchers who meet the criteria for access to confidential data.*<br><br>*The data underlying the results presented in the study are available from (include the name of the third party*	Data cannot be shared publicly because of they belong to the Ministry of Health and some private and state instutions. Raw Data are available from the investigators for researchers who meet the criteria for access to confidential data.

and contact information or URL).<br>• This text is appropriate if the data are owned by a third party and authors do not have permission to share the data.<br><br>* typeset	
Additional data availability information:	

Powered by Editorial Manager® and ProduXion Manager® from Aries Systems Corporation

Manuscript
Click here to access/download;Manuscript;SLACPT_ABC_For PLoS_HSR.docx

National surveillance of antibacterial consumption in Sri Lanka

Title: **National surveillance of antibacterial consumption in Sri Lanka**

Authors: Shalini Sri Ranganathan[1,5], Chandanie Wanigatunge [2,5], G.P.S. G.Senadheera[3,] BVSH Beneragama[4]

Institutions

1. Department of Pharmacology, Faculty of Medicine, University of Colombo, Sri Lanka
2. Department of Pharmacology, Faculty of Medical Sciences, University of Sri Jayewardenepura, Sri Lanka
3. Department of Pharmacy and pharmaceutical Sciences, Faculty of Allied Health Sciences, University of Sri Jayewardenepura, Sri Lanka
4. Deputy Director General (Laboratory Services), National focal point for combating antimicrobial resistance in Sri Lanka, Ministry of Health
5. Sri Lanka Association for Clinical Pharmacology and Therapeutics (SLACPT)

Corresponding author

Name: Shalini Sri Ranganathan

Address: Department of Pharmacology, Faculty of Medicine, University of Colombo, Sri Lanka

Email sshalini@pharm.cmb.ac.lk or sshalini14@hotmail.com

Any disclaimers: None

Sources of support: Sri Lanka Association of Clinical Pharmacology and Therapeutics provided funds for data entry.

Word count: 2572 (excluding abstract, acknowledgements, references, tables and figures)

Number of tables= 4

Number of figures = 2

Abstract

Objective: To quantify and describe the national antibacterial consumption in Sri Lanka

Methods: The methodology was adapted from WHO methodology for a global programme on surveillance of antimicrobial consumption. Aggregate data on national consumption of systemic antibacterials (J01- Anatomical Therapeutic Chemical Classification (ATC)) in 2017 were retrospectively extracted from various data sources and classified using ATC classification. Quantity of consumption was converted to Defined Daily Doses (DDDs). Data are presented as total consumption and comparison between the public and private sector. We also compared few key indicators of antibacterial consumption between these two sectors.

Findings: From the available data sources, the total ABC in 2017 was 343.46 million DDDs. Private sector consumption accounted for 246.76 million DDDs compared to 97.96 million DDDs distributed to entire public sector by the Ministry of Health. Beta-lactam antibacterials, penicillins accounted for 58.79 % of public sector compared to 27.48 % in the private sector while macrolides, quinolones and other beta-lactam antibacterials accounted for 60.51 % in the private compared to 28.41% in public sector. Consumption of reserve group antibacterials was negligible, and limited to private sector. Reserve and watch category antibacterials accounted for 46, 24 and 54% of the total, public and private sector consumption.

Conclusions: The single most important difference observed between public and private sector was disproportionately higher use of broad spectrum and “Watch category” ABAs in the private sector. Our study has provided the evidence that antibacterial surveillance is possible in resource limited countries and it must be made mandatory.

Introduction:

Antimicrobial resistance (AMR) is a major global public health challenge. A global survey conducted by the World Health Organization (WHO) in 2014 has shown a high level of resistance to both first line and reserve antibacterials was seen for nine pathogenic bacteria responsible for common infections in all WHO regions (1). Infection with such resistant microorganisms result in longer illnesses, increased mortality, prolonged hospital stays and increased overall costs. AMR affects all areas of health including veterinary and environmental practices and impacts the entire society and its economy. Countering AMR needs long term strategies which include strengthening the health systems to ensure more appropriate use of and access to antimicrobial agents.

The development of AMR is an evolutionary process of microbes and is accelerated by the use of antimicrobials. There is a strong association between AMR and levels of antimicrobial use, and reduction of inappropriate use of antimicrobials could reduce development of resistance (2, 3). However, there appears to be an increase in global consumption of antibacterials, more in low- and middle-income countries (4, 5) with an increase in the use of broad-spectrum and last-resort antibacterials (5).

The WHO's Global Action Plan (GAP) for Antimicrobial Resistance (6) calls for member states to put in place national plans to urgently combat AMR. Five strategic objectives have been identified to achieve the goals of the GAP. The fourth objective viz to "Optimize the use of antimicrobial medicines in human and animal health" necessitates provision of reliable antimicrobial consumption (AMC) data. While data on antibiotic use are collected and analysed in many high- and middle-income countries, there is severe paucity of AMC data from lower-income countries (7). Data on AMC are vital to understand AMR, as selection pressure due to use

of antimicrobials is a main preventable driver for development and spread of AMR (2, 3). To effectively curtail AMR, surveillance data from AMR must be linked to that of AMC.

The WHO methodology for a global programme on surveillance of antimicrobial consumption provides a practical framework to obtain such data in resource limited countries (RLC) (8). This involves the collection of “Consumption” and “Use” data and recommends that countries separate “consumption data” from “use data” as the objectives, methods and outcomes for these two categories of data are different. Consumption data” refers to estimates derived from of aggregated data, mainly derived from import, sales or reimbursement databases whereas “use data” refers to estimates derived from patient-level (8).

Sri Lanka is a lower middle-income country (9). Both the public and the private sectors provide allopathic healthcare services in Sri Lanka but the share of care being different for inpatients and outpatients. The public sector provides the bulk of inpatient care (10) while outpatient care is shared. The country imports the bulk of its antimicrobials through the State Pharamceuticals Corporation (SPC), which is the State’s procurement arm, and independent private importers. Limited amounts of antimicrobials are manufactured by the State Pharmaceticals Manufacturing Corporation (SPMC) and individual local manufacturers. The State Pharamceuticals Corporation is the sole supplier of antimicrobials to the public sector and procures antibacterials from local manufaturers in addition to imports. The animicrobials are distributed to State medical institutions by the Medical Supplies Division (MSD) of the Ministry of Health and when antibacterials are not available at the MSD, the individual hospitals procure antibacterials from the private retail pharmacies as local purchases. Antimicrobials to private healthcare institutions are supplied by both the SPC and independent private importers and local manufacturers.

Sri Lanka has an established and successful AMR surveillance programme, coordinated by the Sri Lanka College of Microbiologists. However, there is no sytem to obtain aggregated AMC data at present. The Sri Lanka Association of Clinical Pharmacology and Therapeutics (SLACPT), in collaboration the National Focal Point for combating AMR in Sri Lanka, therefore conducted a national surveillance of antibacterial consumption (ABC). This paper outlines the methods adopted, key findings and recommendations for establishment of a national surveillance programme.

Methods

Background: The methodology of this study was adapted from WHO methodology for a global programme on surveillance of antimicrobial consumption (4).

Study design and setting: It was a descriptive cross-sectional study in which aggregate data on antibacterial consumption in 2017 were retrospectively extracted from various data sources in 2018.

Antibacterial agents: The WHO methodology recommends to survey antimicrobials including anti-protozoals, anti-fungals, anti-malarials and anti-virals in addition to antibacterial agents (ABAs). However, to start with, we have surveyed only the ABAs listed under antibacterials for systemic use (J01) in the Anatomical Therapeutic Chemical (ATC) classification system (11).

Definition of consumption data: The WHO defines consumption data as "estimates derived from aggregated data sources such as import or wholesaler data, or aggregated health insurance data where there is no information available on the patients who are receiving the medicines or why they are being used"(4). National antibacterial consumption (ABC) data provide a proxy estimate of use of these agents in the country.

ABAs suppliers: Local manufacturing of ABAs is limited in Sri Lanka leaving the importers as the major supplier of ABAs. State Pharmaceutical Corporation is the sole importer for public sector and Rajya Osusala Pharmacies (retail pharmacy chain of SPC). It also imports for private market. In addition, there are many importers who cater for private market

Data sources: Considering the supply system in Sri Lanka, we approached the Sri Lanka customs, Department of Imports and Exports, SPC, State Pharmaceutical Manufacturing Corporation (SPMC), Medical Supplies Division (MSD), private importers and the private manufacturers (list was obtained from the NMRA website, www.nmra.gov.lk accessed on 31st August 2018) for ABC

data. Formal request letter with evidence of administrative and ethical approval was sent to all these institutions. Investigators personally visited many of these data sources. We accepted both electronic and paper data from the institutions who had extractable ABC data and were willing to provide them.

Available data: The WHO methodology recommends a detailed product-level electronic data to be collected for ABC surveillance programmes. However, for this surveillance, data which had the minimum details, namely name, dosage form, strength and quantity were considered as "complete" and included for analysis.

Data entry: A custom-made MS Excel template was developed based on WHO methodology and our previous experience in ABC surveillance in Colombo district (7). Except the data from the MSD which were electronically transferred, the rest were manually entered as they were in paper format. All the precautions were taken to ensure the accuracy of data entry.

Data analysis: Antibacterials for systemic use (J01, ATC classification level 2) obtained from the data sources were further classified to level 3, 4 and 5. We have presented the consumption data at level 3 (therapeutic or pharmacological sub-group) and level 5 (chemical substance). Quantity of consumption is expressed as Defined Daily Doses (DDDs) using the formula given below. The DDD is defined as "the assumed average maintenance dose per day for a medicine used for its main indication in adults". Total grams consumed was determined by summing the amounts of active ingredient across the various formulations (different strengths of tablets or capsules, syrup formulations) and pack sizes. The DDD value is assigned by the WHO Collaborating Centre and obtained from their website (http:// www.whocc.no/atc_ddd_index/)

$$\textbf{Number of DDDs used} = \frac{\textbf{Total grams consumed (in grams)}}{\textbf{DDD (in grams)}}$$

Though the WHO methodology recommends that the variables for consumption estimates should include packages and DDDs, we have used only DDDs as package details were not available for this retrospective surveillance.

Presentation of data: In addition to presenting the total consumption data obtained from different data sources, we have also compared the public sector data with that of private sector, keeping in mind that the private sector data could be an underestimate as we have no way to verify the accuracy and completeness of data. We also have compared few key indicators of ABC as well as the volumes of ABAs in the "Access, Watch and Reserve" categories (AWaRe classification) between these two sectors (12-14)

Ethics and administration approval: Ethics Review Committee of Sri Lanka Medical Association exempted this survey from ethics review committee approval (ERC-18/14)

Results:

Background: Of the potential data sources we approached, only SPC, MSD, SPMC (out of 5 local manufacturers contacted) and 12 private importers (out of 78 contacted) provided analyzable data. Both Sri Lanka Customs and Department of Imports and Exports did not have extractable data. We had to discard data provided by a major importer as they lacked the information on strength of the products which is essential to calculate DDD.

Table 1 shows the total volume of ABAs (in million DDDs) imported/distributed in 2017 by the respective agencies. Consumption of ABAs was very much higher in the private than the public sector: Private sector consumption (Table 1; data sources 1, 4 and 7) accounted for 246.76 million DDDs compared to 97.96 million DDDs distributed to entire public sector by the MSD (data source 2). Additionally, of the annual volume of ABAs distributed by the SPC, the sole agent for the public sector, private sector accounted for 74.5% followed by MSD (14.35%) and Rajya Osusala Pharmacies (11.15%). Table 2 compares the consumption of different pharmacological sub-groups of ABAs (3rd level of ATC classification) between public (data source 2) and private sector (data sources 1, 4, 7).

Though the top four groups of ABAs were the same for public and private sector, namely (1) beta-lactam antibacterials, penicillins, (2) other beta-lactam antibacterials, (3) macrolides, and (4) quinolones, major difference in the proportion of volumes consumed was observed between these two sectors; beta-lactam antibacterials, penicillins considerably outnumbered the other groups in the public sector (8.62:2.01:1.16: 1.00) whereas the proportions were more or less equal in the private sector (1.61:1.00:1.44: 1.10) . Consumption of macrolides, quinolones and other beta lactam ABAs appears to be disproportionately higher in the private compared to public sector.

Table 3 compares the consumption of most frequently consumed ABAs (5th and last level of ATC classification) within these top four groups between the public and private sector (complete data are available in the supplementary Tables). Substantial differences in the consumption of individual ABAs was observed between public and private sector. For example, consumption of co-amoxiclav, cefuroxime, cefixime, clarithromycin, azithromycin, ciprofloxacin and levofloxacin appears to be disproportionately higher in the private compared to public sector. Table 4 compares the key indicators of ABC between public and private sector. Figure 1 compares the consumption of ABAs in the “Access, Watch and Reserve “categories between public and private sector. The single most important difference observed between public and private sector (Tables 2,3 and 4, Figure 1) was disproportionately higher use of broad spectrum ABAs in the private sector.

Figure 2 shows the top 10 ABAs, oral ABAs and parenteral ABAs consumed in public and private sector. It appears that benzylpenicillin and amoxicillin are scarcely consumed in private as opposed to public sector (Detail tables are available with supplementary data).

Table 1: Total volume of ABAs (in million DDDs) imported/distributed in 2017 by the different agencies

Agency	Volume of ABAs (in million DDDs)[8]
1. Distributed by SPC to private sector	163.04
2. Distributed by MSD to public sector [1]	97.96
3. Distributed by SPMC to both to public and private sector	61.18
4. Imported by private sector	59.30
5. Manufactured by SPMC	56.08
6. Distributed by SPC to MSD [1]	31.43
7. Distributed by SPC to its retail pharmacies (Rajya Osusala)	24.41

1. *MSD distributed to public sector is higher than the SPC distributed to MSD because left over stocks from 2016 could have been distributed by the MSD.*
2. *Because of the overlaps between Agencies, we did not total the volume*

Table 2: Consumption of different pharmacological sub-groups of ABAs in private vs. public sector

ATC level 3 classification of ABAs	ABA consumption volume in DDD per million			
	Public sector (%)		Private sector (%)	
Tetracycline's	5.94	(6.06)	14.89	(6.03)
Amphenicols	0.00	(0.00)	0.19	(0.08)
Beta-lactam antibacterials, Penicillins	57.38	(58.57)	67.80	(27.47)
Other Beta-lactam antibacterials	13.38	(13.66)	42.20	(17.10)
Sulfonamides and Trimethoprim	0.50	(0.51)	1.71	(0.69)
Macrolide, Lincosamide and Streptogramins	7.69	(7.78)	60.57	(24.54)
Aminoglycoside antibacterials	0.14	(0.14)	0.00	(0.00)
Quinolone Antibacterials	6.66	(6.82)	46.57	(18.87)
Other antibacterials	6.28	(6.41)	12.84	(5.20)
**Total**	**97.96**	**100.00**	**246.76**	**100.00**

Table 3: Consumption of individual ABAs in the top four pharmacological groups in private vs. public sector

ATC level 5 classification of ABAs (Code) under each level 3 classification	Name of classes and individual ABAs	Private sector (%)		Public sector (%)	
		DDDs	(%)	DDDs	(%)
J01C	Beta-lactam antibacterials, Penicillins				
J01CA04	Amoxicillin	22.66	(9.18)	21.91	(22.37)
J01CE01	Benzyl penicillin	-	-	16.95	(17.30)
J01CF02	Cloxacillin	2.49	(1.01)	7.96	(8.12)
J01CF05	Flucloxacillin	1.93	(0.78)	0.32	(0.33)
J01CR02	Co-Amoxiclav	39.07	(15.83)	8.72	(8.90)
J01D	Other Beta-lactam antibacterials				
J01DB01	Cephalexin	16.12	(6.53)	4.57	(4.67)
J01DC02	Cefuroxime	20.91	(8.47)	7.79	(7.95)
J01DD08	Cefixime	4.59	(1.86)	0.05	(0.05)
J01F	Macrolide, Lincosamide and Streptogramins				
J01FA01	Erythromycin Stearate	6.13	(2.49)	3.52	(3.60)
J01FA09	Clarithromycin	13.58	(5.50)	2.67	(2.73)
J01FA10	Azithromycin	38.74	(15.70)	1.26	(1.28)
J01M	Quinolone antibacterials				
J01MA01	Ofloxacin	0.32	(0.13)	0.03	(0.04)
J01MA02	Ciprofloxacin	34.93	(14.16)	5.81	(5.93)
J01MA06	Norfloxacin	2.76	(1.12)	0.52	(0.53)
J01MA12	Levofloxacin	8.05	(3.26)	0.22	(0.22)

Table 4: Comparison of few key indicators of ABC between public and private sector

	Indicator	Private sector	Public sector
J01CE_%	Consumption of β-lactamase sensitive penicillins (J01CE) expressed as percentage of the total consumption of antibacterials for systemic use (J01)	0.18	18.77
J01CR_%	Consumption of combination of penicillins, including β -lactamase inhibitor (J01CR) expressed as percentage of the total consumption of antibacterials for systemic use (J01)	15.83	8.98
J01DD+DE_%	Consumption of third and fourth generation of cephalosporins (J01(DD+DE)) expressed as percentage of the total consumption of antibacterials for systemic use (J01)		
	1st Generation	6.55	4.67
	2nd Generation	8.52	7.95
	3rd Generation	1.98	0.72
	4th Generation	0.00	0.00
J01MA_%	Consumption of flouroquinolones (J01MA) expressed as percentage of the total consumption of antibacterials for systemic use (J01)	18.81	6.71
J01_B/N	Ratio of the consumption of broad (J01(CR+DC+DD+(F-FA01))) to the consumption of narrow spectrum penicillins, cephalosporins and macrolides (J01(CE+DB+FA01))	5.25	0.81

Figure 1: Consumption of ABAs in Access, Watch and Reserve categories: Comparing total, public and private sector data

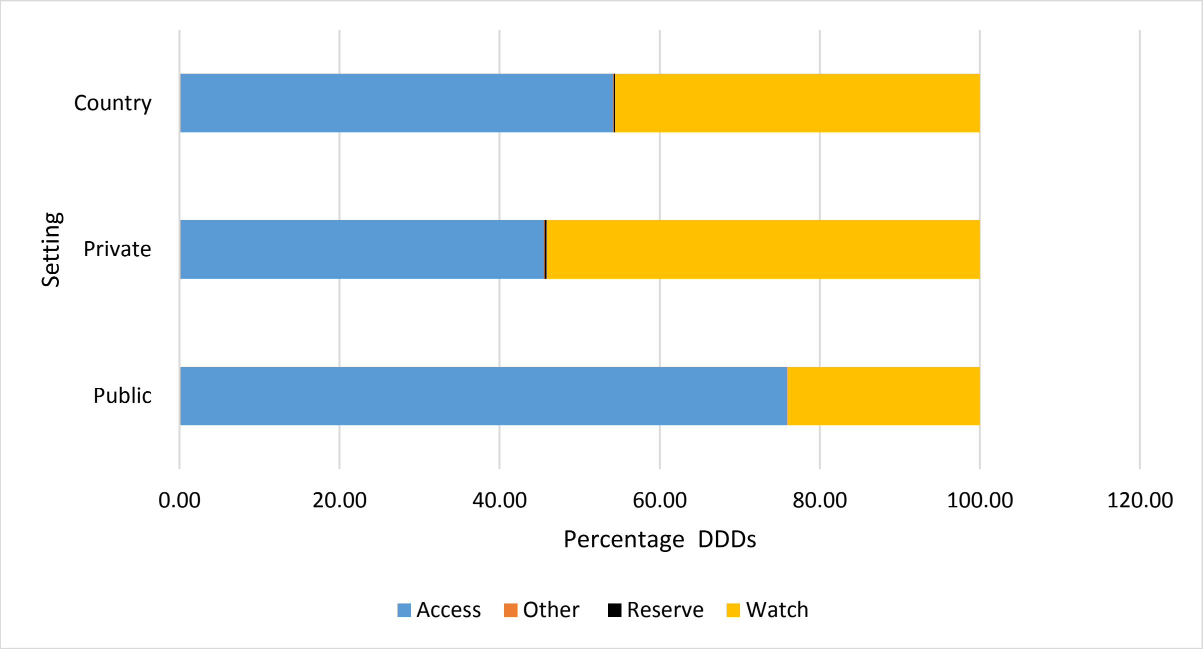

Figure 2: Top 10 ABAs, oral ABAs and parenteral ABAs consumed in public and private sector.

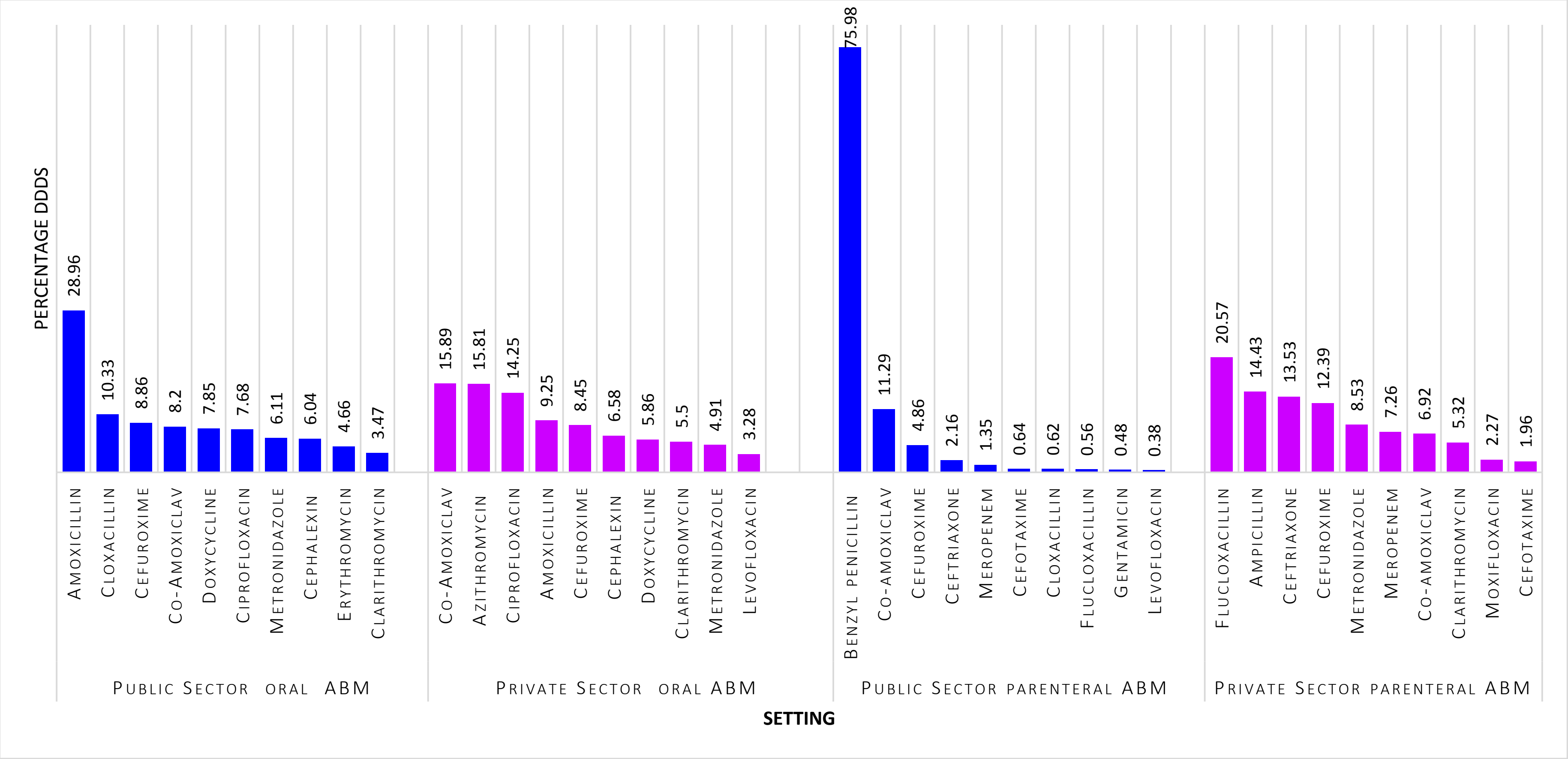

ABM- Antibacterial medicine

Discussion

Surveillance data on both resistance and consumption of antibacterials is essential to obtain a through comprehensive picture of antibiotic resistance and allows for identification of areas that need further action. While national data on antibiotic resistance patterns are available in Sri Lanka, this is the first attempt at obtaining antibiotic consumption data of the country. There is also very little data on ABC from the SEARO region (7). While we do not have complete national data, this paper presents the maximum possible extractable data on ABC in Sri Lanka for the year 2017. As recommended by the WHO for counties which are starting antimicrobial surveillance, we have used procurement/issues data available at the central level which, however, does not reflect what is actually consumed by the end user.

The total country data shows an almost similar consumption of both Access (54.23%) and Watch (45.6%) antibacterials, but a higher consumption of Watch antibacterials is seen in the private sector (54.11%) compared to the public sector (24.11%). This may also be an under representation of the consumption of Watch antibacterials in both sectors as local purchase by public sector hospitals and direct purchase from importers by private sector hospitals and retail pharmacies have not been completely captured. The disparity could be still higher as we do not have complete data from the private sector. The WHO recommends that the Watch group should be prioritized as key targets of stewardship programs and monitoring as they have higher resistance potential (15).

Despite the public sector providing the bulk of inpatient care, the ABC in the private sector was approximately three times higher than that the public sector. The SPC's supply to the private sector is almost 6 times that of to the public sector. This is despite SPC being only one

of the suppliers to the private sector while being the sole supplier to the MSD which supplies the entire public sector.

Analysing the data according to WHO’s EML and AWaRe classification (13) enabled us to understand the use of 2nd line agents. The higher consumption of Watch group antibacterials in the private sectors is also of concern as the antibiotic sensitivity patterns are the same for both sectors and practitioners in both sectors are largely the same. Lack of oversight and greater financial flexibility are possible reasons for greater use of more expensive broad-spectrum antibacterials in the private sector.

Direct comparison with other WHO regions was limited by the disparity of data sources between different regions and countries. Limited data from WHO SEARO countries show a high level of consumption of cephalosporins and quinolones in some of the countries, and a very high level of consumption of third generation cephalosporins in all States of India(7). This is similar to what was seen in Sri Lanka and an AMC pattern similar to our private sector where more antibacterials in the Watch group are consumed is also seen in several European countries and Japan (7) and in India (16, 17).

The key strength of our study is usage of standardized WHO methodology for reporting ABC in DDD and using ATC classification. These allowed for some comparison with regional and global data where available.

An important limitation of our study is the inability to capture all national data. This was largely due to incomplete and inadequate record keeping by the Customs and private importers. The inability of the SPMC to provide consumption data based on the sector to which it supplied added to the incomplete national consumption data. For meaningful interpretation of data, the total numbers of DDDs derived as consumption estimates should be adjusted for the population

to which the data apply. Since we couldn't obtain national estimate of consumption, we have not adjusted for the population. We limited the surveillance to ABAs and did not include other antimicrobials. However, recommendations from the ABAs surveillance will be applicable to AMC surveillance as well.

Conclusions

Our study, although limited to the health sector, highlights the problems a LMIC face when trying to apply globally accepted surveillance methods to determine ABC. It also highlights the need for better regulation of ABC to reduce emergence of resistance strains as we have reported disproportionally higher use of broad spectrum and "Watch category ABAs" in the private sector. Establishing a central unit to coordinate all activities related to both AMR and AMC, using accepted classification when coding imports, getting the private sector into the programme, creating a central data base which records, analyses and generates statistics routinely are some of the key recommendations for LMICs trying to establish routine antimicrobial surveillance programmes.

Acknowledgements: We acknowledge the Sri Lanka Association of Clinical Pharmacology and Therapeutics (SLACPT) for funding the data entry personnel Mr P.A.A.S.P. Kumara for assisting in data entry, Dr Malitha Rubesinghe for coordinating approvals and all the officials and pharmacists who facilitated data extraction.

The authors declare that they have no competing interests

References

1. World Health Organization. Antimicrobial resistance Global report on Surveillance 2014.
2. Goossens H, Ferech M, Vander Stichele R, Elseviers M, Group EP. Outpatient antibiotic use in Europe and association with resistance: a cross-national database study. The Lancet. 2005; 365(9459):579-87. doi:10.1016/ S0140-6736(05)17907-0
3. Costelloe C, Metcalfe C, Lovering A, Mant D, Hay AD. Effect of antibiotic prescribing in primary care on antimicrobial resistance in individual patients: systematic review and meta-analysis. Bmj. 2010;340. doi:10.1136/bmj.c2096
4. Van Boeckel TP, Gandra S, Ashok A, Caudron Q, Grenfell BT, Levin SA, et al. Global antibiotic consumption 2000 to 2010: an analysis of national pharmaceutical sales data. The Lancet infectious diseases. 2014;14(8):742-50. doi:10.1016/S1473-3099(14)70780-7
5. Klein EY, Van Boeckel TP, Martinez EM, Pant S, Gandra S, Levin SA, et al. Global increase and geographic convergence in antibiotic consumption between 2000 and 2015. Proceedings of the National Academy of Sciences. 2018;115(15):E3463-E70. doi:10.1073/pnas.1717295115
6. World Health Organization. Global Action Plan on Antimicrobial Resistance.: World Health Organization; 2015.
7. World Health Organization. WHO Report on surveillance of antibiotic consumption: 2016-2018 early implementation. 2018.
8. World Health Organization. WHO methodology for a global programme on surveillance of antimicrobial consumption. World Health Organization: 2017.
9. The world bank in Sri Lanka - "Overview ". Available from: https://www.worldbank.org/en/country/srilanka/overview.
10. World Health Organization. Antimicrobial resistance: global report on surveillance: World Health Organization; 2014.
11. World Health organization, WHO Collaborating centre for drug statistics methodology. Available: https://www.whocc.no/atc_ddd_index/.
12. Coenen S, Ferech M, Haaijer-Ruskamp FM, Butler CC, Vander Stichele RH, Verheij TJ, et al. European Surveillance of Antimicrobial Consumption (ESAC): quality

indicators for outpatient antibiotic use in Europe. BMJ Quality & Safety. 2007;16(6):440-5.
DOI: 10.1136/qshc.2006.021121

13. Sharland M, Pulcini C, Harbarth S, Zeng M, Gandra S, Mathur S, et al. Classifying antibiotics in the WHO Essential Medicines List for optimal use—be AWaRe. The Lancet Infectious Diseases. 2018;18(1):18-20.
DOI: 10.1016/S1473-3099(17)30724-7
14. World Heath organization Organization Regional office for Europe. Antimicrobial Medicines Consumption (AMC) Network. AMC data 2011–2014. World Health Organization Regional office for Europe; 2017
https://www.euro.who.int/en/publications/abstracts/antimicrobial-medicines-consumption-amc-network.-amc-data-20112014-2017
15. World Health Organization. Organization. WHO releases the 2019 AWaRe Classification Antibiotics.
https://www.who.int/medicines/news/2019/WHO_releases2019AWaRe_classification_antibiotics/en/
16. Kotwani A, Ewen M, Dey D, Iyer S, Lakshmi P, Patel A, et al. Prices & availability of common medicines at six sites in India using a standard methodology. Indian journal of medical research. 2007;125(5):645.
PMID: 17642500.
17. Farooqui HH, Selvaraj S, Mehta A, Heymann DL. Community level antibiotic utilization in India and its comparison vis-à-vis European countries: Evidence from pharmaceutical sales data. PloS one. 2018;13(10):e0204805.
doi: 10.1016/S1473- 3099(17)30724-7.
