# Supplementary material for: A national survey of antibacterial consumption in Sri Lanka
Source: PLoS One. 2021 Sep 14;16(9):e0257424. doi: 10.1371/journal.pone.0257424 (PMC8439449; doi:10.1371/journal.pone.0257424)
Supplement: Attachment — Submitted filename: Review Plos ONE _HR comments filepdf.pdf [file pone.0257424.s005.pdf]

PLOS ONE
A National Survey of antibacterial consumption in Sri Lanka
--Manuscript Draft--

**Manuscript Number:**	PONE-D-21-11873R1
**Article Type:**	Research Article
**Full Title:**	A National Survey of antibacterial consumption in Sri Lanka
**Short Title:**	National Survey of antibacterial consumption
**Corresponding Author:**	Shalini Sri Ranganathan<br>University of Colombo Faculty of Medicine<br>Colombo, SRI LANKA
**Keywords:**	antibacterials; consumption; survey; antibacterial resistance; utilization
**Abstract:**	Introduction<br>Optimizing the use of antibacterial medicines is an accepted strategy to combat the antibacterial resistance. Availability of reliable antibacterial consumption (ABC) data is a prerequisite to implement this strategy<br>Objectives<br>To quantify and describe the national ABC in Sri Lanka and to examine any differences in the consumption between public and private sector<br>Methods<br>The methodology for this survey was adapted from World Health Organization (WHO) methodology for a global programme on surveillance of antimicrobial consumption. Aggregate data on national consumption of systemic antibacterials (J01- Anatomical Therapeutic Chemical Classification (ATC) for 2017 were retrospectively extracted from all available data sources and classified using ATC classification. Quantity of consumption was converted to Defined Daily Doses (DDDs). Data are presented as total consumption and comparison between the public and private sector. We also compared few key quality indicators of ABC between these two sectors.<br>Findings<br>From the available data sources, the total ABC in 2017 was 343.46 million DDDs. Private sector consumption accounted for 246.76 million DDDs compared to 97.96 million DDDs distributed to entire public sector by the Ministry of Health. Beta-lactam-penicillins antibacterial group accounted for 58.79 % in public sector compared to 27.48 % in private sector while macrolides, quinolones and other beta-lactam antibacterials accounted for 60.51 % in the private compared to 28.41% in public sector. Consumption of Reserve group antibacterials was negligible, and limited to private sector. Watch category antibacterials accounted for 46%, 24% and 54% of the total, public and private sector consumption, respectively.<br>Conclusions<br><br>A disproportionately higher use of broad spectrum and Watch category antibacterials was observed in the private sector which needs further investigation. This national consumption survey highlights the need and provides the opening for establishment of ABC surveillance in Sri Lanka.
**Order of Authors:**	Shalini Sri Ranganathan
	Chandanie Wanigatunge
	G.P.S. G Senadheera
	BVSH Beneragama
**Response to Reviewers:**	PONE-D-21-11873<br>National surveillance of antibacterial consumption in Sri Lanka<br>Amended title: A National survey of antibacterial consumption in Sri Lanka<br>Response to Reviewers<br>COMMENTS RESPONSE FROM AUTHORS<br>ACADEMIC EDITOR<br>Please ensure that your manuscript meets PLOS ONE's style requirements, including those for file naming. The PLOS ONE style templates can be found at

https://journals.plos.org/plosone/s/file?id=wjVg/PLOSOne_formatting_sample_main_body.pdf and
https://journals.plos.org/plosone/s/file?id=ba62/PLOSOne_formatting_sample_title_authors_affiliations.pdf
Thank you very much

We apologize for not following the journal style

We have now revised the whole manuscript based on the journal style guide for title page, main body, tables, figure and references
Please provide additional details regarding participant consent. In the ethics statement in the Methods and online submission information, please ensure that you have specified (1) whether consent was informed and (2) what type you obtained (for instance, written or verbal, and if verbal, how it was documented and witnessed). If your study included minors, state whether you obtained consent from parents or guardians. If the need for consent was waived by the ethics committee, please include this information.
If you are reporting a retrospective study of medical records or archived samples, please ensure that you have discussed whether all data were fully anonymized before you accessed them and/or whether the IRB or ethics committee waived the requirement for informed consent. If patients provided informed written consent to have data from their medical records used in research, please include this information.Thank you
This is a retrospective study of medicine supplies/imports data
Ethics Review Committee of Sri Lanka Medical Association exempted this survey from ethics review committee approval (ERC-18/14). Formal request letter with a copy of ethics review committee approval was sent to all the institutions who had the data. Investigators personally visited many of these institutions to explain the aim of survey. Data presented here are from the consenting institutions. Data comprised aggregate data of amount of antibacterials distributed, imported or manufactured by these institutions and not individual patient or hospital data.
We have stated this under ethical considerations in the methods section

We note that you have indicated that data from this study are available upon request. PLOS only allows data to be available upon request if there are legal or ethical restrictions on sharing data publicly. For information on unacceptable data access restrictions, please see http://journals.plos.org/plosone/s/data-availability#loc-unacceptable-data-access-restrictions
Please see below (cover letter comment)
Cover letter
a)If there are ethical or legal restrictions on sharing a de-identified data set, please explain them in detail (e.g., data contain potentially identifying or sensitive patient information) and who has imposed them (e.g., an ethics committee). Please also provide contact information for a data access committee, ethics committee, or other institutional body to which data requests may be sent.
b)If there are no restrictions, please upload the minimal anonymized data set necessary to replicate your study findings as either Supporting Information files or to a stable, public repository and provide us with the relevant URLs, DOIs, or accession numbers. Please see http://www.bmj.com/content/340/bmj.c181.long for guidelines on how to de-identify and prepare clinical data for publication. For a list of acceptable repositories, please see http://journals.plos.org/plosone/s/data-availability#loc-recommended-repositories.
The data underlying this study belong to many third parties, Ministry of Health, State Pharmaceutical Corporation, State Pharmaceutical Manufacturing Corporation and private Pharmaceutical Companies. Raw data used to analyse antibiotic consumption in this manuscript is either state owned or private company/industry owned. Data were given to us under the agreement that the raw data will not be shared. Hence, we are unable to provide raw data in public domain. We confirm that we did not have any special access to the data that other researchers would not have.

REVIEWER 1
The manuscript addresses a timely topic which is very important for all the developing countries. It also stresses the possibility of antibiotic abuse (especially second line antibiotics) within the private sector which can be controlled with appropriate measures

	as discussed in the article. It is well written except for a few changes as per my comments. Statistically, they have only used percentages which is descriptive enough given the content of the article. Please delete the words that were highlighted in the Introduction and add the comment I stressed to the discussion.Thank you very much.<br>The typo has been deleted.<br><br>Add another limitation. The fact that you calculated the doses assuming only adults uses the antibiotics<br>Thank you for this. We have added this limitation and appropriate reference has been cited.<br>Grammatical and typo errors Thank you and have been corrected<br><br>REVIEWER 2<br>General comments<br>The manuscript needs to be reformatted by referring the journal style, excessive bolding of words and excessive unnecessary abbreviations should be reduced, and appropriate citations, repeated editing and proof reading are required. In addition to making the paper mind-numbing, those things are very technical and reduce the readability of the paper, and hence, it needs careful revisions.Thank you very much for the comment<br><br>We have carefully gone through the manuscript again and reformatted addressing the issues pointed out<br><br>We have revised the entiremanuscript and added the relevant citations<br>The background, objective, method, results and conclusions of manuscript need to be reformatted including make them bold.Thank you very much<br>We have reformatted the subheadings as per the Journal style<br>Detail comments/suggestions<br>Title<br>I suggest the title need to be revised. I think it is a cross sectional study, which is a survey, but the title saying surveillance. Unlike your study, surveillance is an ongoing collection of information to detect changes or it is repeated survey.Thank you very much for the comment<br><br>We have amended the title as<br><br>A National survey of antibacterial consumption in Sri Lanka<br>Abstract<br>The abstract lacks backgroundThank you very much<br>We have added background in the revised manuscript<br>The word WHO, in method section need to be written in full. You should first write the full term before you abbreviated. Full term do not need to be in caps and brackets, just abbreviations. Similarly for ABC in the finding section of abstract.Thank you very much<br>We have addressed this issue in the abstract as well as in the rest of the manuscript whenever applicable<br><br>The sentence “Reserve and watch category antibacterials accounted for 46, 24 and 54% of the total, public and private sector consumption” not clear and needs revisionsThank you very much<br>We apologize for the error<br>We have amended in the revised manuscript<br>Amended sentence is:<br>Watch category antibacterials accounted for 46%, 24% and 54% of the total, public and private sector consumption respectively<br>The aim of the study and the conclusion is mismatched. The aim is to describe and quantify, but the conclusion seems comparisons between public and private. The recommendation “Our study has provided the evidence that antibacterial surveillance is possible in resource limited countries and it must be made mandatory” needs revisions. Because, the recommendation is beyond the scope of the study, recommended to other resource limited countries are no appropriate, as the method including sampling procedure used in the study are not allowed to infer to other countries. I also suggest to not use the word mandatory since recommendation are not approached as obligatoryThank you very much. We agree with the comment

	We have amended the aim of the study as<br><br>To quantify and describe the national antibacterial consumption in Sri Lanka and to examine any differences in the consumption between public and private sector<br>Conclusion is amended as<br>A disproportionately higher use of broad spectrum and Watch category antibacterials was observed in the private sector which needs further investigation. This national annual consumption survey highlights the need and provides ~~has provided~~ the opening for establishment of an ABC surveillance in Sri Lanka<br>I not saw key words. Please add.We have given the key words in the online system<br>These were the key words: antibacterials; consumption; surveillance; antibacterial resistance; utilization (if possible, we will edit the surveillance as survey in the system)<br>Background<br>Revise the typo error (two point after the title) of "Introduction:"Thank you very much<br>We have corrected this typo error<br>The paper generally needs serious citation revisions. For example, in the first paragraph only, from five sites that need citations, only one has citation and the rest four are not. Please, also add more evidences/citations for those cited also.Thank you.<br>We have revised the paper extensively and added citations as appropriate<br>The third paragraph of introduction also missed citations and needs revised again.Thank you, this has been done. As we have redone the entire paper this is now paragraph 4.<br>The last paragraph of introduction "therefore conducted a national surveillance of antibacterial consumption (ABC)" needs revisions.Thank you, revised as given below<br><br>The Sri Lanka Association of Clinical Pharmacology and Therapeutics (SLACPT), in collaboration the National Focal Point for combating AMR in Sri Lanka, therefore conducted this national survey of antibacterial consumption (ABC) for 2017.<br>As suggested above to the title, this study is not surveillance rather a survey. The two concepts are quite different.Thank you very much<br>We have amended the term surveillance to survey<br>What are the significance of the sentence "This paper outlines the methods adopted, key findings and recommendations for establishment of a national surveillance programme"This sentence has been modified as below:<br><br>This paper presents the methods adopted, discusses the key findings and the problems encountered when conducting the survey.<br>The background section lacks and needs to revise based on updated prior literatures on global, regional and national picture about the antimicrobial consumption.Thank you and the background section has been extensively revised with reference to other studies on global, regional and national AMC.<br>Authors also need to add contribution of this study and practical policy implication.Thank you, we have added the following:<br><br>Our findings would be helpful when planning for comprehensive national AMC surveys or surveillances.<br><br><br>I couldn't see the aim/s and or objective of the study in this section that is mentioned in the abstract, authors need to expand those points including the rationale of the study.Thank you very much<br>We have included the objective of the study in the last paragraph of the introduction<br>Methods<br>There are many bolded disorganized subsections. No need to say background here. Please reformat them based on the journal style. For instance, study design, data source and sampling procedure in to one subsection. Variables of the study (outcome variable vs explanatory variables, including their definitions with appropriate citations, coding and categories in another subsection. Then, data entry, data analysis, and presentation of results as statistical analysis, the ethical issues in another subsection like that. Thank you very much for the comment<br><br>We have reformatted the methods section with three sub section<br>Materials and Methods

Powered by Editorial Manager® and ProduXion Manager® from Aries Systems Corporation

	1.Study design and data source<br>2.Measures of antibacterial consumption<br>3.Ethical considerations<br><br>Information given in the original manuscript had been reassigned to these three sub sections<br>Please elaborate a little more on the ethical issues. The ethical issues mentioned in the data source section need to be bring in to ethical consideration section.Thank you very much for the comment<br><br>We have given some additional information under ethical considerations<br><br>Ethics Review Committee of Sri Lanka Medical Association exempted this survey from ethics review committee approval (ERC-18/14), Formal request letter with a copy of ethics review committee approval was sent to all the institutions who had the data. Investigators personally visited many of these institutions to explain the aim of survey. Data presented here are from the consenting institutions. Data comprised aggregate data of amount of antibacterials distributed, imported or manufactured by these institutions and not individual patient or hospital data.<br>Please summarize data source and availability of data into one.Thank very much<br>Please see our responses for the first comment in the methods<br>Authors mentioned as it indicates the 2017 national antimicrobial consumption. However, the data collection period or this specific study is not mentioned.We have already given this information<br>Second sentence in the methods<br>It was done in 2018<br>I couldn't found about sampling technique and procedures use in the study. Who were the source and sampled population? How they were selected?Based on the medicine supply system in Sri Lanka, the data sources we identified are expected to provide the data for the entire country. So, we did not employ any sampling technique<br>We have now given one statement to this effect in lines 25 and 26 under methods<br><br>These data sources are expected to provide the data for the entire country. Hence, no sampling was done.<br>Who were the data collectors? What are the tools?Data comprised aggregate data They were extracted electronically (Excel worksheet) or manually transferred from the paper format of data submitted by the institutions<br>Hence we have not used data collectors.<br><br>We have now given one statement to this effect in lines 30- 32 under methods<br><br>Data from the MSD were electronically transferred. All other data sources submitted data in paper format which were manually entered in the Excel worksheet.<br>Methods- Additional change done by us<br>In the methods section we have given a formula about calculating defined daily doses. To improve the format of the section, we have converted the formula into text<br><br>Number of DDDs consumed was calculated by dividing the total consumption (in grams) by DDDs (in grams).<br>Results<br>The results need to be summarized with subsection to make clearer of the key findings.Thank you. We have added subsections as appropriate<br>Please avoid the typo error (colon after the title of resultsThank you and done<br>Results – Additional change/s done by us<br>1.We have combined results and discussion under one title as per the journal format<br>2.Additional column in table 2 - DDD per 1000s population<br>3.Additional column in table 4 to show the quality indicators for the country<br>4.Changed figure 2 to tables 5 and 6 to highlight the issues clearly<br>Discussion<br>The discussion is poor. It seems redundancy with results, and therefore, needs careful revisions; compare and contrast of your key findings with prior literatures and justify or discuss in details especially for inconsistency findings. It also have citations problem.Thank you. The discussion has been extensively revised to compare and contrast our findings with prior literature. The citations have been updated.

Powered by Editorial Manager® and ProduXion Manager® from Aries Systems Corporation

	Conclusions<br>The conclusion is not specific and not aligned with the findings. Please first write the word in full before you abbreviated it such as LMICs. You recommend to LMICS, but I couldn't see any evidence in the method sections including sampling technique, that support for generalizability of the findings for LMICs. The conclusion is beyond the study's scope and not specific. Please revise it.Thank you. We have revised the conclusions to reflect our findings.<br>REVIEWER 3<br>This is a comprehensive study looking at antibiotic consumption patterns in a LMIC. It is well-written except for a few grammatical errors (Introduction - line 3, page 5 - line 10, page 20 - lines 1 and 6). Thank you. The paper has been revised extensively and we have corrected the errors stated.<br>Data however is far from complete since many potential sources have not been able to provide usable data (ie only 12/78 private importers have submitted usable data).Agree and we have explained this in our limitations.<br>Although there were 78 importers registered with NMRA, the exact number of Companies imported antibacterials in 2017 was not available: Rough estimate from NMRA records was 40. Of them, we have got data from 12. Moreover, of the 5 leading importers, we have got data from 4. In addition, the private sector distribution data from SPC could have captured the data for the Companies who have not submitted data to us<br>We have mentioned in the results section, paragraph 1<br>A major factor that affects antibiotic consumption in the public hospitals in Sri Lanka is the highly limited formulary that is available for prescribers, compared to the private sector. This fact has not been taken in to account in the Discussion. Being able to 'locally purchase' antibiotics in the public sector does not equate to the choice of antibiotics available in the private sector.Thank you for this suggestion. We have incorporated it to the discussion as below:<br><br>A major factor that affects antibiotic consumption in the public hospitals in Sri Lanka is the highly limited formulary available to prescribers. The formulary is based on the WHO's and the country's EMLs and the medicines are made available strictly according to it to the public sector institutions. Even though prescribers have the facility of "local purchase" of medicines, this does not give them the same availability of medicines as those practicing in the private sector as the patients who seek care from these institutions are largely those with limited financial means.<br>No information on antibiotic resistance patterns in the country is provided. Therefore, the usefulness and applicability of the prescription patterns is not explored in detail.Thank you for this suggestion. We have added information on AMR patterns in Sri Lanka both in the Background and results and discussion sections.<br>Additional changes done by us<br>a)Surveillance was changed to survey in all places
**Additional Information:**	
**Question**	**Response**
**Financial Disclosure**<br><br>Enter a financial disclosure statement that describes the sources of funding for the work included in this submission. Review the submission guidelines for detailed requirements. View published research articles from *PLOS ONE* for specific examples.<br><br>This statement is required for submission and **will appear in the published article** if the submission is accepted. Please make sure it is accurate.	The author(s) received no specific funding for this work.

**Unfunded studies**<br>Enter: *The author(s) received no specific funding for this work.*<br><br>**Funded studies**<br>Enter a statement with the following details:<br>• Initials of the authors who received each award<br>• Grant numbers awarded to each author<br>• The full name of each funder<br>• URL of each funder website<br>• Did the sponsors or funders play any role in the study design, data collection and analysis, decision to publish, or preparation of the manuscript?<br>• **NO** - Include this sentence at the end of your statement: *The funders had no role in study design, data collection and analysis, decision to publish, or preparation of the manuscript.*<br>• **YES** - Specify the role(s) played.<br><br>* typeset	
**Competing Interests**<br><br>Use the instructions below to enter a competing interest statement for this submission. On behalf of all authors, disclose any competing interests that could be perceived to bias this work—acknowledging all financial support and any other relevant financial or non-financial competing interests.<br><br>This statement is required for submission and **will appear in the published article** if the submission is accepted. Please make sure it is accurate and that any funding sources listed in your Funding Information later in the submission form are also declared in your Financial Disclosure statement.<br><br>View published research articles from *PLOS ONE* for specific examples.	The authors have declared that no competing interests exist.

**NO authors have competing interests**<br><br>Enter: *The authors have declared that no competing interests exist.*<br><br>**Authors with competing interests**<br><br>Enter competing interest details beginning with this statement:<br><br>*I have read the journal's policy and the authors of this manuscript have the following competing interests: [insert competing interests here]*<br><br>* typeset	
**Ethics Statement**<br><br>Enter an ethics statement for this submission. This statement is required if the study involved:<br><br>• Human participants<br>• Human specimens or tissue<br>• Vertebrate animals or cephalopods<br>• Vertebrate embryos or tissues<br>• Field research<br><br>Write "N/A" if the submission does not require an ethics statement.<br><br>General guidance is provided below. Consult the submission guidelines for detailed instructions. **Make sure that all information entered here is included in the Methods section of the manuscript.**	Ethics Review Committee of Sri Lanka Medical Association exempted this survey from review (ERC-18/14). A formal letter of request with a copy of ethics review committee's letter of exemption was sent to all the institutions who had the data. Investigators personally visited many of these institutions to explain the aim of survey. Data presented here are from the consenting institutions. Data comprised aggregate data of the amount of antibacterials distributed, imported or manufactured by these institutions and not individual patient or hospital data.

Powered by Editorial Manager® and ProduXion Manager® from Aries Systems Corporation

**Format for specific study types**<br><br>**Human Subject Research (involving human participants and/or tissue)**<br>• Give the name of the institutional review board or ethics committee that approved the study<br>• Include the approval number and/or a statement indicating approval of this research<br>• Indicate the form of consent obtained (written/oral) or the reason that consent was not obtained (e.g. the data were analyzed anonymously)<br><br>**Animal Research (involving vertebrate animals, embryos or tissues)**<br>• Provide the name of the Institutional Animal Care and Use Committee (IACUC) or other relevant ethics board that reviewed the study protocol, and indicate whether they approved this research or granted a formal waiver of ethical approval<br>• Include an approval number if one was obtained<br>• If the study involved *non-human primates*, add *additional details* about animal welfare and steps taken to ameliorate suffering<br>• If anesthesia, euthanasia, or any kind of animal sacrifice is part of the study, include briefly which substances and/or methods were applied<br><br>**Field Research**<br><br>Include the following details if this study involves the collection of plant, animal, or other materials from a natural setting:<br>• Field permit number<br>• Name of the institution or relevant body that granted permission	
**Data Availability**<br><br>Authors are required to make all data underlying the findings described fully available, without restriction, and from the time of publication. PLOS allows rare exceptions to address legal and ethical concerns. See the PLOS Data Policy and FAQ for detailed information.	No - some restrictions will apply

A Data Availability Statement describing where the data can be found is required at submission. Your answers to this question constitute the Data Availability Statement and **will be published in the article**, if accepted.<br><br>**Important:** Stating 'data available on request from the author' is not sufficient. If your data are only available upon request, select 'No' for the first question and explain your exceptional situation in the text box.<br><br>Do the authors confirm that all data underlying the findings described in their manuscript are fully available without restriction?	
**Describe where the data may be found in full sentences. If you are copying our sample text, replace any instances of XXX with the appropriate details.**<br><br>• If the data are **held or will be held in a public repository**, include URLs, accession numbers or DOIs. If this information will only be available after acceptance, indicate this by ticking the box below. For example: *All XXX files are available from the XXX database (accession number(s) XXX, XXX.*).<br>• If the data are all contained **within the manuscript and/or Supporting Information files**, enter the following: *All relevant data are within the manuscript and its Supporting Information files.*<br>• If neither of these applies but you are able to provide **details of access elsewhere**, with or without limitations, please do so. For example:<br><br>*Data cannot be shared publicly because of [XXX]. Data are available from the XXX Institutional Data Access / Ethics Committee (contact via XXX) for researchers who meet the criteria for access to confidential data.*<br><br>*The data underlying the results presented in the study are available from (include the name of the third party*	Data cannot be shared publicly because they belong to many third parties. The data underlying the results presented in the study are available from the Medical Supplies Division, Ministry of Health (https://www.msd.gov.lk), Sri Lanka, State Pharmaceutical Corporation (https://www.spc.lk), Sri Lanka and State Pharmaceutical Manufacturing Corporation (https://www.spmclanka.lk) and private companies. Data were given to us under the agreement that the raw data will not be shared.<br>Others can access each dataset used in this study by approaching the respective institutions with letters from a recognized local ethics review committee and administrative authorities. If the required approval is obtained from the respective institutions, we confirm that others would be able to access each dataset in the same manner as the authors. We confirm that authors did not have any special access privileges to each dataset that others would not have.

and contact information or URL).<br>• This text is appropriate if the data are owned by a third party and authors do not have permission to share the data.<br><br>* typeset	
Additional data availability information:	

Powered by Editorial Manager® and ProduXion Manager® from Aries Systems Corporation

Manuscript Click here to access/download;Manuscript;Manuscript.docx

A National survey of antibacterial consumption in Sri Lanka

Shalini Sri Ranganathan[1¶,*], Chandanie Wanigatunge [2¶], G.P.S. G.Senadheera[3¶]

BVSH Beneragama[4¶]

1. Department of Pharmacology, Faculty of Medicine, University of Colombo, Sri Lanka
2. Department of Pharmacology, Faculty of Medical Sciences, University of Sri Jayewardenepura, Sri Lanka
3. Department of Pharmacy and Pharmaceutical Sciences, Faculty of Allied Health Sciences, University of Sri Jayewardenepura, Sri Lanka
4. National focal point for combating antimicrobial resistance in Sri Lanka, Ministry of Health, Sri Lanka

***** Corresponding author

Email: sshalini@pharm.cmb.ac.lk or sshalini14@hotmail.com (SS)

// Abstract

Introduction

Optimizing the use of antibacterial medicines is an accepted strategy to combat the antibacterial resistance. Availability of reliable antibacterial consumption (ABC) data is a prerequisite to implement this strategy

Objectives

To quantify and describe the national ABC in Sri Lanka and to examine any differences in the consumption between public and private sector

Methods

The methodology for this survey was adapted from World Health Organization (WHO) methodology for a global programme on surveillance of antimicrobial consumption. Aggregate data on national consumption of systemic antibacterials (J01- Anatomical Therapeutic Chemical Classification (ATC) for 2017 were retrospectively extracted from all available data sources and classified using ATC classification. Quantity of consumption was converted to Defined Daily Doses (DDDs). Data are presented as total consumption and comparison between the public and private sector. We also compared few key quality indicators of ABC between these two sectors.

Findings

From the available data sources, the total ABC in 2017 was 343.46 million DDDs. Private sector consumption accounted for 246.76 million DDDs compared to 97.96 million DDDs distributed to entire public sector by the Ministry of Health. Beta-lactam-penicillins antibacterial group accounted for 58.79 % in public sector compared to 27.48 % in private sector while macrolides, quinolones and other beta-lactam antibacterials accounted for 60.51 % in the private compared to 28.41% in public sector. Consumption of Reserve group

antibacterials was negligible, and limited to private sector. Watch category antibacterials accounted for 46%, 24% and 54% of the total, public and private sector consumption, respectively.

Conclusions

A disproportionately higher use of broad spectrum and Watch category antibacterials was observed in the private sector which needs further investigation. This national consumption survey highlights the need and provides the opening for establishment of ABC surveillance in Sri Lanka.

Introduction

Antimicrobial resistance (AMR) is a major public health challenge [1]. A global survey conducted by the World Health Organization (WHO) in 2014 has shown a high level of resistance to both first line and Reserve category antibacerials for nine pathogenic bacteria responsible for common infections in all WHO regions [2]. Infection with such resistant microorganisms result in longer illnesses, increased mortality, prolonged hospital stays and increased overall costs [1,3,4]. Antimicrobial resistance affects all areas of health including veterinary and environmental practices and impacts the entire society and its economy [2,5].

Principally driven by low-and-middle-income countries (LMICs), the global antibacterial consumption (ABC), expressed in defined daily doses (DDD), has increased by 65% from 2000 to 2015 [6]. An increased use of broad-spectrum and last-resort antibacterials was observed in both LMICs and high-income countries (HICs) during this period [6]. If the present policies continue, ABC is expected to increase by 200% in 2030 compared to the consumption in 2015 [6].

The association between ABC and development of antibacterial resistance (ABR) is well documented and a reduction of inappropriate use of antibacterials could reduce development of resistance [7,8]. Countering ABR needs long term strategies which include strengthening the healthcare systems and enacting regulations to ensure appropriate use of and access to antibacterial agents [5]. To enhance access to antibacterials for treatment of commonly occurring infections and their appropriate use, the WHO introduced the Access, Watch, and Reserve (AWaRe) classification of antibacterials as part of Essential Medicines List [9]. With the application of AWaRe classification, the WHO national level target is that 60% of the antibacterials used should be from the Access category by 2023 [9].

The WHO's Global Action Plan (GAP) for Antimicrobial Resistance (AMR) [5] calls for member states to put in place national plans to urgently combat AMR. Five strategic objectives have been identified to achieve the goals of the GAP [5]. The fourth objective viz to "Optimize the use of antimicrobial medicines in human and animal health" needs reliable antimicrobial consumption (AMC) data [5, 10]. Data on AMC are vital to understand AMR, as selection pressure due to use of antimicrobials is a preventable driver for development and spread of AMR [7, 8]. While data on AMC are collected and analysed in many high- and middle-income countries, there is limited data on AMC from lower-income countries [10]. However, the available data from LMICs show a greater increase in the use of Watch category antibacterials and a greater reduction in the Access to Watch ratio [6, 11] To effectively curtail AMR, surveillance data from AMR must be linked to that of AMC [12].

The WHO methodology for a global programme on surveillance of antimicrobial consumption provides a practical framework to obtain such data in resource limited countries (RLC) [13]. This involves the collection of "Consumption" and "Use" data and recommends that countries separate "consumption data" from "use data" as the objectives, methods and outcomes for these two categories of data are different. Consumption data" refers to estimates derived from of aggregated data, mainly derived from import, sales or reimbursement databases whereas "use data" refers to estimates derived from patient-level [13].

Sri Lanka is a lower middle-income country [14]. Both the public and the private sectors provide allopathic healthcare services in Sri Lanka but the share of care being different for inpatients and outpatients. The public sector provides the bulk of inpatient care [15] while outpatient care is shared. Infections are a leading cause of morbidity and mortality in the public sector health care institutions with zoonotic and other bacterial infections being the 2nd leading cause of death in 2017 [15] with the highest case fatality rates seen from septicaemia and pneumonia. A similar picture was seen in children where pneumonia and other bacterial

infections were the 4th and 5th leading causes of death [15]. The absence of morbidity and mortality data from the private sector makes comparisons between the sectors difficult.

The country imports the bulk of its antimicrobials through the State Pharamceuticals Corporation (SPC), which is the State's procurement arm, and independent private importers. Limited amounts of antimicrobials are manufactured by the State Pharmaceutical Manufacturing Corporation (SPMC) and individual local manufacturers. The SPC is the sole supplier of antimicrobials to the public sector. It directly imports and also procures from local manufaturers. They are distributed to medical institutions in the public sector by the Medical Supplies Division (MSD) of the Ministry of Health and when antibacterials are not available at the MSD, the individual hospitals have the option to procure them from private retail pharmacies as "local purchases". Antibacterials for the healthcare institutions in the private sector are purchased from SPC, independent private importers and local manufacturers.

Sri Lanka has an established and successful AMR surveillance programme, coordinated by the Sri Lanka College of Microbiologists, but there is no system in place to obtain aggregated AMC data. The available AMR data shows significant resistance by bacteria causing common infections to 1st line antibacterials [16-18]. Available AMC data from Sri Lanka are either limited to pharmaceutical sales data which lacks information of the public sector ABC [6, 19] or only from the state (public) sector and lacks information about consumption in the private sector [20]. The state sector data showed an increase of ABC by 143% (44.4-108.2 million DDDs) with a significant shift towards the use of broad-spectrum antibacterials from 1998 to 2018 [20]. There is, however, no system at present to correlate AMC/ABC data with AMR/ABR patterns in the country.

The Sri Lanka Association of Clinical Pharmacology and Therapeutics (SLACPT), in collaboration the National Focal Point for combating AMR in Sri Lanka, therefore conducted

this national survey of antibacterial consumption for 2017. Though the WHO methodology [13] has defined a core set of antimicrobials namely antibacterials, antibacterials for alimentary tract and nitroimidazole derivatives for protozoal diseases that all countries should monitor in their surveillance programme, this study has surveyed only the antibacterial consumption (ABC).

Our objective was to quantify and describe the national antibacterial consumption in Sri Lanka and compare the consumption between public and private healthcare sectors. This paper presents the methods adopted, discusses the key findings and the problems encountered when conducting the survey. Our findings would be helpful when planning for comprehensive national ABC/AMC surveys or surveillances.

Materials and Methods

Study design and data sources

The methodology of this study was adapted from WHO methodology for a global programme on surveillance of antimicrobial consumption [13]. It was a descriptive cross-sectional study in which aggregate data on antibacterial consumption in 2017 were retrospectively extracted from all available data sources in 2018. The WHO methodology recommends to survey antimicrobials including anti-protozoals, anti-fungals, anti-malarials and anti-virals in addition to antibacterial agents (ABAs). However, to start with, we have surveyed only the ABAs listed under antibacterials for systemic use (J01) in the Anatomical Therapeutic Chemical (ATC) classification system [21].

Local manufacturing of antibacterials is limited in Sri Lanka and the importers are the major supplier of antibacterials for the country. State Pharmaceutical Corporation is the sole importer for public sector and Rajya Osusala Pharmacies (retail pharmacy chain of SPC). It also imports for private market. In addition, there are many importers who cater for private market. Considering the supply system in Sri Lanka, we approached the Sri Lanka Customs, Department of Imports and Exports, SPC, State Pharmaceutical Manufacturing Corporation (SPMC), Medical Supplies Division (MSD), private importers and the private manufacturers (list was obtained from the NMRA website, www.nmra.gov.lk accessed on 31st August 2018) for ABC data. As these data sources are expected to provide the data for the entire country, no sampling was done. A custom-made MS Excel worksheet was developed based on WHO methodology and our previous experience in ABC surveillance in Colombo district [22]. Data from the MSD were electronically transferred. All other data sources submitted data in paper format and these were manually entered into the Excel worksheet. All the precautions were taken to ensure the accuracy of data entry. The WHO methodology recommends a detailed

product-level electronic data to be collected for ABC surveillance programmes. However, for this ~~surveillance~~, data which had the minimum details, ~~namely~~ name, dosage form, strength and quantity were considered as “complete” and included for analysis.

Measures of antibacterial consumption

The WHO defines consumption data as “estimates derived from aggregated data sources such as import or wholesaler data, or aggregated health insurance data where there is no information available on the patients who are receiving the medicines or why they are being used”(13). National antibacterial consumption (ABC) data provide a [illegible] in the country. Antibacterials for systemic use (J01, ATC classifica[illegible] were further classified to level 3, 4 and [illegible] We have presented the consumption data at level 3 (therapeutic or pharmacological sub-group) and level 5 (chemical substance). Quantity of consumption is expressed as Defined Daily Doses (DDDs) using the formula given below. The DDD is defined as “the assumed average maintenance dose per day for a medicine used for its main indication in adults”. Total grams consumed was determined by summing the amounts of active ingredient across the various formulations (different strengths of tablets or capsules, syrup formulations) and pack sizes. The DDD value is assigned by the WHO Collaborating Centre and obtained from their website (http:// www.whocc.no/atc_ddd_index/). Number of DDDs consumed was calculated by dividing the total consumption (in grams) by DDDs (in grams). Though the WHO methodology recommends that the variables for consumption estimates should include packages and DDDs, we have used only DDDs as package details were not available for this retrospective survey. In addition to presenting the total consumption data obtained from [illegible]erent data sources, we have also compared the public sector data with that of private sector, keeping in mind that the private sector data could be an underestimate as it was impossible to verify the accuracy and completeness of data. We also have compared few key ESAC based quality indicators [23] of ABC as well as the volumes of antibacterials in the “Access, Watch and Reserve” categories (AWaRe classificatio[illegible]etween these two sectors.

methodology states that data i calculated in DDDs using adult dosages. Were consumption data adjusted for syrups excluding them or keeping a percentage. What is the Sri Lankas population proportion for paediatric and geriatric patients

Ethical considerations

Ethics Review Committee of Sri Lanka Medical Association exempted this survey from review (ERC-18/14). A formal letter of request with a copy of ethics review committee's letter of exemption was sent to all the institutions who had the data. Investigators personally visited many of these institutions to explain the aim of survey. Data presented here are from the consenting institutions. Data comprised aggregate data of the amount of antibacterials distributed, imported or manufactured by these institutions and not individual patient or hospital data.

Results and discussion

We analyzed the data from the MSD, SPC, one local manufacturer (SPMC) and 12 private importers. Four local manufacturers did not provide data. Although 78 are registered as private importers with the NMRA, the exact number of Companies actually imported antibacterials in 2017 was not available. The rough estimate from the NMRA records was 40 and 12 of them provided analyzable data. Four out of the 5 leading importers provided analyzable data whilst data provided by one major importer had to be discarded as the Company did not provide the information on strengths of the dosage forms which is essential to calculate DDD. The MSD data is reliable and is the almost complete data for public sector. A very small proportion of antibacterials purchased by individual hospitals as "local purchase" would not have been captured in our data. The SPC provided data separately for public and private sector while SPMC provided cumulative data for both sectors. Data provided by the private importers is their imports for private sector. The SPC data for private sector may well include the data for the Companies who have not provided their imports data to us

The total volume of antibacterial agents (ABA) (in million DDDs) imported/distributed in 2017 by the respective agencies is shown in Table 1.

Table 1: Total volume of ABAs (in million DDDs) imported/distributed in 2017 by the different agencies

Agency	Volume of ABAs (in million DDDs)
1. Distributed by SPC to private sector	163.04
2. Distributed by MSD to public sector [1]	97.96
3. Distributed by SPMC to both to public and private sector[2]	61.18
4. Imported by private sector	59.30
5. Manufactured by SPMC [2]	56.08
6. Distributed by SPC to MSD [1]	31.43
7. Distributed by SPC to its retail pharmacies (Rajya Osusala)	24.41

1. *MSD distributed to public sector is higher than the SPC distributed to MSD because left over stocks from 2016 could have been distributed by the MSD.*
2. *Manufactured amount is not equal to distributed amount because all the amount manufactured in a particular year would not have been distributed in that particular year*

Despite most of the inpatient care being provided by the public sector health care institutions, the ABC in the private sector (Table 1; data sources 1, 4 and 7) accounted for 246.76 million DDDs compared to 97.96 million DDDs distributed to the public sector by the MSD (data source 2).

The SPC was initially established as a procurement agency for the public sector. However, in 2017 a significant 74.5% of its imports had been for the private sector (163 million DDDs,) while it was only 14.35% to the public sector (31.43 million DDDs)

Comparison of ABC between public and private sectors

As Sri Lanka has a free public health care system and a fee levying private health care system, we analyzed the data according to the total ABC and in the two sectors. The top four groups of antibacterials consumed in Sri Lanka were beta-lactams, penicillins, other beta-lactams, macrolides and quinolones (Table 2). However, major differences were observed in the

proportion of volumes consumed with these categories between the public and private sectors. The beta-lactam antibacterials, penicillins considerably outnumbered (58.57%) the other three groups (13.7%, 7.8%, and 6.8%) in the public sector whereas each of the top four groups accounted for between 15-36% of the ABC in the private sector. Compared to the public sector, the consumption of macrolides, quinolones and other beta lactam antibacterials is disproportionately higher in the private sector.

Table 2: Consumption of different pharmacological sub-groups of antibacterials in the country and public and private sectors

ATC code	ATC level 3 classification of ABAs	ABA consumption volume in DDD per million						DDD per 1000s population
		Public sector (%)		Private sector (%)		Total (%)		
J01A	Tetracycline's	5.94	(6.06)	14.89	0.01	20.83	(6.04)	0.98
J01B	Amphenicols	0.00	(0.00)	0.19	5.90	0.19	(0.06)	0.01
J01C	Beta-lactam antibacterials, penicillins	57.38	(58.57)	67.80	2.62	125.18	(36.31)	5.90
J01D	Other Beta-lactam antibacterials	13.38	(13.66)	42.20	0.10	55.58	(16.12)	2.62
J01E	Sulfonamides and trimethoprim	0.50	(0.51)	1.71	3.22	2.21	(0.64)	0.10
J01F	Macrolide, lincosamide and streptogramins	7.69	(7.78)	60.57	0.01	68.26	(19.80)	3.22
J01G	Aminoglycoside antibacterials	0.14	(0.14)	0.00	2.51	0.14	(0.04)	0.01
J01 M	Quinolone antibacterials	6.66	(6.82)	46.57	0.90	53.23	(15.44)	2.51
J01X	Other antibacterials	6.28	(6.41)	12.84	16.26	19.12	(5.55)	0.90
	**Total**	**97.96**	**100.00**	**246.76**	**0.98**	**344.72**	**100.00**	**16.26**

Reference: Mid-Year Population in 2017 (estimated) – 21,203,000
Mid-year population estimate by district and sex 2014-2019
http://www.statistics.gov.lk/PopHouSat/VitalStatistics/MidYearPopulation/Mid-year%20population%20by%20district.pdf

The consumption of most frequently consumed antibacterials (5th and last level of ATC classification) within these top four groups are shown in table 3. (Detailed data are available in the supplementary Table 1). Substantial differences were observed between public and private

sectors in the consumption of individual antibacterials within each of the top four groups. In the private sector co-amoxiclav was the most consumed antibacterial in J01C group and azithromycin in the J01F group while amoxicillin and erythromycin were the equivalents in the public sector. Interestingly, benzyl penicillin was consumed only in the public sector.

Table 3: Most consumed antibacterials of the top four pharmacological groups in private and public sectors

ATC level 5 classification of ABAs (Code) under each level 3 classification	Name of classes and individual ABAs	Public sector (%)		Private sector (%)	
		DDDs	(%)*	DDDs	(%)*
J01C	Beta-lactam antibacterials, Penicillins				
J01CA04	Amoxicillin	21.91	(22.37)	22.66	(9.18)
J01CE01	Benzyl penicillin	16.95	(17.30)	-	-
J01CF02	Cloxacillin	7.96	(8.13)	2.49	(1.01)
J01CF05	Flucloxacillin	0.32	(0.33)	1.93	(0.78)
J01CR02	Co-Amoxiclav	8.72	(8.90)	39.07	(15.83)
J01D	Other Beta-lactam antibacterials				
J01DB01	Cephalexin	4.57	(4.67)	16.12	(6.53)
J01DC02	Cefuroxime	7.79	(7.95)	20.91	(8.47)
J01DD08	Cefixime	0.05	(0.05)	4.59	(1.86)
J01F	Macrolide, Lincosamide and Streptogramins				
J01FA01	Erythromycin Stearate	3.52	(3.59	6.13	(2.49)
J01FA09	Clarithromycin	2.67	(2.73)	13.58	(5.50)
J01FA10	Azithromycin	1.26	(1.29)	38.74	(15.70)
J01M	Quinolone antibacterials				
J01MA01	Ofloxacin	0.03	(0.03)	0.32	(0.13)
J01MA02	Ciprofloxacin	5.81	(5.93)	34.93	(14.16)
J01MA06	Norfloxacin	0.52	(0.53)	2.76	(1.12)
J01MA12	Levofloxacin	0.22	(0.22)	8.05	(3.26)

* *Percentages are out of total antibacterials consumed in the respective sector. Total is not 100 as only four classes are shown here (see Supplementary Table 1 for full details)*

Quality indicators of ABC for the country as a whole and separately for public and private sectors are given in Table 4.

Table 4: Comparison of few key quality indicators of ABC between public and private sector

	Indicator	Public sector	Private sector	Total *
J01CE_%	Consumption of β-lactamase sensitive penicillins (J01CE) expressed as percentage of the total consumption of antibacterials for systemic use (J01)	18.77	0.18	5.46
J01CR_%	Consumption of combination of penicillins, including β -lactamase inhibitor (J01CR) expressed as percentage of the total consumption of antibacterials for systemic use (J01)	8.98	15.83	13.89
J01DD+DE_%	Consumption of third and fourth generation of cephalosporins (J01(DD+DE)) expressed as percentage of the total consumption of antibacterials for systemic use (J01)			
	1st Generation	4.67	6.55	6.01
	2nd Generation	7.95	8.52	8.35
	3rd Generation	0.72	1.98	1.62
	4th Generation	0.00	0.00	0.00
J01MA_%	Consumption of flouroquinolones (J01MA) expressed as percentage of the total consumption of antibacterials for systemic use (J01)	6.71	18.81	15.31
J01_B/N	Ratio of the consumption of broad (J01(CR+DC+DD+(F-FA01))) to the consumption of narrow spectrum penicillins, cephalosporins and macrolides (J01(CE+DB+FA01))	0.81	5.25	2.86

** Indicators are calculated for total data to show how one sector affects the country data*

Antibacterials consumption according to WHO AWaRe Classification

Fig 1 shows the consumption of antibacterials in the "Access, Watch and Reserve" categories for the country as a whole and separately for public and private sector (supplementary Table 2). Of the total antibacterials consumed, 54.19% were from the Access category while 45.57 were from the Watch group with the Access: Watch ratio was 1.18. However, in the public sector this ratio was 3.16 while it was 0.84 in the private sector.

Fig 1: Consumption of antibacterials in Access, Watch and Reserve categories

The single most important difference observed between public and private sector (Tables 2,3 and 4, Fig 1) was disproportionately higher use of broad spectrum antibacterials in the private sector. We compared the top ten oral and parental antibacterials used between the sectors (Tables 5,6). Both sectors have consumed large amounts of antibacterials in the Watch category and this is more in the private sector. A notable difference was the higher use of parenteral metronidazole and meropenem in private sector compared to that of public sector.

Table 5: Comparison of the top ten oral antibacterials consumed between sectors

Public sector				Private Sector			
ABM	AwaRe group	DDDs in million	%	ABM	AwaRe group	DDDs in million	%
Amoxicillin	A	21.91	28.96	Co-Amoxiclav	A	38.95	15.89
Cloxacillin	A	7.82	10.33	Azithromycin	W	38.74	15.81
Cefuroxime	W	6.7	8.86	Ciprofloxacin	W	34.91	14.25
Co-Amoxiclav	A	6.2	8.2	Amoxicillin	A	22.66	9.25
Doxycycline	A	5.94	7.85	Cefuroxime	W	20.7	8.45
Ciprofloxacin	W	5.81	7.68	Cephalexin	A	16.12	6.58
Metronidazole	A	4.62	6.11	Doxycycline	A	14.35	5.86
Cephalexin	A	4.57	6.04	Clarithromycin	W	13.49	5.5
Erythromycin	W	3.52	4.66	Metronidazole	A	12.03	4.91
Clarithromycin	W	2.62	3.47	Levofloxacin	W	8.03	3.28

A= Aware *W= Watch*

Table 6: Comparison of the top ten parental antibacterials consumed between sectors

Public sector				Private sector			
ABM	AwaRe group	DDDs in million	%	ABM	AwaRe group	DDDs in million	%
Benzyl penicillin	A	16.95	75.98	Flucloxacillin	A	0.35	20.57
Co-amoxiclav	A	2.52	11.29	Ampicillin	A	0.24	14.43
Cefuroxime	W	1.08	4.86	Ceftriaxone	W	0.23	13.53
Ceftriaxone	W	0.48	2.16	Cefuroxime	W	0.21	12.39
Meropenem	W	0.3	1.35	Metronidazole	A	0.14	8.53
Cefotaxime	W	0.14	0.64	Meropenem	W	0.12	7.26
Cloxacillin	A	0.14	0.62	Co-amoxiclav	A	0.12	6.92
Flucloxacillin	A	0.13	0.56	Clarithromycin	W	0.09	5.32
Gentamicin	A	0.11	0.48	Moxifloxacin	W	0.04	2.27
Levofloxacin	W	0.09	0.38	Cefotaxime	W	0.03	1.96

A= Aware *W= Watch*

Surveillance data on both resistance and consumption are essential to obtain a comprehensive picture of antibacterial resistance. Correlating ABC data with the ABR patterns will help to identify areas that need further action. While national data on ABR patterns are available in Sri Lanka [24,25], to the best of our knowledge this is the first attempt at obtaining national antibacterial consumption data. Previous data from Sri Lanka which have been included in other surveys [6,19,20] have been obtained from either only pharmaceutical sales (6,19) data or only public sector ABC data (20). Adopting WHOs' standard methodology made it possible for us to compare our findings with similar studies done globally and in the region.

We compared Sri Lanka's ABC in 2017 with global surveys on ABC [6, 11] and that of the ESAC-Net countries (26). In 2015, the most commonly consumed antibacterial classes globally were broad-spectrum penicillins (J01CA), cephalosporins (J01D), quinolones (J01M) and macrolides (J01F) [6,11]. This was similar to the top four groups of antibacterials consumed in Sri Lanka. The average total ABC for systemic use (ATC group J01) in the EU/EEA (23.4 DID) [26] was much higher than that of Sri Lanka for 2017 (16.26 DID). A key finding was the ratio of the consumption of broad spectrum to narrow spectrum penicillins, cephalosporins and macrolides (J01_B/N). This was 2.86 for the country, 0.81 for the public sector but a significant 5.25 for the private sector (Table 4). This ratio is higher than what was seen in the community consumption for ESAC-Net countries i.e. 2.25 [26]. The higher DID and the comparatively lower J01-B/N in ESAC-Net countries could be due to greater access to and better regulation of antimicrobials in these countries.

Direct comparison with other WHO South East Asian Region (SEAR) countries in the region was limited by the lack of studies and the disparity of data sources in different studies. Although South East Asian Region (SEAR) was excluded in the WHO report on surveillance

of antibacterial consumption from 2016 – 2018 due to the lack of data, a high level of consumption of cephalosporins and quinolones was found in some countries of the region [2]. A higher consumption of other beta-lactam antibacterials, macrolides and quinolones was seen in the private sector compared to that of the public sector of Sri Lanka. This was somewhat similar to that of the retail private sector in India which also showed an increase in the use of 3rd generation cephalosporins, penicillins with beta-lactamase inhibitors, and of newer classes of antibacterials like carbapenems, lincosamides and glycopeptides [27].

As the WHO recommends that the Watch group should be prioritized as key targets of stewardship programs and monitoring as they have higher resistance potential [9], we analysed the data according to WHO's AWaRe classification (9). Despite a 54.19% overall use of Watch antibacterials in the country, a higher consumption of these antibacterials was seen in the private sector (54.11%) compared to the public sector (24.11%) (Fig 1).This may be an under representation of the consumption of Watch antibacterials in both sectors as local purchase by public sector hospitals and direct purchase from importers by private sector hospitals and retail pharmacies have not been completely captured in our data. The disparity could be still higher as we do not have complete data from the private sector imports. A similar pattern where more antibacterials in the Watch group are consumed is also seen in several European countries and Japan [10] and in India [16, 28]. The higher consumption of Watch group antibacterials in the private sector in Sri Lanka is a concern as the antibacterial sensitivity patterns are the same for both sectors and practitioners in both sectors are largely the same. It is more alarming as there is an increasing emergence of multi drug resistant bacteria in the country [16-18,29]. It has also been shown that some of the pathogens causing lower respiratory tract infections are resistant to the first line Access antibacterials but show

an increased sensitivity to 2nd line (Watch) agents [30]. Infections with these resistant bacteria would add a significant burden to the health budget.

Lack of regulatory oversight, greater accessibility to antibacterials and improved financial status of the patients are possible reasons for greater use of newer and more expensive broad-spectrum antibacterials in the private sector. A major factor that affects antibacterial consumption in the public hospitals in Sri Lanka is the highly limited formulary available to prescribers. The formulary is based on the WHO's and the country's EMLs and the medicines are made available strictly according to it to the public sector institutions. Even though prescribers have the facility to "local purchase" medicines, this does not give them the same availability of medicines as those practicing in the private sector as the patients who seek care from these institutions are largely from lower income segments of the society. However, inappropriate prescriptions of antibacterials are seen in the public sector hospitals too [31,32] and was associated with incorrect physician perception of the need for antibacterials, high patient volume and fear of bacterial super infection [32].

The Government of Sri Lanka has initiated many regulatory mechanisms to curtail inappropriate antibacterial use. All antibacterials are registered under Schedule 11B by the National Medicines Regulatory Authority and are prescription only [33]. Despite such regulations it is still possible to obtain antibacterials from retail pharmacies without a valid prescription [34,35]. Self-medication of antibacterials by patients [35] and the freedom to access any practitioner without an appropriate referral which could lead to duplication and/or inappropriate prescriptions, also contribute to an increase in ABC and AMR in the country. While we do not have complete national data, this paper presents the maximum possible extractable data on ABC in Sri Lanka for the year 2017. As recommended by the WHO for

counties which are starting antimicrobial surveillance, we have used procurement/issues data available at the central. This, however, does not reflect what is actually consumed by the end user. The key strength of our study is usage of standardized WHO methodology for reporting ABC in DDD and using ATC classification. The DDD methodology allowed us to use aggregated antibacterial purchase data and made it possible to compare our data with regional and global data. However, the DDD may not reflect the prescribed daily dose (PDD) for individual patients and cannot be used to measure consumption in paediatric wards since the measure is based on adult dosing [36]. It also does not accurately measure antibacterial consumption in cases of renal or hepatic dysfunction, often underestimating the actual antibacterial usage [36].

An important limitation of our study was the inability to capture all national data. This was largely due to incomplete and inadequate record keeping by the Customs and private importers. The inability of the SPMC to provide consumption data based on the sector to which it supplied added to the incomplete national consumption data. For meaningful interpretation of data, the total numbers of DDDs derived as consumption estimates should be adjusted for the population to which the data apply. Despite these limitations we have adjusted for the population (DID) to compare with similar studies.

Conclusions

Despite limitations our study provides the first national ABC data from Sri Lanka which captures the use in both public and private sectors. Although limited to the health care sector, it highlights the problems a LMIC face when trying to apply globally accepted survey methods to evaluate aspects of ABC. Establishing a central unit to coordinate all activities related to both AMR and AMC, using accepted classification when coding imports, getting

the private sector into the programme and creating a central data base which records, analyses and generates statistics routinely are key areas that need attention for better surveillance of antimicrobial use. As antibacterials use is to some extent patient and prescription driven in Sri Lanka, education campaigns targeting both patients and prescribers are needed to change behaviours and prescribing habits. Better implementation of existing regulation is vital to curtail antibacterial misuse.

Acknowledgements: We acknowledge the Sri Lanka Association of Clinical Pharmacology and Therapeutics (SLACPT) for funding the data entry personnel Mr P.A.A.S.P. Kumara for assisting in data entry, Dr Malitha Rubesinghe for coordinating approvals and all the officials and pharmacists who facilitated data extraction.

The authors declare that they have no competing interests

References

1. Laxminarayan R, Duse A, Wattal C, Zaidi AKM, Wertheim HFL, Sumpradit N, et al. Antibacterial resistance-the need for global solutions. The Lancet Infectious Diseases, 2013; 13(12), 1057-1098. doi: 10.1016/S1473-3099(13)70318-9
2. World Health Organization. Antimicrobial resistance: Global report on Surveillance 2014.
3. Centers for Disease Control and Prevention. Antibacterial Resistance Threats in the United States, 2019. Atlanta, GA: U.S. Department of Health and Human Services, CDC; 2019. Available at https://www.cdc.gov/drugresistance/pdf/threats-report/2019-ar-threats-report-508.pdf. Accessed 18th May 2021
4. Ventola CL. "The antibacterial resistance crisis: part 1: causes and threats." P & T : a peer-reviewed journal for formulary management vol. 40,4 (2015): 277-83.
5. World Health Organization. Global Action Plan on Antimicrobial Resistance: World Health Organization; 2015.
6. Klein EY, Van Boeckel TP, Martinez EM, Pant S, Gandra S, Levin SA, et al. Global increase and geographic convergence in antibacterial consumption between 2000 and 2015. Proceedings of the National Academy of Sciences. 2018;115(15):E3463-E70. doi:10.1073/pnas.1717295115
7. Goossens H, Ferech M, Vander Stichele R, Elseviers M, Group EP. Outpatient antibacterial use in Europe and association with resistance: a cross-national database study. The Lancet. 2005; 365(9459):579-87. doi:10.1016/ S0140-6736(05)17907-0
8. Costelloe C, Metcalfe C, Lovering A, Mant D, Hay AD. Effect of antibacterial prescribing in primary care on antimicrobial resistance in individual patients: systematic review and meta-analysis. Bmj. 2010;340. doi:10.1136/bmj.c2096
9. World Health Organization model list of essential medicines, 21st List, 2019. Geneva: World Health Organization, 2019.
10. World Health Organization. WHO Report on surveillance of antibacterial consumption: 2016-2018 early implementation. 2018.

11. Versporten A, Zarb P, Caniaux I, Gros MF, Drapier N, Miller M, et al. Global-PPS network. Antimicrobial consumption and resistance in adult hospital inpatients in 53 countries: results of an internet-based global point prevalence survey. Lancet Glob Health. 2018; 6(6):e619-e629. doi: 10.1016/S2214-109X(18)30186-4.
12. Surveillance and monitoring for antimicrobial use and resistance, IAGC discussion paper, June 2018. Available at https://www.who.int/antimicrobial-resistance/interagency-coordination-group/IACG_Surveillance_and_Monitoring_for_AMU_and_AMR_110618.pdf Accessed on 18th May 2021
13. World Health Organization. WHO methodology for a global programme on surveillance of antimicrobial consumption. World Health Organization: 2017.
14. The world bank in Sri Lanka -Overview. Available from: https://www.worldbank.org/en/country/srilanka/overview. Accessed 18th May 2021
15. Annual Health Bulletin 2018, Ministry of Health, Sri Lanka Available at: http://www.health.gov.lk/moh_final/english/public/elfinder/files/publications/AHB/2020/AHB_2017.pdf. Accessed 18th May 2021
16. Jayatilleke S, Patabedige G, Dassanayake M. Analysis of urine culture isolates from seven laboratories of Sri Lanka: National Laboratory Based Surveillance of Sri Lanka College of Microbiologists in 2014. Sri Lankan Journal of Infectious Diseases. 2016;6(1):17–24. doi: http://doi.org/10.4038/sljid.v6i1.8105
17. Perera V, Assellage P and De Silva P. Profile of extended spectrum βlactamase (ESBL) producing enterobacteria isolated from clinical specimens in a private teaching hospital in Sri Lanka. The Bulletin of the Sri Lanka College of Microbiologists. 2014; 12(1):19-20
18. Fernando MMPSC, Luke WANV, Miththinda JKND, Wickramsingha RDSS, Sebastiampillai BS, Gunathilake MPML et al. Extended spectrum beta lactamase producing organisms causing urinary tract infections in Sri Lanka and their antibacterial susceptibility pattern –A hospital based cross sectional study. BMC Infect Dis. 2017:17, 13.doi:10.1186/s12879-017-2250-y

19. Klein EY, Milkowska-Shibata M, Tseng KK, Sharland M, Gandra S, Pulcini C, Laxminarayan R. Assessment of WHO antibacterial consumption and access targets in 76 countries, 2000–15: an analysis of pharmaceutical sales data. The Lancet Infectious Diseases, 2021;21(1), 107-115. doi:10.1016/S1473-3099(20)30332-7

20. Fernandopulle BMR, Yasarathna KWGKP, Gunasekara ADM, Wijesinghe KGCD, Beneragama BVSH, Panapitiya L et al. Antibacterial Consumption in the State Sector of Sri Lanka Over 25 Years: 1994 - 2018 Using the GAP Methodology. The Pharmaceutical Journal of Sri Lanka 2020 10(1): 54-63. doi: 10.4038/pjsl.v10i1.57

21. World Health organization, WHO Collaborating centre for drug statistics methodology. Available: https://www.whocc.no/atc_ddd_index/.

22. Senadheera GPSG, Sri Ranganathan S, Fernando GH, Fernandopulle BMR. Antibacterial Medicine Consumption in Private and State Sector Outpatient Settings in Colombo District, Sri Lanka. Pharmaceutical Journal of Sri Lanka. 2019;9(1):28–39. DOI: http://doi.org/10.4038/pjsl.v9i1.49

23. Coenen S, Ferech M, Haaijer-Ruskamp FM, Butler CC, Vander Stichele RH, Verheij TJ et al.; ESAC Project Group. European Surveillance of Antimicrobial Consumption (ESAC): quality indicators for outpatient antibacterial use in Europe. Qual Saf Health Care. 2007 Dec;16(6):440-5. doi: 10.1136/qshc.2006.021121.

24. JayatillekeS, Patabedige G, Dassanayake M. Analysis of urine culture isolates from seven laboratories of Sri Lanka: National Laboratory Based Surveillance of Sri Lanka College of Microbiologists in 2014. Sri Lankan Journal of Infectious Diseases, 2016;6(1):17–24. doi: 10.4038/sljid.v6i1.8105

25. ARSP Working Group, The Sri Lanka College of Microbiologists. A multi centre laboratory study of Gram negative bacterial blood stream infections in Sri Lanka. Ceylon Med J. 2013 Jun;58(2):56-61. doi: 10.4038/cmj.v58i2.5680

26. European Centre for Disease Prevention and Control. Antimicrobial consumption. In: ECDC. Annual epidemiological report 2017. Stockholm: ECDC; 2018.

27. Farooqui HH, Selvaraj S, Mehta A, Heymann DL. Community level antibacterial utilization in India and its comparison vis-à-vis European countries: Evidence from

pharmaceutical sales data. PLoS ONE; 2018:13(10): e0204805. doi:10.1371/journal.pone.0204805

28. McGettigan P, Roderick P, Kadam A, Pollock AM. Access, Watch, and Reserve antibacterials in India: challenges for WHO stewardship. Lancet Glob Health. 2017;5(11):e1075-e1076. doi: 10.1016/S2214-109X(17)30365-0.

29. Kumuduni WGM, Wijesooriya LI, Namalie KD, Sunil-Chandra NP, Wijayasinghe YS. Epidemiology of multidrug-resistant Enterobacteriaceae in Sri Lanka: First evidence of blaKPC harboring Klebsiella pneumoniae. Journal of Infection and Public Health; 2020:13(9), 1330-1335. doi: 10.1016/j.jiph.2020.04.010.

30. Amarasinghe N, Athavan M, Jayamanne D, Rajapakshe Y, Sadikeen A, Gunasekera K et al. Bacterial profile and antibacterial susceptibility pattern of adult lower respiratory tract infections in Colombo, Sri Lanka. Journal of Health and Social Sciences 2018; 3,1:27

31. Zhang HL, Bodinayake C, Wijayaratne GB, Jayatissa P, Piyasiri B, Kurukulasooriya R et al. Point-prevalence survey of outpatient antibacterial prescription at a tertiary medical center in Sri Lanka: opportunities to improve prescribing practices for respiratory illnesses. BMC Infect Dis; 2021:21, 97). Doi:10.1186/s12879-021-05804-6

32. Tillekeratne LG, Bodinayake CK, Dabrera T, Nagahawatte A, Kodikara Arachchi W, Sooriyaarachchi A. et al. Antibacterial overuse for acute respiratory tract infections in Sri Lanka: a qualitative study of outpatients and their physicians. BMC Fam Pract; 2017: 18, 37. doi:10.1186/s12875-017-0619-z

33. National Medicines Regulatory Authority Act, No. 5 of 2015. Available at https://nmra.gov.lk/images/PDF/gazzet/PG--4687--E.pdf. Accessed on 18th May 2021

34. Zawahira S, Lekamwasam S, Aslania P. Antibacterial dispensing practice in Sri Lankan community pharmacies: Asimulated client study. Research in Social and Administrative Pharmacy; 2019: 15(5), 584-590

35. Senadheera GPSG, Sri Ranganathan S, Fernando GH, Fernandopulle BMR. Practice of self-medication with antibacterials in the Colombo district, Sri Lanka. Ceylon Medical Journal 2017; 62: 70-72. doi:10.4038/cmj.v62i1.8439

36. Gravat LAH, Pakyz AL. Challenges in Measuring Antibacterial Consumption. Curr Infect Dis Rep. 2013; 15:559–563. doi: 10.1007/s11908-013-0374-9

 Click here to access/download;Figure;Figure 1.tiff

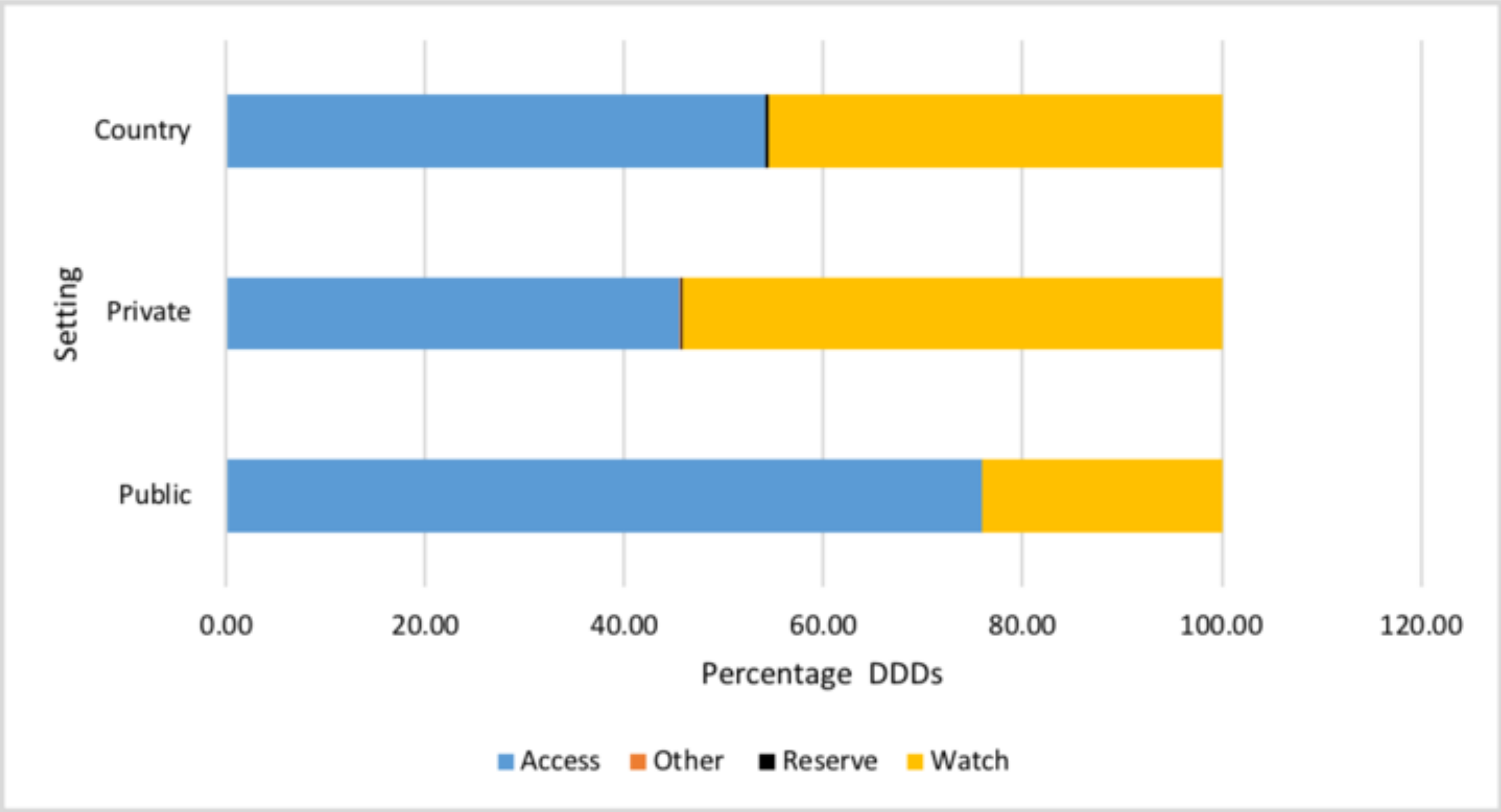

Click here to access/download
Supporting Information
Supplementary Table 1.docx

Click here to access/download
Supporting Information
Supplementary Table 2.docx

~~National surveillance of antibacterial consumption in Sri Lanka~~

Formatted: Width: 8.26", Height: 11.69", Header distance from edge: 0.49", Footer distance from edge: 0.49"

Title: **National surveillance of antibacterial consumption in Sri Lanka**

Authors: Shalini Sri Ranganathan[1,5], Chandanie Wanigatunge [2,5], G.P.S. G.Senadheera[3,] BVSH Beneragama[4]

Institutions

1. Department of Pharmacology, Faculty of Medicine, University of Colombo, Sri Lanka
2. Department of Pharmacology, Faculty of Medical Sciences, University of Sri Jayewardenepura, Sri Lanka
3. Department of Pharmacy and pharmaceutical Sciences, Faculty of Allied Health Sciences, University of Sri Jayewardenepura, Sri Lanka
4. Deputy Director General (Laboratory Services), National focal point for combating antimicrobial resistance in Sri Lanka, Ministry of Health
5. Sri Lanka Association for Clinical Pharmacology and Therapeutics (SLACPT)

Corresponding author

Name: Shalini Sri Ranganathan

Address: Department of Pharmacology, Faculty of Medicine, University of Colombo, Sri Lanka

Email sshalini@pharm.cmb.ac.lk or sshalini14@hotmail.com

Any disclaimers: None

Sources of support: Sri Lanka Association of Clinical Pharmacology and Therapeutics provided funds for data entry.

Word count: 2572 (excluding abstract, acknowledgements, references, tables and figures)

Number of tables= 4

Number of figures = 2

Formatted: Normal, Left, Space After: 8 pt, Line spacing: Multiple 1.08 li

Formatted: Normal, Space After: 8 pt, Line spacing: Multiple 1.08 li

A National survey of antibacterial consumption in Sri Lanka

Shalini Sri Ranganathan[1¶,*], Chandanie Wanigatunge [2¶], G.P.S. G.Senadheera[3¶]

BVSH Beneragama[4¶]

1. Department of Pharmacology, Faculty of Medicine, University of Colombo, Sri Lanka
2. Department of Pharmacology, Faculty of Medical Sciences, University of Sri Jayewardenepura, Sri Lanka
3. Department of Pharmacy and Pharmaceutical Sciences, Faculty of Allied Health Sciences, University of Sri Jayewardenepura, Sri Lanka
4. National focal point for combating antimicrobial resistance in Sri Lanka, Ministry of Health, Sri Lanka

***** Corresponding author

Email: sshalini@pharm.cmb.ac.lk or sshalini14@hotmail.com (SS)

Formatted: Numbered + Level: 1 + Numbering Style: 1, 2, 3, … + Start at: 1 + Alignment: Left + Aligned at: 0.19" + Indent at: 0.44"

Formatted: Normal, Space After: 8 pt, Line spacing: Multiple 1.08 li

Formatted: Font: Not Bold

Formatted: Normal, Left, Space After: 8 pt, Line spacing: Multiple 1.08 li

Formatted: Font: Not Bold

Formatted: Normal, Space After: 8 pt, Line spacing: Multiple 1.08 li

Abstract

Formatted: Font: 18 pt

Introduction

Optimizing the use of antibacterial medicines is an accepted strategy to combat the antibacterial resistance. Availability of reliable antibacterial consumption (ABC) data is a prerequisite to implement this strategy

Objectives~~:~~

To quantify and describe the national ABC ~~antibacterial consumption~~ in Sri Lanka and to examine any differences in the consumption between public and private sector

Methods~~:~~

The methodology for this survey was adapted from World Health Organization (WHO) methodology for a global programme on surveillance of antimicrobial consumption. Aggregate data on national consumption of systemic antibacterials (J01- Anatomical Therapeutic Chemical Classification (ATC)~~)~~ for ~~in~~ 2017 were retrospectively extracted from all available ~~various~~ data sources and classified using ATC classification. Quantity of consumption was converted to Defined Daily Doses (DDDs). Data are presented as total consumption and comparison between the public and private sector. We also compared few key quality indicators of ~~antibacterial consumption~~ ABC between these two sectors.

Findings~~:~~

From the available data sources, the total ABC in 2017 was 343.46 million DDDs. Private sector consumption accounted for 246.76 million DDDs compared to 97.96 million DDDs distributed to entire public sector by the Ministry of Health. Beta-lactam~~- antibacterials,~~ penicillins antibacterial group accounted for 58.79 % ~~of~~ in public sector compared to 27.48 % in ~~the~~ private sector while macrolides, quinolones and other beta-lactam antibacterials accounted for 60.51 % in the private compared to 28.41% in public sector. Consumption of

Formatted: Font: 16 pt, Bold

Formatted: Font: 16 pt, Bold

Formatted: Font: 16 pt, Bold

Formatted: Font: 16 pt, Bold

R~~r~~eserve group antibacterials was negligible, and limited to private sector. ~~Reserve and w~~Watch category antibacterials accounted for 46%, 24% and 54% of the total, public and private sector consumption, respectively.

~~Conclusions: The single most important difference observed between public and private sector was disproportionately higher use of broad spectrum and "Watch category" ABAs in the private sector. Our study has provided the evidence that antibacterial surveillance is possible in resource limited countries and it must be made mandatory.~~

Conclusions

Formatted: Font: 16 pt

A disproportionately higher use of broad spectrum and Watch category antibacterials was observed in the private sector which needs further investigation. This national consumption survey highlights the need and provides the opening for establishment of ABC surveillance in Sri Lanka.

Introduction~~:~~

Formatted: Font: 18 pt

Formatted: Font: 16 pt

Antimicrobial resistance (AMR) is a major ~~global~~ public health challenge [1]. A global survey conducted by the World Health Organization (WHO) in 2014 has shown a high level of resistance to both first line and R~~r~~eserve category antibacerials~~antibacterials~~ ~~was seen~~ for nine pathogenic bacteria responsible for common infections in all WHO regions [~~(1~~2]~~)~~. Infection with such resistant microorganisms result in longer illnesses, increased mortality, prolonged hospital stays and increased overall costs [1,3,4]. Antimicrobial resistance ~~MR~~ affects all areas of health including veterinary and environmental practices and impacts the entire society and its economy [2,5]. ~~Countering AMR needs long term strategies which include strengthening the health systems to ensure more appropriate use of and access to antimicrobial agents.~~

Principally driven by low-and-middle-income countries (LMICs), the global antibacterial consumption (ABC), expressed in defined daily doses (DDD), has increased by 65% from 2000 to 2015 [6]. An increased use of broad-spectrum and last-resort antibacterials was observed in both LMICs and high-income countries (HICs) during this period [6]. If the present policies continue, ABC is expected to increase by 200% in 2030 compared to the consumption in 2015 [6].

~~The development of AMR is an evolutionary process of microbes and is accelerated by the use of antimicrobials. There is a strong association~~The association between ABC and development

of antibacterial resistance (ABR) is well documented ~~AMR and levels of antimicrobial use,~~ and a reduction of inappropriate use of antibacterials ~~microbials~~ could reduce development of resistance [~~(~~7,8]~~2, 3)~~. Countering ABR needs long term strategies which include strengthening the healthcare systems and enacting regulations to ensure appropriate use of and access to antibacterial agents [5]. To enhance access to antibacterials for treatment of commonly occurring infections and their appropriate use, the WHO introduced the Access, Watch, and Reserve (AWaRe) classification of antibacterials as part of Essential Medicines List [9]. With the application of AWaRe classification, the WHO national level target is that 60% of the antibacterials used should be from the Access category by 2023 [9].

~~However, there appears to be an increase in global consumption of antibacterials, more in low- and middle-income countries (4, 5) with an increase in the use of broad-spectrum and last-resort antibacterials (5).~~

The WHO's Global Action Plan (GAP) for Antimicrobial Resistance (AMR) [~~(~~56]~~)~~ calls for member states to put in place national plans to urgently combat AMR. Five strategic objectives have been identified to achieve the goals of the GAP [5]. The fourth objective viz to "Optimize the use of antimicrobial medicines in human and animal health" ~~necessitates provision of~~ needs reliable antimicrobial consumption (AMC) data [5, 10]. Data on AMC are vital to understand AMR, as selection pressure due to use of antimicrobials is a preventable driver for development and spread of AMR [7, 8]. ~~While data on antibiotic use are collected and analysed in many high- and middle-income countries, there is severe paucity of AMC data from lower-income countries (7). Data on AMC are vital to understand AMR, as selection pressure due to use of antimicrobials is a main preventable driver for development and spread of AMR (2, 3). To effectively curtail AMR, surveillance data from AMR must be linked to that of AMC.~~ While data on AMC are collected and analysed in many high- and middle-income countries, there is limited data on AMC from lower-income countries [10]. However, the available data from

LMICs show a greater increase in the use of Watch category antibacterials and a greater reduction in the Access to Watch ratio [6, 11] To effectively curtail AMR, surveillance data from AMR must be linked to that of AMC [12].

Formatted: Font: 12 pt

The WHO methodology for a global programme on surveillance of antimicrobial consumption provides a practical framework to obtain such data in resource limited countries (RLC) [(813]). This involves the collection of “Consumption” and “Use” data and recommends that countries separate “consumption data” from “use data” as the objectives, methods and outcomes for these two categories of data are different. Consumption data” refers to estimates derived from of aggregated data, mainly derived from import, sales or reimbursement databases whereas “use data” refers to estimates derived from patient-level [(813]).

Formatted: Font: 12 pt

Sri Lanka is a lower middle-income country [(914]). Both the public and the private sectors provide allopathic healthcare services in Sri Lanka but the share of care being different for inpatients and outpatients. The public sector provides the bulk of inpatient care [(1095]) while outpatient care is shared. Infections are a leading cause of morbidity and mortality in the public sector health care institutions with zoonotic and other bacterial infections being the 2nd leading cause of death in 2017 [15], with the highest case fatality rates seen from septicaemia and pneumonia. A similar picture was seen in children where pneumonia and other bacterial infections were the 4th and 5th leading causes of death [15]. The absence of morbidity and mortality data from the private sector makes comparisons between the sectors difficult.

Formatted: Font: 12 pt

Formatted: Font: 12 pt

Formatted: Font: 12 pt

The country imports the bulk of its antimicrobials through the State Pharamceuticals Corporation (SPC), which is the State’s procurement arm, and independent private importers. Limited amounts of antimicrobials are manufactured by the State Pharmaceutical ticals Manufacturing Corporation (SPMC) and individual –local manufacturers. The SPC tate

Pharamceuticals Corporation is the sole supplier of antimicrobials to the public sector. It directly imports and also procures and procures antibacterials from local manufaturers. in addition to imports. They animicrobials are distributed to State medical institutions in the public sector by the Medical Supplies Division (MSD) of the Ministry of Health and when antibacterials antibacterials are not available at the MSD, the individual hospitals have the option to procure them antibacterials from the private retail pharmacies as "local purchases". Antibacterials Antimicrobials for the to private healthcare institutions in the private sector are purchased from supplied by both the SPC, and independent private importers and local manufacturers.

Sri Lanka has an established and successful AMR surveillance programme, coordinated by the Sri Lanka College of Microbiologists,. but there is no system in place to obtain aggregated AMC data. The available AMR data shows significant resistance by bacteria causing common infections to 1st line antibacterials [16-18]. However, there is no sytem to obtain aggregated AMC data at present. Available AMC data from Sri Lanka are either limited to pharmaceutical sales data which lacks information of the public sector ABC [6, 19] or only from the state (public) sector and lacks information about consumption in the private sector [20]. The state sector data showed an increase of ABC by 143% (44.4-108.2 million DDDs) with a significant shift towards the use of broad-spectrum antibacterials from 1998 to 2018 [20]. There is, however, no system at present to correlate AMC/ABC data with AMR/ABR patterns in the country.

The Sri Lanka Association of Clinical Pharmacology and Therapeutics (SLACPT), in collaboration the National Focal Point for combating AMR in Sri Lanka, therefore conducted this a national survey surveillance of antibacterial consumption (ABC)for 2017. Though the WHO methodology [13] has defined a core set of antimicrobials namely antibacterials, antibacterials for alimentary tract and nitroimidazole derivatives for protozoal diseases that all

countries should monitor in their surveillance programme, this study has surveyed only the antibacterial consumption (ABC).

This paper outlines the methods adopted, key findings and recommendations for establishment of a national surveillance programme. Our objective was to quantify and describe the national antibacterial consumption in Sri Lanka and compare the consumption between public and private healthcare sectors. This paper presents the methods adopted, discusses the key findings and the problems encountered when conducting the survey. Our findings would be helpful when planning for comprehensive national ABC/AMC surveys or surveillances.

Formatted: Font: 12 pt

Formatted: Font: 14 pt

Formatted: Font: 18 pt

Formatted: Font: 16 pt

Materials and Methods

Background: The methodology of this study was adapted from WHO methodology for a global programme on surveillance of antimicrobial consumption (4).

Study design and data sources

Study design and setting: The methodology of this study was adapted from WHO methodology for a global programme on surveillance of antimicrobial consumption [13]. It was a descriptive cross-sectional study in which aggregate data on antibacterial consumption in 2017 were retrospectively extracted from all available various data sources in 2018.

Antibacterial agents: The WHO methodology recommends to survey antimicrobials including anti-protozoals, anti-fungals, anti-malarials and anti-virals in addition to antibacterial agents (ABAs). However, to start with, we have surveyed only the ABAs listed under antibacterials for systemic use (J01) in the Anatomical Therapeutic Chemical (ATC) classification system [(211]).

Definition of consumption data: The WHO defines consumption data as "estimates derived from aggregated data sources such as import or wholesaler data, or aggregated health insurance data where there is no information available on the patients who are receiving the medicines or why they are being used"(4). National antibacterial consumption (ABC) data provide a proxy estimate of use of these agents in the country.

ABAs suppliers: Local manufacturing of antibacterials ABAs is limited in Sri Lanka and leaving the importers are as tthe major supplier of antibacterials for the countryABAs. State Pharmaceutical Corporation is the sole importer for public sector and Rajya Osusala

Pharmacies (retail pharmacy chain of SPC). It also imports for private market. In addition, there are many importers who cater for private market.

~~**Data sources:**~~ Considering the supply system in Sri Lanka, we approached the Sri Lanka C~~c~~ustoms, Department of Imports and Exports, SPC, State Pharmaceutical Manufacturing Corporation (SPMC), Medical Supplies Division (MSD), private importers and the private manufacturers (list was obtained from the NMRA website, www.nmra.gov.lk accessed on 31st August 2018) for ABC data. As these data sources are expected to provide the data for the entire country, no sampling was done. ~~Formal request letter with evidence of administrative and ethical approval was sent to all these institutions. Investigators personally visited many of these data sources. We accepted both electronic and paper data from the institutions who had extractable ABC data and were willing to provide them.~~ A custom-made MS Excel worksheet was developed based on WHO methodology and our previous experience in ABC surveillance in Colombo district [22]. Data from the MSD were electronically transferred. All other data sources submitted data in paper format and these were manually entered into the Excel worksheet. All the precautions were taken to ensure the accuracy of data entry.

~~**Available data:**~~ The WHO methodology recommends a detailed product-level electronic data to be collected for ABC surveillance programmes. However, for this surveillance, data which had the minimum details, namely name, dosage form, strength and quantity were considered as "complete" and included for analysis.

Measures of antibacterial consumption

The WHO defines consumption data as "estimates derived from aggregated data sources such as import or wholesaler data, or aggregated health insurance data where there is no information available on the patients who are receiving the medicines or why they are being used"(13). National antibacterial consumption (ABC) data provide a proxy estimate of use of these agents

Formatted: Font: 12 pt

Formatted: Font: 12 pt

in the country. **Data entry:** A custom-made MS Excel template was developed based on WHO methodology and our previous experience in ABC surveillance in Colombo district (7). Except the data from the MSD which were electronically transferred, the rest were manually entered as they were in paper format. All the precautions were taken to ensure the accuracy of data entry.

Data analysis: Antibacterials for systemic use (J01, ATC classification level 2) obtained from the data sources were further classified to level 3, 4 and 5. We have presented the consumption data at level 3 (therapeutic or pharmacological sub-group) and level 5 (chemical substance). Quantity of consumption is expressed as Defined Daily Doses (DDDs) using the formula given below. The DDD is defined as “the assumed average maintenance dose per day for a medicine used for its main indication in adults”. Total grams consumed was determined by summing the amounts of active ingredient across the various formulations (different strengths of tablets or capsules, syrup formulations) and pack sizes. The DDD value is assigned by the WHO Collaborating Centre and obtained from their website (http:// www.whocc.no/atc_ddd_index/). Number of DDDs consumed was calculated by dividing the total consumption (in grams) by DDDs (in grams).

Formatted: Comment Text, Line spacing: Double

Field Code Changed

Though the WHO methodology recommends that the variables for consumption estimates should include packages and DDDs, we have used only DDDs as package details were not available for this retrospective survey. surveillance.

Formatted: Comment Text, Space After: 0 pt, Line spacing: Double, Widow/Orphan control, Adjust space between Latin and Asian text, Adjust space between Asian text and numbers

Presentation of data: In addition to presenting the total consumption data obtained from different data sources, we have also compared the public sector data with that of private sector, keeping in mind that the private sector data could be an underestimatean underestimate as it was impossible we have no way to verify the accuracy and completeness of data. We also have compared few key ESAC based quality indicators [23] of ABC as well as the volumes of antibacterials ABAs in the “Access, Watch and Reserve” categories (AWaRe classification) between these two sectors. (12-14)

Formatted: Comment Text, Line spacing: Double

Formatted: Font: 12 pt

Formatted: Font: 12 pt

Formatted: Font: 12 pt

Ethical considerations s and administration approval:

Formatted: Font: 16 pt

~~Ethics Review Committee of Sri Lanka Medical Association exempted this survey from ethics review committee approval (ERC-18/14)~~

Ethics Review Committee of Sri Lanka Medical Association exempted this survey from review (ERC-18/14). A formal letter of request with a copy of ethics review committee's letter of exemption was sent to all the institutions who had the data. Investigators personally visited many of these institutions to explain the aim of survey. Data presented here are from the consenting institutions. Data comprised aggregate data of the amount of antibacterials distributed, imported or manufactured by these institutions and not individual patient or hospital data.

Formatted: Line spacing: Double

Results and discussion~~:~~

Formatted: Font: 18 pt

Formatted: Font: 18 pt

~~**Background:** Of the potential data sources we approached, only SPC, MSD, SPMC (out of 5 local manufacturers contacted) and 12 private importers (out of 78 contacted) provided analyzable data. Both Sri Lanka Customs and Department of Imports and Exports did not have extractable data. We had to discard data provided by a major importer as they lacked the information on strength of the products which is essential to calculate DDD.~~

We analyzed the data from the MSD, SPC, one local manufacturer (SPMC) and 12 private importers. Four local manufacturers did not provide data. Although 78 are registered as private importers with the NMRA, the exact number of Companies actually imported antibacterials in 2017 was not available. The rough estimate from the NMRA records was 40 and 12 of them provided analyzable data. Four out of the 5 leading importers provided analyzable data whilst data provided by one major importer had to be discarded as the Company did not provide the information on strengths of the dosage forms which is essential to calculate DDD. The MSD data is reliable and is the almost complete data for public sector. A very small proportion of antibacterials purchased by individual hospitals as "local purchase" would not have been captured in our data. The SPC provided data separately for public and private sector while SPMC provided cumulative data for both sectors. Data provided by the private importers is their imports for private sector. The SPC data for private sector may well include the data for the Companies who have not provided their imports data to us.

Formatted: Justified

The total volume of antibacterial agents (ABA) (in million DDDs) imported/distributed in 2017 by the respective agencies is shown in Table 1.

Table 1: Total volume of ABAs (in million DDDs) imported/distributed in 2017 by the different agencies

Agency	Volume of ABAs (in million DDDs)
1. Distributed by SPC to private sector	163.04
2. Distributed by MSD to public sector [1]	97.96
3. Distributed by SPMC to both to public and private sector[2]	61.18
4. Imported by private sector	59.30
5. Manufactured by SPMC [2]	56.08
6. Distributed by SPC to MSD [1]	31.43
7. Distributed by SPC to its retail pharmacies (Rajya Osusala)	24.41

1. *MSD distributed to public sector is higher than the SPC distributed to MSD because left over stocks from 2016 could have been distributed by the MSD.*
2. *Manufactured amount is not equal to distributed amount because all the amount manufactured in a particular year would not have been distributed in that particular year*

Despite most of the inpatient care being provided by the public sector health care institutions, the ABC in the private sector (Table 1; data sources 1, 4 and 7) accounted for 246.76 million DDDs compared to 97.96 million DDDs distributed to the public sector by the MSD (data source 2).

The SPC was initially established as a procurement agency for the public sector. However, in 2017 a significant 74.5% of its imports had been for the private sector (163 million DDDs,) while it was only 14.35% to the public sector (31.43 million DDDs)

~~Table 1 shows the total volume of ABAs (in million DDDs) imported/distributed in 2017 by~~

Formatted Table

Formatted: Font: 10 pt, Italic, Font color: Black

Formatted: Normal, No bullets or numbering

Formatted: Font: 10 pt, Italic, Font color: Black

Formatted: List Paragraph, Space After: 0 pt, Numbered + Level: 2 + Numbering Style: 1, 2, 3, … + Start at: 1 + Alignment: Left + Aligned at: 0.5" + Indent at: 0.75", Widow/Orphan control, Adjust space between Latin and Asian text, Adjust space between Asian text and numbers

Formatted: Strikethrough

Formatted: Font: 10 pt, Italic, Font color: Black

Formatted: Font: 10 pt, Italic, Font color: Black, English (United States), Strikethrough

the respective agencies. Consumption of ABAs was very much higher in the private than the public sector: Private sector consumption (Table 1; data sources 1, 4 and 7) accounted for 246.76 million DDDs compared to 97.96 million DDDs distributed to entire public sector by the MSD (data source 2). Additionally, of the annual volume of ABAs distributed by the SPC, the sole agent for the public sector, private sector accounted for 74.5% followed by MSD (14.35%) and Rajya Osusala Pharmacies (11.15%).

Comparison of ABC between public and private sectors

Formatted: Font: 16 pt, Bold

As Sri Lanka has a free public health care system and a fee levying private health care system, we analyzed the data according to the total ABC and in the two sectors. Table 2 compares the consumption of different pharmacological sub-groups of ABAs (3rd level of ATC classification) between public (data source 2) and private sector (data sources 1, 4, 7).

Though the top four groups of ABAs were the same for public and private sector, namely (1) beta-lactam antibacterials, penicillins, (2) other beta-lactam antibacterials, (3) macrolides, and (4) quinolones, major difference in the proportion of volumes consumed was observed between these two sectors; The top four groups of antibacterials consumed in Sri Lanka were beta-lactams, penicillins, other beta-lactams, macrolides and quinolones (Table 2). However, major differences were observed in the proportion of volumes consumed with these categories between the public and private sectors. The beta-lactam antibacterials, penicillins considerably outnumbered (58.57%) the other three groups (13.7%, 7.8%, and 6.8%) in the public sector whereas each of the top four groups accounted for between 15-36% of the ABC in the private sector. Compared to the public sector, the consumption of macrolides, quinolones and other beta lactam antibacterials is disproportionately higher in the private sector.

Formatted: Strikethrough

Table 2: Consumption of different pharmacological sub-groups of antibacterials in the country and public and private sectors

ATC code	ATC level 3 classification of ABAs	ABA consumption volume in DDD per million						DDD per 1000s population
		Public sector (%)		Private sector (%)		Total (%)		
J01A	Tetracycline's	5.94	(6.06)	14.89	0.01	20.83	(6.04)	0.98
J01B	Amphenicols	0.00	(0.00)	0.19	5.90	0.19	(0.06)	0.01
J01C	Beta-lactam antibacterials, penicillins	57.38	(58.57)	67.80	2.62	125.18	(36.31)	5.90
J01D	Other Beta-lactam antibacterials	13.38	(13.66)	42.20	0.10	55.58	(16.12)	2.62
J01E	Sulfonamides and trimethoprim	0.50	(0.51)	1.71	3.22	2.21	(0.64)	0.10
J01F	Macrolide, lincosamide and streptogramins	7.69	(7.78)	60.57	0.01	68.26	(19.80)	3.22
J01G	Aminoglycoside antibacterials	0.14	(0.14)	0.00	2.51	0.14	(0.04)	0.01
J01 M	Quinolone antibacterials	6.66	(6.82)	46.57	0.90	53.23	(15.44)	2.51
J01X	Other antibacterials	6.28	(6.41)	12.84	16.26	19.12	(5.55)	0.90
	**Total**	**97.96**	**100.00**	**246.76**	**0.98**	**344.72**	**100.00**	**16.26**

Reference: Mid-Year Population in 2017 (estimated) – 21,203,000
Mid-year population estimate by district and sex 2014-2019
http://www.statistics.gov.lk/PopHouSat/VitalStatistics/MidYearPopulation/Mid-year%20population%20by%20district.pdf

beta-lactam antibacterials, penicillins considerably outnumbered the other groups in the public sector (8.62:2.01:1.16: 1.00) whereas the proportions were more or less equal in the private sector (1.61:1.00:1.44: 1.10) . Consumption of macrolides, quinolones and other beta lactam ABAs appears to be disproportionately higher in the private compared to public sector.

The consumption of most frequently consumed antibacterials (5th and last level of ATC classification) within these top four groups are shown in table 3. (Detailed data are available in the supplementary Table 1). Substantial differences were observed between public and private sectors in the consumption of individual antibacterials within each of the top four groups. In the private sector co-amoxiclav was the most consumed antibacterial in J01C group and

azithromycin in the J01F group while amoxicillin and erythromycin were the equivalents in the public sector. Interestingly, benzyl penicillin was consumed only in the public sector.

Table 3: Most consumed antibacterials of the top four pharmacological groups in private and public sectors

ATC level 5 classification of ABAs (Code) under each level 3 classification	Name of classes and individual ABAs	Public sector (%)		Private sector (%)	
		DDDs	(%)*	DDDs	(%)*
J01C	Beta-lactam antibacterials, Penicillins				
J01CA04	Amoxicillin	21.91	(22.37)	22.66	(9.18)
J01CE01	Benzyl penicillin	16.95	(17.30)	-	-
J01CF02	Cloxacillin	7.96	(8.13)	2.49	(1.01)
J01CF05	Flucloxacillin	0.32	(0.33)	1.93	(0.78)
J01CR02	Co-Amoxiclav	8.72	(8.90)	39.07	(15.83)
J01D	Other Beta-lactam antibacterials				
J01DB01	Cephalexin	4.57	(4.67)	16.12	(6.53)
J01DC02	Cefuroxime	7.79	(7.95)	20.91	(8.47)
J01DD08	Cefixime	0.05	(0.05)	4.59	(1.86)
J01F	Macrolide, Lincosamide and Streptogramins				
J01FA01	Erythromycin Stearate	3.52	(3.59	6.13	(2.49)
J01FA09	Clarithromycin	2.67	(2.73)	13.58	(5.50)
J01FA10	Azithromycin	1.26	(1.29)	38.74	(15.70)
J01M	Quinolone antibacterials				
J01MA01	Ofloxacin	0.03	(0.03)	0.32	(0.13)
J01MA02	Ciprofloxacin	5.81	(5.93)	34.93	(14.16)
J01MA06	Norfloxacin	0.52	(0.53)	2.76	(1.12)
J01MA12	Levofloxacin	0.22	(0.22)	8.05	(3.26)

* *Percentages are out of total antibacterials consumed in the respective sector. Total is not 100 as only four classes are shown here (see Supplementary Table 1 for full details)*

Quality indicators of ABC for the country as a whole and separately for public and private sectors are given in Table 4.

Table 4: Comparison of few key quality indicators of ABC between public and private sector

Formatted Table

Formatted: Font: Not Bold

Formatted: Font: Not Bold

	Indicator	Public sector	Private sector	Total *
J01CE_%	Consumption of β-lactamase sensitive penicillins (J01CE) expressed as percentage of the total consumption of antibacterials for systemic use (J01)	18.77	0.18	5.46
J01CR_%	Consumption of combination of penicillins, including β -lactamase inhibitor (J01CR) expressed as percentage of the total consumption of antibacterials for systemic use (J01)	8.98	15.83	13.89
J01DD+DE_%	Consumption of third and fourth generation of cephalosporins (J01(DD+DE)) expressed as percentage of the total consumption of antibacterials for systemic use (J01)			
	1st Generation	4.67	6.55	6.01
	2nd Generation	7.95	8.52	8.35
	3rd Generation	0.72	1.98	1.62
	4th Generation	0.00	0.00	0.00
J01MA_%	Consumption of flouroquinolones (J01MA) expressed as percentage of the total consumption of antibacterials for systemic use (J01)	6.71	18.81	15.31
J01_B/N	Ratio of the consumption of broad (J01(CR+DC+DD+(F-FA01))) to the consumption of narrow spectrum penicillins, cephalosporins and macrolides (J01(CE+DB+FA01))	0.81	5.25	2.86

** Indicators are calculated for total data to show how one sector affects the country data*

~~Table 3 compares the consumption of most frequently consumed ABAs (5th and last level of ATC classification) within these top four groups between the public and private sector~~

~~(complete data are available in the supplementary Tables). Substantial differences in the consumption of individual ABAs was observed between public and private sector. For example, consumption of co-amoxiclav, cefuroxime, cefixime, clarithromycin, azithromycin, ciprofloxacin and levofloxacin appears to be disproportionately higher in the private compared to public sector.~~

Antibacterials consumption according to WHO AWaRe Classification

Formatted: Font: 16 pt, Bold

~~Table 4 compares the key indicators of ABC between public and private sector.~~ Fig~~ure~~ 1 shows ~~compares~~ the consumption of antibacterials ~~ABAs~~ in the "Access, Watch and Reserve" ~~"~~categories for the country as a whole and separately for ~~between~~ public and private sector (supplementary Table 2). Of the total antibacterials consumed, 54.19% were from the Access category while 45.57 were from the Watch group with the Access: Watch ratio was 1.18. However, in the public sector this ratio was 3.16 while it was 0.84 in the private sector.

Fig 1: Consumption of antibacterials in Access, Watch and Reserve categories

Formatted: Font: Bold

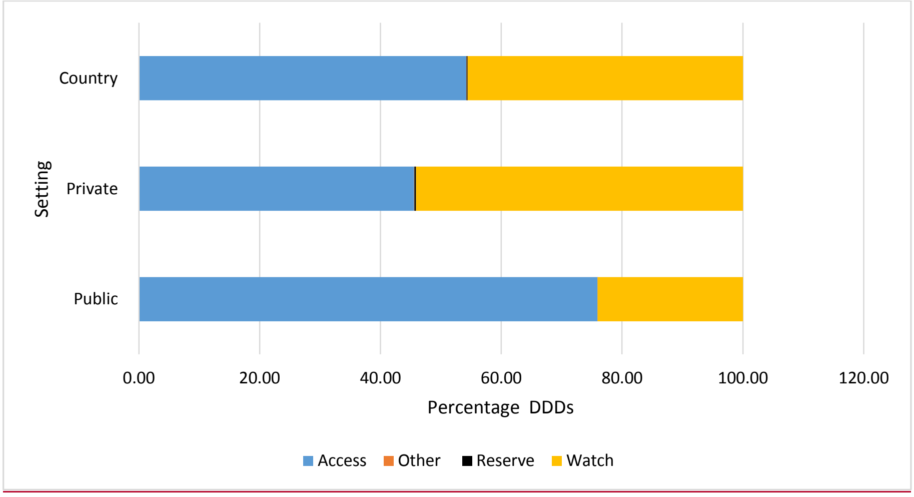

The single most important difference observed between public and private sector (Tables 2,3

and 4, Fig~~ure~~ 1) was disproportionately higher use of broad spectrum antibacterials ~~ABAs~~ in the private sector.

~~Figure 2 shows the top 10 ABAs, oral ABAs and parenteral ABAs consumed in public and private sector. It appears that benzylpenicillin and amoxicillin are scarcely consumed in private as opposed to public sector (Detail tables are available with supplementary data).~~

Formatted: Space After: 12 pt, No widow/orphan control, Don't adjust space between Latin and Asian text, Don't adjust space between Asian text and numbers

We compared the top ten oral and parental antibacterials used between the sectors (Tables 5,6). Both sectors have consumed large amounts of antibacterials in the Watch category and this is more in the private sector. A notable difference was the higher use of parenteral metronidazole and meropenem in private sector compared to that of public sector.

Table 5: Comparison of the top ten oral antibacterials consumed between sectors

Formatted Table

Public sector				Private Sector			
ABM	AwaRe group	DDDs in million	%	ABM	AwaRe group	DDDs in million	%
Amoxicillin	A	21.91	28.96	Co-Amoxiclav	A	38.95	15.89
Cloxacillin	A	7.82	10.33	Azithromycin	W	38.74	15.81
Cefuroxime	W	6.7	8.86	Ciprofloxacin	W	34.91	14.25
Co-Amoxiclav	A	6.2	8.2	Amoxicillin	A	22.66	9.25
Doxycycline	A	5.94	7.85	Cefuroxime	W	20.7	8.45
Ciprofloxacin	W	5.81	7.68	Cephalexin	A	16.12	6.58
Metronidazole	A	4.62	6.11	Doxycycline	A	14.35	5.86
Cephalexin	A	4.57	6.04	Clarithromycin	W	13.49	5.5
Erythromycin	W	3.52	4.66	Metronidazole	A	12.03	4.91
Clarithromycin	W	2.62	3.47	Levofloxacin	W	8.03	3.28

A= Aware W= Watch

Table 6: Comparison of the top ten parental antibacterials consumed between sectors

Formatted Table

Public sector				Private sector			

ABM	AwaRe group	DDDs in million	%	ABM	AwaRe group	DDDs in million	%
Benzyl penicillin	A	16.95	75.98	Flucloxacillin	A	0.35	20.57
Co-amoxiclav	A	2.52	11.29	Ampicillin	A	0.24	14.43
Cefuroxime	W	1.08	4.86	Ceftriaxone	W	0.23	13.53
Ceftriaxone	W	0.48	2.16	Cefuroxime	W	0.21	12.39
Meropenem	W	0.3	1.35	Metronidazole	A	0.14	8.53
Cefotaxime	W	0.14	0.64	Meropenem	W	0.12	7.26
Cloxacillin	A	0.14	0.62	Co-amoxiclav	A	0.12	6.92
Flucloxacillin	A	0.13	0.56	Clarithromycin	W	0.09	5.32
Gentamicin	A	0.11	0.48	Moxifloxacin	W	0.04	2.27
Levofloxacin	W	0.09	0.38	Cefotaxime	W	0.03	1.96

A= Aware W= Watch

Surveillance data on both resistance and consumption are essential to obtain a comprehensive picture of antibacterial resistance. Correlating ABC data with the ABR patterns will help to identify areas that need further action. While national data on ABR patterns are available in Sri Lanka [24,25], to the best of our knowledge this is the first attempt at obtaining national antibacterial consumption data. Previous data from Sri Lanka which have been included in other surveys [6,19,20] have been obtained from either only pharmaceutical sales (6,19) data or only public sector ABC data (20). Adopting WHOs' standard methodology made it possible for us to compare our findings with similar studies done globally and in the region.

We compared Sri Lanka's ABC in 2017 with global surveys on ABC [6, 11] and that of the ESAC-Net countries (26). In 2015, the most commonly consumed antibacterial classes globally were broad-spectrum penicillins (J01CA), cephalosporins (J01D), quinolones (J01M) and macrolides (J01F) [6,11]. This was similar to the top four groups of antibacterials consumed in Sri Lanka. The average total ABC for systemic use (ATC group J01) in the EU/EEA (23.4 DID) [26] was much higher than that of Sri Lanka for 2017 (16.26 DID). A

key finding was the ratio of the consumption of broad spectrum to narrow spectrum penicillins, cephalosporins and macrolides (J01_B/N). This was 2.86 for the country, 0.81 for the public sector but a significant 5.25 for the private sector (Table 4). This ratio is higher than what was seen in the community consumption for ESAC-Net countries i.e. 2.25 [26]. The higher DID and the comparatively lower J01-B/N in ESAC-Net countries could be due to greater access to and better regulation of antimicrobials in these countries.

Direct comparison with other WHO South East Asian Region (SEAR) countries in the region was limited by the lack of studies and the disparity of data sources in different studies. Although South East Asian Region (SEAR) was excluded in the WHO report on surveillance of antibacterial consumption from 2016 – 2018 due to the lack of data, a high level of consumption of cephalosporins and quinolones was found in some countries of the region [2]. A higher consumption of other beta-lactam antibacterials, macrolides and quinolones was seen in the private sector compared to that of the public sector of Sri Lanka. This was somewhat similar to that of the retail private sector in India which also showed an increase in the use of 3rd generation cephalosporins, penicillins with beta-lactamase inhibitors, and of newer classes of antibacterials like carbapenems, lincosamides and glycopeptides [27].

As the WHO recommends that the Watch group should be prioritized as key targets of stewardship programs and monitoring as they have higher resistance potential [9], we analysed the data according to WHO's AWaRe classification (9). Despite a 54.19% overall use of Watch antibacterials in the country, a higher consumption of these antibacterials was seen in the private sector (54.11%) compared to the public sector (24.11%) (Fig 1). This may be an under representation of the consumption of Watch antibacterials in both sectors as local purchase by public sector hospitals and direct purchase from importers by private sector

Formatted: Not Highlight

hospitals and retail pharmacies have not been completely captured in our data. The disparity could be still higher as we do not have complete data from the private sector imports. A similar pattern where more antibacterials in the Watch group are consumed is also seen in several European countries and Japan [10] and in India [16, 28]. The higher consumption of Watch group antibacterials in the private sector in Sri Lanka is a concern as the antibacterial sensitivity patterns are the same for both sectors and practitioners in both sectors are largely the same. It is more alarming as there is an increasing emergence of multi drug resistant bacteria in the country [16-18,29]. It has also been shown that some of the pathogens causing lower respiratory tract infections are resistant to the first line Access antibacterials but show an increased sensitivity to 2nd line (Watch) agents [30]. Infections with these resistant bacteria would add a significant burden to the health budget.

Lack of regulatory oversight, greater accessibility to antibacterials and improved financial status of the patients are possible reasons for greater use of newer and more expensive broad-spectrum antibacterials in the private sector. A major factor that affects antibacterial consumption in the public hospitals in Sri Lanka is the highly limited formulary available to prescribers. The formulary is based on the WHO's and the country's EMLs and the medicines are made available strictly according to it to the public sector institutions. Even though prescribers have the facility to "local purchase" medicines, this does not give them the same availability of medicines as those practicing in the private sector as the patients who seek care from these institutions are largely from lower income segments of the society. However, inappropriate prescriptions of antibacterials are seen in the public sector hospitals too [31,32] and was associated with incorrect physician perception of the need for antibacterials, high patient volume and fear of bacterial super infection [32].

The Government of Sri Lanka has initiated many regulatory mechanisms to curtail inappropriate antibacterial use. All antibacterials are registered under Schedule 11B by the National Medicines Regulatory Authority and are prescription only [33]. Despite such regulations it is still possible to obtain antibacterials from retail pharmacies without a valid prescription [34,35]. Self-medication of antibacterials by patients [35] and the freedom to access any practitioner without an appropriate referral which could lead to duplication and/or inappropriate prescriptions, also contribute to an increase in ABC and AMR in the country. While we do not have complete national data, this paper presents the maximum possible extractable data on ABC in Sri Lanka for the year 2017. As recommended by the WHO for counties which are starting antimicrobial surveillance, we have used procurement/issues data available at the central. This, however, does not reflect what is actually consumed by the end user. The key strength of our study is usage of standardized WHO methodology for reporting ABC in DDD and using ATC classification. The DDD methodology allowed us to use aggregated antibacterial purchase data and made it possible to compare our data with regional and global data. However, the DDD may not reflect the prescribed daily dose (PDD) for individual patients and cannot be used to measure consumption in paediatric wards since the measure is based on adult dosing [36]. It also does not accurately measure antibacterial consumption in cases of renal or hepatic dysfunction, often underestimating the actual antibacterial usage [36].

An important limitation of our study was the inability to capture all national data. This was largely due to incomplete and inadequate record keeping by the Customs and private importers. The inability of the SPMC to provide consumption data based on the sector to which it supplied added to the incomplete national consumption data. For meaningful interpretation of data, the total numbers of DDDs derived as consumption estimates should be

adjusted for the population to which the data apply. Despite these limitations we have adjusted for the population (DID) to compare with similar studies.

Conclusions

Despite limitations our study provides the first national ABC data from Sri Lanka which captures the use in both public and private sectors. Although limited to the health care sector, it highlights the problems a LMIC face when trying to apply globally accepted survey methods to evaluate aspects of ABC. Establishing a central unit to coordinate all activities related to both AMR and AMC, using accepted classification when coding imports, getting the private sector into the programme and creating a central data base which records, analyses and generates statistics routinely are key areas that need attention for better surveillance of antimicrobial use. As antibacterials use is to some extent patient and prescription driven in Sri Lanka, education campaigns targeting both patients and prescribers are needed to change behaviours and prescribing habits. Better implementation of existing regulation is vital to curtail antibacterial misuse.

Table 1: Total volume of ABAs (in million DDDs) imported/distributed in 2017 by the different agencies

Agency	Volume of ABAs (in million DDDs)[8]
1. Distributed by SPC to private sector	163.04
2. Distributed by MSD to public sector [1]	97.96
3. Distributed by SPMC to both to public and private sector	61.18
4. Imported by private sector	59.30
5. Manufactured by SPMC	56.08
6. Distributed by SPC to MSD [1]	31.43
7. Distributed by SPC to its retail pharmacies (Rajya Osusala)	24.41

1. *MSD distributed to public sector is higher than the SPC distributed to MSD because left over stocks from 2016 could have been distributed by the MSD.*
2. *Because of the overlaps between Agencies, we did not total the volume*

ATC code	ATC level 3 classification of ABAs	ABA consumption volume in DDD per million						DDD per 1000s population
		Public sector (%)		Private sector (%)		Total (%)		
J01A	Tetracycline's	5.94	(6.06)	14.89	0.01	20.83	(6.04)	0.98
J01B	Amphenicols	0.00	(0.00)	0.19	5.90	0.19	(0.06)	0.01
J01C	Beta-lactam antibacterials, penicillins	57.38	(58.57)	67.80	2.62	125.18	(36.31)	5.90
J01D	Other Beta-lactam antibacterials	13.38	(13.66)	42.20	0.10	55.58	(16.12)	2.62
J01E	Sulfonamides and trimethoprim	0.50	(0.51)	1.71	3.22	2.21	(0.64)	0.10
J01F	Macrolide, lincosamide and streptogramins	7.69	(7.78)	60.57	0.01	68.26	(19.80)	3.22
J01G	Aminoglycoside antibacterials	0.14	(0.14)	0.00	2.51	0.14	(0.04)	0.01
J01 M	Quinolone antibacterials	6.66	(6.82)	46.57	0.90	53.23	(15.44)	2.51
J01X	Other antibacterials	6.28	(6.41)	12.84	16.26	19.12	(5.55)	0.90
	**Total**	**97.96**	**100.00**	**246.76**	**0.98**	**344.72**	**100.00**	**16.[illegible]**

Formatted: Numbered + Level: 1 + Numbering Style: 1, 2, 3, … + Start at: 1 + Alignment: Left + Aligned at: 0" + Indent at: 0.25"

Formatted Table

Formatted: Justified

Table 2: Consumption of different pharmacological sub-groups of ABAs in private vs. public sector

ATC level 3 classification of ABAs	ABA consumption volume in DDD per million			
	Public sector (%)		Private sector (%)	
Tetracycline's	5.94	(6.06)	14.89	(6.03)
Amphenicols	0.00	(0.00)	0.19	(0.08)
Beta-lactam antibacterials, Penicillins	57.38	(58.57)	67.80	(27.47)
Other Beta-lactam antibacterials	13.38	(13.66)	42.20	(17.10)
Sulfonamides and Trimethoprim	0.50	(0.51)	1.71	(0.69)
Macrolide, Lincosamide and Streptogramins	7.69	(7.78)	60.57	(24.54)
Aminoglycoside antibacterials	0.14	(0.14)	0.00	(0.00)
Quinolone Antibacterials	6.66	(6.82)	46.57	(18.87)
Other antibacterials	6.28	(6.41)	12.84	(5.20)
**Total**	**97.96**	**100.00**	**246.76**	**100.00**

**Table 3:**		DDDs	(%)	DDDs	(%)
J01C	Beta-lactam antibacterials, Penicillins				
J01CA04	Amoxicillin	22.66	(9.18)	21.91	(22.37)
J01CE01	Benzyl penicillin	-	-	16.95	(17.30)
J01CF02	Cloxacillin	2.49	(1.01)	7.96	(8.12)
J01CF05	Flucloxacillin	1.93	(0.78)	0.32	(0.33)
J01CR02	Co-Amoxiclav	39.07	(15.83)	8.72	(8.90)
J01D	Other Beta-lactam antibacterials				
J01DB01	Cephalexin	16.12	(6.53)	4.57	(4.67)
J01DC02	Cefuroxime	20.91	(8.47)	7.79	(7.95)
J01DD08	Cefixime	4.59	(1.86)	0.05	(0.05)
J01F	Macrolide, Lincosamide and Streptogramins				
J01FA01	Erythromycin Stearate	6.13	(2.49)	3.52	(3.60)
J01FA09	Clarithromycin	13.58	(5.50)	2.67	(2.73)
J01FA10	Azithromycin	38.74	(15.70)	1.26	(1.28)
J01M	Quinolone antibacterials				
J01MA01	Ofloxacin	0.32	(0.13)	0.03	(0.04)
J01MA02	Ciprofloxacin	34.93	(14.16)	5.81	(5.93)
J01MA06	Norfloxacin	2.76	(1.12)	0.52	(0.53)
J01MA12	Levofloxacin	8.05	(3.26)	0.22	(0.22)

Formatted Table

Formatted: Font: 10 pt, Italic

Formatted: Justified, Line spacing: single

Table 4: Comparison of few key indicators of ABC between public and private sector

	Indicator	Private sector	Public sector
J01CE_%	Consumption of β-lactamase sensitive penicillins (J01CE) expressed as percentage of the total consumption of antibacterials for systemic use (J01)	0.18	18.77
J01CR_%	Consumption of combination of penicillins, including β -lactamase inhibitor (J01CR) expressed as percentage of the total consumption of antibacterials for systemic use (J01)	15.83	8.98
J01DD+DE_%	Consumption of third and fourth generation of cephalosporins (J01(DD+DE)) expressed as percentage of the total consumption of antibacterials for systemic use (J01)		
	1st Generation	6.55	4.67
	2nd Generation	8.52	7.95
	3rd Generation	1.98	0.72
	4th Generation	0.00	0.00
J01MA_%	Consumption of flouroquinolones (J01MA) expressed as percentage of the total consumption of antibacterials for systemic use (J01)	18.81	6.71
J01_B/N	Ratio of the consumption of broad (J01(CR+DC+DD+(F-FA01))) to the consumption of narrow spectrum penicillins, cephalosporins and macrolides (J01(CE+DB+FA01))	5.25	0.81

~~Figure 1: Consumption of ABAs in Access, Watch and Reserve categories: Comparing total, public and private sector data~~

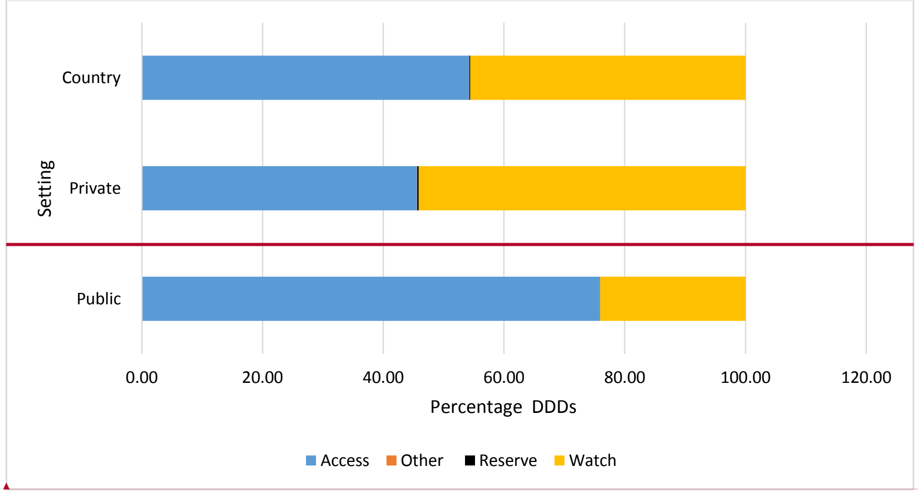

Formatted: Font: Bold

Formatted: Font: Bold

~~Figure 2: Top 10 ABAs, oral ABAs and parenteral ABAs consumed in public and private sector.~~

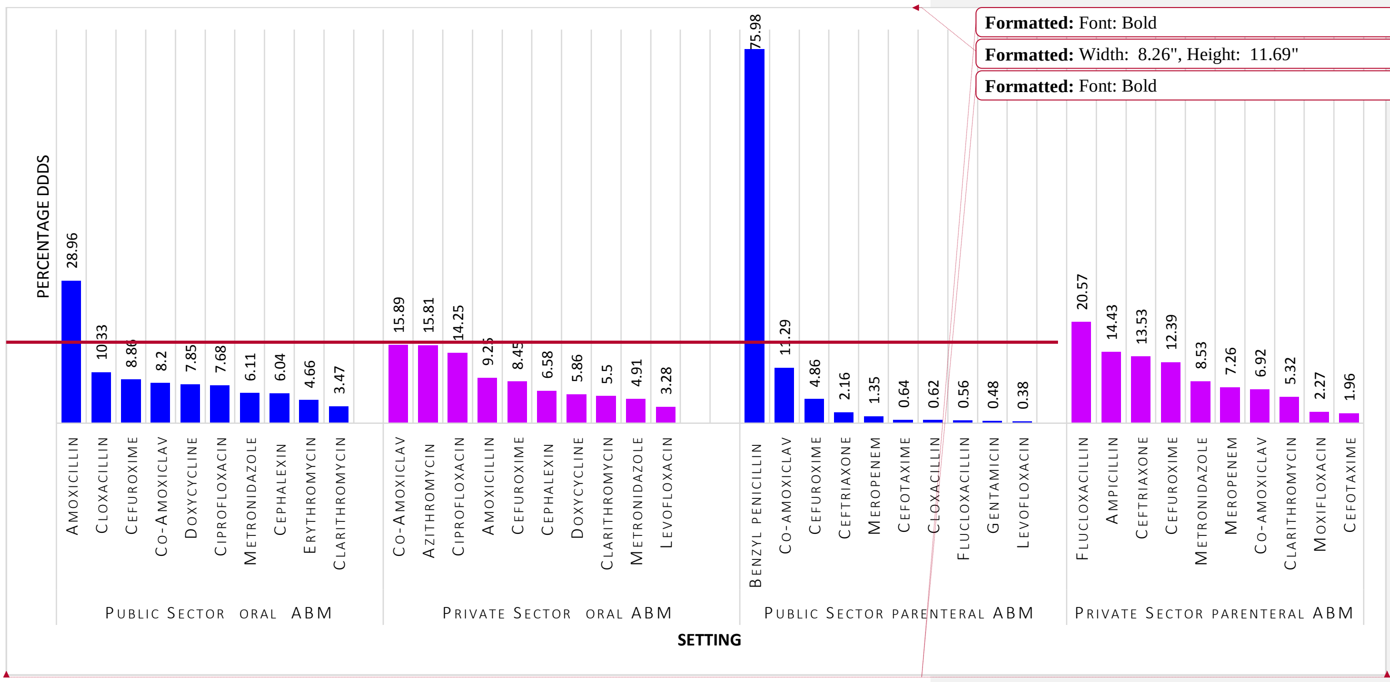

ABM- Antibacterial medicine

Formatted: Font: Bold

Formatted: Width: 8.26", Height: 11.69"

Formatted: Font: Bold

Formatted: Font: Bold

Formatted: Font: Bold

Discussion

Surveillance data on both resistance and consumption of antibacterials is essential to obtain a through comprehensive picture of antibiotic resistance and allows for identification of areas that need further action. While national data on antibiotic resistance patterns are available in Sri Lanka, this is the first attempt at obtaining antibiotic consumption data of the country. There is also very little data on ABC from the SEARO region (7). While we do not have complete national data, this paper presents the maximum possible extractable data on ABC in Sri Lanka for the year 2017. As recommended by the WHO for counties which are starting antimicrobial surveillance, we have used procurement/issues data available at the central level which, however, does not reflect what is actually consumed by the end user.

Formatted: Font: Bold

The total country data shows an almost similar consumption of both Access (54.23%) and Watch (45.6%) antibacterials, but a higher consumption of Watch antibacterials is seen in the private sector (54.11%) compared to the public sector (24.11%). This may also be an under representation of the consumption of Watch antibacterials in both sectors as local purchase by public sector hospitals and direct purchase from importers by private sector hospitals and retail pharmacies have not been completely captured. The disparity could be still higher as we do not have complete data from the private sector. The WHO recommends that the Watch group should be prioritized as key targets of stewardship programs and monitoring as they have higher resistance potential (15).

Despite the public sector providing the bulk of inpatient care, the ABC in the private sector was approximately three times higher than that the public sector. The SPC's supply to the private sector is almost 6 times that of to the public sector. This is despite

SPC being only one of the suppliers to the private sector while being the sole supplier to the MSD which supplies the entire public sector.

Analysing the data according to WHO's EML and AWaRe classification (13) enabled us to understand the use of 2nd line agents. The higher consumption of Watch group antibacterials in the private sectors is also of concern as the antibiotic sensitivity patterns are the same for both sectors and practitioners in both sectors are largely the same. Lack of oversight and greater financial flexibility are possible reasons for greater use of more expensive broad-spectrum antibacterials in the private sector.

Formatted: Font: (Default) Times New Roman, Bold

Formatted: Justified, Line spacing: Double

Direct comparison with other WHO regions was limited by the disparity of data sources between different regions and countries. Limited data from WHO SEARO countries show a high level of consumption of cephalosporins and quinolones in some of the countries, and a very high level of consumption of third generation cephalosporins in all States of India(7). This is similar to what was seen in Sri Lanka and an AMC pattern similar to our private sector where more antibacterials in the Watch group are consumed is also seen in several European countries and Japan (7) and in India (16, 17).

Formatted: Font: Bold

Formatted: Font: (Default) Times New Roman, Bold

Formatted: Justified, Line spacing: Double

Formatted: Font: Bold

Formatted: Adjust space between Latin and Asian text, Adjust space between Asian text and numbers

The key strength of our study is usage of standardized WHO methodology for reporting ABC in DDD and using ATC classification. These allowed for some comparison with regional and global data where available.

Formatted: Justified, Line spacing: Double

An important limitation of our study is the inability to capture all national data. This was largely due to incomplete and inadequate record keeping by the Customs and private importers. The inability of the SPMC to provide consumption data based on the sector to

which it supplied added to the incomplete national consumption data. For meaningful interpretation of data, the total numbers of DDDs derived as consumption estimates should be adjusted for the population to which the data apply. Since we couldn't obtain national estimate of consumption, we have not adjusted for the population. We limited the surveillance to ABAs and did not include other antimicrobials. However, recommendations from the ABAs surveillance will be applicable to AMC surveillance as well.

Formatted: Font: (Default) Times New Roman, Bold

Formatted: Justified, Line spacing: Double

Conclusions

Formatted: Font: Bold

Formatted: Justified

Our study, although limited to the health sector, highlights the problems a LMIC face when trying to apply globally accepted surveillance methods to determine ABC. It also highlights the need for better regulation of ABC to reduce emergence of resistance strains as we have reported disproportionally higher use of broad spectrum and "Watch category ABAs" in the private sector. Establishing a central unit to coordinate all activities related to both AMR and AMC, using accepted classification when coding imports, getting the private sector into the programme, creating a central data base which records, analyses and generates statistics routinely are some of the key recommendations for LMICs trying to establish routine antimicrobial surveillance programmes.

Acknowledgements: We acknowledge the Sri Lanka Association of Clinical Pharmacology and Therapeutics (SLACPT) for funding the data entry personnel Mr P.A.A.S.P. Kumara for assisting in data entry, Dr Malitha Rubesinghe for coordinating approvals and all the officials and pharmacists who facilitated data extraction.

Formatted: Space Before: 0 pt, After: 0 pt

The authors declare that they have no competing interests

References

1. Laxminarayan R, Duse A, Wattal C, Zaidi AKM, Wertheim HFL, Sumpradit N, et al. Antibacterial resistance-the need for global solutions. The Lancet Infectious Diseases, 2013; 13(12), 1057-1098. doi: 10.1016/S1473-3099(13)70318-9
2. World Health Organization. Antimicrobial resistance: Global report on Surveillance 2014.
3. Centers for Disease Control and Prevention. Antibacterial Resistance Threats in the United States, 2019. Atlanta, GA: U.S. Department of Health and Human Services, CDC;

Formatted: Font: Bold

Formatted: Font: Font color: Black

Formatted: List Paragraph, Numbered + Level: 1 + Numbering Style: 1, 2, 3, … + Start at: 1 + Alignment: Left + Aligned at: 0" + Indent at: 0.25"

Formatted: Font: Font color: Black

Formatted: Font color: Custom Color(RGB(32,31,30)), Border: : (No border)

Formatted: Font: Font color: Custom Color(RGB(32,31,30))

Formatted: Font: Font color: Custom Color(RGB(32,31,30))

Formatted: Font:

Formatted: Font:

2019. Available at https://www.cdc.gov/drugresistance/pdf/threats-report/2019-ar-threats-report-508.pdf. Accessed 18th May 2021

4. Ventola CL. “The antibacterial resistance crisis: part 1: causes and threats.” P & T : a peer-reviewed journal for formulary management vol. 40,4 (2015): 277-83.
5. World Health Organization. Global Action Plan on Antimicrobial Resistance: World Health Organization; 2015.
6. Klein EY, Van Boeckel TP, Martinez EM, Pant S, Gandra S, Levin SA, et al. Global increase and geographic convergence in antibacterial consumption between 2000 and 2015. Proceedings of the National Academy of Sciences. 2018;115(15):E3463-E70. doi:10.1073/pnas.1717295115
7. Goossens H, Ferech M, Vander Stichele R, Elseviers M, Group EP. Outpatient antibacterial use in Europe and association with resistance: a cross-national database study. The Lancet. 2005; 365(9459):579-87. doi:10.1016/ S0140-6736(05)17907-0
8. Costelloe C, Metcalfe C, Lovering A, Mant D, Hay AD. Effect of antibacterial prescribing in primary care on antimicrobial resistance in individual patients: systematic review and meta-analysis. Bmj. 2010;340. doi:10.1136/bmj.c2096
9. World Health Organization model list of essential medicines, 21st List, 2019. Geneva: World Health Organization, 2019.
10. World Health Organization. WHO Report on surveillance of antibacterial consumption: 2016-2018 early implementation. 2018.
11. Versporten A, Zarb P, Caniaux I, Gros MF, Drapier N, Miller M, et al. Global-PPS network. Antimicrobial consumption and resistance in adult hospital inpatients in 53 countries: results of an internet-based global point prevalence survey. Lancet Glob Health. 2018; 6(6):e619-e629. doi: 10.1016/S2214-109X(18)30186-4.
12. Surveillance and monitoring for antimicrobial use and resistance, IAGC discussion paper, June 2018. Available at https://www.who.int/antimicrobial-resistance/interagency-coordination-group/IACG_Surveillance_and_Monitoring_for_AMU_and_AMR_110618.pdf Accessed on 18th May 2021

Formatted: Hyperlink, Font: No underline, Font color: Auto, Border: : (No border)

Field Code Changed

Formatted: Font color: Custom Color(RGB(32,31,30)), Border: : (No border)

Formatted: Font:

Formatted: Font:

Formatted: Font color: Custom Color(RGB(32,31,30)), Border: : (No border)

Formatted: Font: Font color: Custom Color(RGB(32,31,30))

Formatted: Font: Font color: Custom Color(RGB(32,31,30))

Formatted: Font: Font color: Custom Color(RGB(32,31,30))

Formatted: Font: Times New Roman, 12 pt

Formatted: Font color: Custom Color(RGB(32,31,30)), English (United States), Border: : (No border)

Formatted: Font: Font color: Custom Color(RGB(32,31,30))

Formatted: Font: Font color: Custom Color(RGB(32,31,30))

Formatted: Font: Times New Roman, 12 pt

Formatted: Font color: Custom Color(RGB(32,31,30)), English (United States), Border: : (No border)

Formatted: Font: Font color: Custom Color(RGB(32,31,30))

Formatted: Font: Font color: Custom Color(RGB(32,31,30))

Formatted: Font:

Formatted: Font: Times New Roman, Font color: Custom Color(RGB(32,31,30)), English (United States), Border: : (No border)

Formatted: Font: Font color: Custom Color(RGB(32,31,30))

Formatted: English (United States), Border: : (No border)

Formatted: Font: Font color: Custom Color(RGB(32,31,30))

Formatted: Font: Font color: Custom Color(RGB(32,31,30))

Formatted: Border: : (No border)

Formatted: Font:

Formatted: Font color: Custom Color(RGB(32,31,30)), Border: : (No border)

Formatted: Font: Font color: Custom Color(RGB(32,31,30))

Formatted: Font: 12 pt

Formatted: Font color: Custom Color(RGB(32,31,30))

13. World Health Organization. WHO methodology for a global programme on surveillance of antimicrobial consumption. World Health Organization: 2017.
14. The world bank in Sri Lanka -Overview. Available from: https://www.worldbank.org/en/country/srilanka/overview. Accessed 18th May 2021
15. Annual Health Bulletin 2018, Ministry of Health, Sri Lanka Available at: http://www.health.gov.lk/moh_final/english/public/elfinder/files/publications/AHB/2020/AHB_2017.pdf. Accessed 18th May 2021
16. Jayatilleke S, Patabedige G, Dassanayake M. Analysis of urine culture isolates from seven laboratories of Sri Lanka: National Laboratory Based Surveillance of Sri Lanka College of Microbiologists in 2014. Sri Lankan Journal of Infectious Diseases. 2016;6(1):17–24. doi: http://doi.org/10.4038/sljid.v6i1.8105
17. Perera V, Assellage P and De Silva P. Profile of extended spectrum βlactamase (ESBL) producing enterobacteria isolated from clinical specimens in a private teaching hospital in Sri Lanka. The Bulletin of the Sri Lanka College of Microbiologists. 2014; 12(1):19-20
18. Fernando MMPSC, Luke WANV, Miththinda JKND, Wickramsingha RDSS, Sebastiampillai BS, Gunathilake MPML et al. Extended spectrum beta lactamase producing organisms causing urinary tract infections in Sri Lanka and their antibacterial susceptibility pattern –A hospital based cross sectional study. BMC Infect Dis. 2017:17, 13.doi:10.1186/s12879-017-2250-y
19. Klein EY, Milkowska-Shibata M, Tseng KK, Sharland M, Gandra S, Pulcini C, Laxminarayan R. Assessment of WHO antibacterial consumption and access targets in 76 countries, 2000–15: an analysis of pharmaceutical sales data. The Lancet Infectious Diseases, 2021;21(1), 107-115. doi:10.1016/S1473-3099(20)30332-7
20. Fernandopulle BMR, Yasarathna KWGKP, Gunasekara ADM, Wijesinghe KGCD, Beneragama BVSH, Panapitiya L et al. Antibacterial Consumption in the State Sector of Sri Lanka Over 25 Years: 1994 - 2018 Using the GAP Methodology. The Pharmaceutical Journal of Sri Lanka 2020 10(1): 54-63. doi: 10.4038/pjsl.v10i1.57

Formatted: Font: Font color: Custom Color(RGB(32,31,30))

Field Code Changed

Formatted: Hyperlink, Font: No underline, Font color: Auto, Border: : (No border)

Formatted: Font: Font color: Custom Color(RGB(32,31,30))

Field Code Changed

Formatted: Hyperlink, Font: No underline, Font color: Auto, Border: : (No border)

Formatted: Font: Font color: Black

Field Code Changed

Formatted: Hyperlink, Font: English (United States), Border: : (No border)

Formatted: Font color: Custom Color(RGB(32,31,30)), English (United States), Border: : (No border)

Formatted: Font: (Default) inherit, Font color: Custom Color(RGB(32,31,30))

Formatted: List Paragraph, Numbered + Level: 1 + Numbering Style: 1, 2, 3, … + Start at: 1 + Alignment: Left + Aligned at: 0" + Indent at: 0.25"

Formatted: Font: Times New Roman, Border: : (No border)

Formatted: Font: Font color: Gray-80%

Formatted: List Paragraph, Numbered + Level: 1 + Numbering Style: 1, 2, 3, … + Start at: 1 + Alignment: Left + Aligned at: 0" + Indent at: 0.25"

Formatted: Font: Font color: Gray-80%

Formatted: Font color: Custom Color(RGB(32,31,30)), Border: : (No border)

Formatted: Font: Font color: Black

Formatted: Font: Font color: Black

Formatted: Font color: Custom Color(RGB(32,31,30)), Border: : (No border)

Formatted: Font: Font color: Custom Color(RGB(32,31,30))

Formatted: Font: Font color: Custom Color(RGB(32,31,30))

Formatted: Font: Font color: Black

Formatted: Font: 11 pt, Font color: Custom Color(RGB(32,31,30)), English (United States), Border: : (No border)

21. World Health organization, WHO Collaborating centre for drug statistics methodology. Available: https://www.whocc.no/atc_ddd_index/.

22. Senadheera GPSG, Sri Ranganathan S, Fernando GH, Fernandopulle BMR. Antibacterial Medicine Consumption in Private and State Sector Outpatient Settings in Colombo District, Sri Lanka. Pharmaceutical Journal of Sri Lanka. 2019;9(1):28–39. DOI: http://doi.org/10.4038/pjsl.v9i1.49

23. Coenen S, Ferech M, Haaijer-Ruskamp FM, Butler CC, Vander Stichele RH, Verheij TJ et al.; ESAC Project Group. European Surveillance of Antimicrobial Consumption (ESAC): quality indicators for outpatient antibacterial use in Europe. Qual Saf Health Care. 2007 Dec;16(6):440-5. doi: 10.1136/qshc.2006.021121.

24. JayatillekeS, Patabedige G, Dassanayake M. Analysis of urine culture isolates from seven laboratories of Sri Lanka: National Laboratory Based Surveillance of Sri Lanka College of Microbiologists in 2014. Sri Lankan Journal of Infectious Diseases, 2016;6(1):17–24. doi: 10.4038/sljid.v6i1.8105

25. ARSP Working Group, The Sri Lanka College of Microbiologists. A multi centre laboratory study of Gram negative bacterial blood stream infections in Sri Lanka. Ceylon Med J. 2013 Jun;58(2):56-61. doi: 10.4038/cmj.v58i2.5680

26. European Centre for Disease Prevention and Control. Antimicrobial consumption. In: ECDC. Annual epidemiological report 2017. Stockholm: ECDC; 2018.

27. Farooqui HH, Selvaraj S, Mehta A, Heymann DL. Community level antibacterial utilization in India and its comparison vis-à-vis European countries: Evidence from pharmaceutical sales data. PLoS ONE; 2018:13(10): e0204805. doi:10.1371/journal.pone.0204805

28. McGettigan P, Roderick P, Kadam A, Pollock AM. Access, Watch, and Reserve antibacterials in India: challenges for WHO stewardship. Lancet Glob Health. 2017;5(11):e1075-e1076. doi: 10.1016/S2214-109X(17)30365-0.

29. Kumuduni WGM, Wijesooriya LI, Namalie KD, Sunil-Chandra NP, Wijayasinghe YS. Epidemiology of multidrug-resistant Enterobacteriaceae in Sri Lanka: First evidence of

Formatted: Font: (Default) TimesNewRomanPSMT, Font color: Custom Color(RGB(32,31,30))

Formatted: Font: Font color: Custom Color(RGB(32,31,30))

Formatted: Font: (Default) TimesNewRomanPSMT, Font color: Custom Color(RGB(32,31,30))

Formatted: Font: Times New Roman, 11 pt, Border: : (No border)

Formatted: Font: 11 pt, Font color: Custom Color(RGB(32,31,30)), Border: : (No border)

Formatted: Font:

Formatted: Font:

Formatted: Font: 11 pt, Font color: Custom Color(RGB(32,31,30)), Border: : (No border)

Formatted: Font: Font color: Black

Formatted: Font: 11 pt, Font color: Custom Color(RGB(32,31,30)), English (United States), Border: : (No border)

Formatted: Font:

Formatted: Font: 11 pt, Font color: Custom Color(RGB(32,31,30)), Border: : (No border)

Formatted: Font: (Default) inherit

Formatted: Font: Times New Roman, 11 pt, Font color: Custom Color(RGB(32,31,30)), English (United States), Border: : (No border)

Formatted: Font:

Formatted: Font:

Formatted: Font: 11 pt, Font color: Custom Color(RGB(32,31,30)), Border: : (No border)

Formatted: Font: Font color: Custom Color(RGB(32,31,30))

Formatted: Font:

Formatted: Font:

Formatted: Font: 11 pt, Font color: Custom Color(RGB(32,31,30)), Border: : (No border)

Formatted: Font: Font color: Custom Color(RGB(19,20,19))

blaKPC harboring Klebsiella pneumoniae. Journal of Infection and Public Health; 2020:13(9), 1330-1335. doi: 10.1016/j.jiph.2020.04.010.

30. Amarasinghe N, Athavan M, Jayamanne D, Rajapakshe Y, Sadikeen A, Gunasekera K et al. Bacterial profile and antibacterial susceptibility pattern of adult lower respiratory tract infections in Colombo, Sri Lanka. Journal of Health and Social Sciences 2018; 3,1:27

31. Zhang HL, Bodinayake C, Wijayaratne GB, Jayatissa P, Piyasiri B, Kurukulasooriya R et al. Point-prevalence survey of outpatient antibacterial prescription at a tertiary medical center in Sri Lanka: opportunities to improve prescribing practices for respiratory illnesses. BMC Infect Dis; 2021:21, 97). Doi:10.1186/s12879-021-05804-6

32. Tillekeratne LG, Bodinayake CK, Dabrera T, Nagahawatte A, Kodikara Arachchi W, Sooriyaarachchi A, et al. Antibacterial overuse for acute respiratory tract infections in Sri Lanka: a qualitative study of outpatients and their physicians. BMC Fam Pract; 2017: 18, 37. doi:10.1186/s12875-017-0619-z

33. National Medicines Regulatory Authority Act, No. 5 of 2015. Available at https://nmra.gov.lk/images/PDF/gazzet/PG--4687--E.pdf. Accessed on 18th May 2021

34. Zawahira S, Lekamwasam S, Aslania P. Antibacterial dispensing practice in Sri Lankan community pharmacies: Asimulated client study. Research in Social and Administrative Pharmacy; 2019: 15(5), 584-590

35. Senadheera GPSG, Sri Ranganathan S, Fernando GH, Fernandopulle BMR. Practice of self-medication with antibacterials in the Colombo district, Sri Lanka. Ceylon Medical Journal 2017; 62: 70-72. doi:10.4038/cmj.v62i1.8439

36. Gravat LAH, Pakyz AL. Challenges in Measuring Antibacterial Consumption. Curr Infect Dis Rep. 2013; 15:559–563. doi: 10.1007/s11908-013-0374-9

~~References~~

Formatted: Font: 11 pt, Font color: Custom Color(RGB(32,31,30)), English (United States), Border: : (No border)

Formatted: Font: Font color: Black

Formatted: Font: Font color: Black

Formatted: Font:

Formatted: Font: 11 pt, English (United States), Border: : (No border)

Formatted: Font:

Formatted: Font: Font color: Custom Color(RGB(19,20,19))

Formatted: Font: 11 pt

Formatted: Font: 12 pt

Formatted: List Paragraph, Indent: Left: 0.25"

Formatted: Font: 12 pt

Formatted: Font:

Formatted: Font: Font color: Gray-80%

Formatted: List Paragraph, Numbered + Level: 1 + Numbering Style: 1, 2, 3, … + Start at: 1 + Alignment: Left + Aligned at: 0" + Indent at: 0.25"

Formatted: Font:

Formatted: Font: Font color: Gray-80%

Formatted: Font: Font color: Gray-80%

Formatted: Font:

Formatted: Font: Font color: Custom Color(RGB(32,31,30))

Formatted: Font: (Default) TimesNewRoman,Bold, Font color: Custom Color(RGB(32,31,30))

Formatted: Font: Font color: Custom Color(RGB(32,31,30))

Formatted: Font: (Default) TimesNewRoman,Bold, Font color: Custom Color(RGB(32,31,30))

Formatted: Font: Times New Roman, Font color: Black, Border: : (No border)

Formatted: Font: Font color: Custom Color(RGB(32,31,30))

Formatted: Font: Font color: Custom Color(RGB(32,31,30))

Formatted: Font color: Black, English (United States), Border: : (No border)

Formatted: Font: Font color: Custom Color(RGB(32,31,30))

Formatted: Font:

Formatted: Font:

Formatted: Font color: Black, English (United States), Border: : (No border)

Formatted: Font:

Formatted: List Paragraph, Numbered + Level: 1 + Numbering Style: 1, 2, 3, … + Start at: 1 + Alignment: Left + Aligned at: 0" + Indent at: 0.25", Pattern: Clear (White)

Formatted: Font:

1. World Health Organization. Antimicrobial resistance Global report on Surveillance 2014.

2. Goossens H, Ferech M, Vander Stichele R, Elseviers M, Group EP. Outpatient antibiotic use in Europe and association with resistance: a cross-national database study. The Lancet. 2005; 365(9459):579-87.

doi:10.1016/ S0140-6736(05)17907-0

3. Costelloe C, Metcalfe C, Lovering A, Mant D, Hay AD. Effect of antibiotic prescribing in primary care on antimicrobial resistance in individual patients: systematic review and meta-analysis. Bmj. 2010;340.

doi:10.1136/bmj.c2096

4. Van Boeckel TP, Gandra S, Ashok A, Caudron Q, Grenfell BT, Levin SA, et al. Global antibiotic consumption 2000 to 2010: an analysis of national pharmaceutical sales data. The Lancet infectious diseases. 2014;14(8):742-50.

doi:10.1016/S1473-3099(14)70780-7

5. Klein EY, Van Boeckel TP, Martinez EM, Pant S, Gandra S, Levin SA, et al. Global increase and geographic convergence in antibiotic consumption between 2000 and 2015. Proceedings of the National Academy of Sciences. 2018;115(15):E3463-E70.

doi:10.1073/pnas.1717295115

6. World Health Organization. Global Action Plan on Antimicrobial Resistance.: World Health Organization; 2015.

7. World Health Organization. WHO Report on surveillance of antibiotic consumption: 2016-2018 early implementation. 2018.

8. World Health Organization. WHO methodology for a global programme on surveillance of antimicrobial consumption. World Health Organization: 2017.

9. The world bank in Sri Lanka - "Overview ". Available from: https://www.worldbank.org/en/country/srilanka/overview.

Formatted: Normal, No bullets or numbering

Formatted: Normal, Indent: Left: 0"

Formatted: Normal, No bullets or numbering

Formatted: Normal, Indent: Left: 0"

Formatted: Normal, No bullets or numbering

Formatted: Normal, Indent: Left: 0"

Formatted: Normal, No bullets or numbering

Formatted: Normal, Indent: Left: 0"

Formatted: Normal, No bullets or numbering

10. World Health Organization. Antimicrobial resistance: global report on surveillance: World Health Organization; 2014.

11. World Health organization, WHO Collaborating centre for drug statistics methodology. Available: https://www.whocc.no/atc_ddd_index/.

12. Coenen S, Ferech M, Haaijer-Ruskamp FM, Butler CC, Vander Stichele RH, Verheij TJ, et al. European Surveillance of Antimicrobial Consumption (ESAC): quality indicators for outpatient antibiotic use in Europe. BMJ Quality & Safety. 2007;16(6):440-5.

DOI: 10.1136/qshc.2006.021121

13. Sharland M, Pulcini C, Harbarth S, Zeng M, Gandra S, Mathur S, et al. Classifying antibiotics in the WHO Essential Medicines List for optimal use—be AWaRe. The Lancet Infectious Diseases. 2018;18(1):18-20.

DOI: 10.1016/S1473-3099(17)30724-7

14. World Heath organization Organization Regional office for Europe. Antimicrobial Medicines Consumption (AMC) Network. AMC data 2011–2014. World Health Organization Regional office for Europe; 2017

https://www.euro.who.int/en/publications/abstracts/antimicrobial-medicines-consumption-amc-network.-amc-data-20112014-2017

15. World Health Organization. Organization. WHO releases the 2019 AWaRe Classification Antibiotics.

https://www.who.int/medicines/news/2019/WHO_releases2019AWaRe_classification_antibiotics/en/

16. Kotwani A, Ewen M, Dey D, Iyer S, Lakshmi P, Patel A, et al. Prices & availability of common medicines at six sites in India using a standard methodology. Indian journal of medical research. 2007;125(5):645.

PMID: 17642500.

Formatted: Normal, Indent: Left: 0"

Formatted: Normal, No bullets or numbering

Formatted: Normal, Indent: Left: 0"

Formatted: Normal, No bullets or numbering

Formatted: Normal, Indent: Left: 0"

Formatted: Normal, No bullets or numbering

Formatted: Normal, Indent: Left: 0", Line spacing: 1.5 lines

Formatted: Normal, Indent: Left: 0"

Formatted: Normal, No bullets or numbering

Formatted: Normal, Indent: Left: 0"

17. Farooqui HH, Selvaraj S, Mehta A, Heymann DL. Community level antibiotic utilization in India and its comparison vis-à-vis European countries: Evidence from pharmaceutical sales data. PloS one. 2018;13(10):e0204805.

doi: 10.1016/S1473-3099(17)30724-7.

Formatted: Normal, No bullets or numbering

Formatted: Normal, Indent: Left: 0"

Formatted: Line spacing: 1.5 lines

PONE-D-21-11873
National surveillance of antibacterial consumption in Sri Lanka

Amended title: A National survey of antibacterial consumption in Sri Lanka

Response to Reviewers

COMMENTS	RESPONSE FROM AUTHORS
**ACADEMIC EDITOR**	
Please ensure that your manuscript meets PLOS ONE's style requirements, including those for file naming.<br>The PLOS ONE style templates can be found at<br><br>https://journals.plos.org/plosone/s/file?id=wjVg/PLOSOne_formatting_sample_main_body.pdf and<br><br>https://journals.plos.org/plosone/s/file?id=ba62/PLOSOne_formatting_sample_title_authors_affiliations.pdf	Thank you very much<br><br>We apologize for not following the journal style<br><br>We have now revised the whole manuscript based on the journal style guide for title page, main body, tables, figure and references
Please provide additional details regarding participant consent. In the ethics statement in the Methods and online submission information, please ensure that you have specified (1) whether consent was informed and (2) what type you obtained (for instance, written or verbal, and if verbal, how it was documented and witnessed). If your study included minors, state whether you obtained consent from parents or guardians. If the need for consent was waived by the ethics committee, please include this information.<br><br>If you are reporting a retrospective study of medical records or archived samples, please ensure that you have discussed whether all data were fully anonymized before you accessed them and/or whether the IRB or ethics committee waived the requirement for informed consent. If patients provided informed written consent to have data from their medical records used in research, please include this information.	Thank you<br><br>This is a retrospective study of medicine supplies/imports data<br><br>Ethics Review Committee of Sri Lanka Medical Association exempted this survey from review (ERC-18/14). A formal letter of request with a copy of ethics review committee’s letter of exemption was sent to all the institutions who had the data. Investigators personally visited many of these institutions to explain the aim of survey. Data presented here are from the consenting institutions. Data comprised aggregate data of the amount of antibacterials distributed, imported or manufactured by these institutions and not individual patient or hospital data.<br><br>We have stated this under ethical considerations in the methods section

We note that you have indicated that data from this study are available upon request. PLOS only allows data to be available upon request if there are legal or ethical restrictions on sharing data publicly. For information on unacceptable data access restrictions, please see http://journals.plos.org/plosone/s/data-availability#loc-unacceptable-data-access-restrictions	Please see below (cover letter comment)
**Cover letter**<br>a) If there are ethical or legal restrictions on sharing a de-identified data set, please explain them in detail (e.g., data contain potentially identifying or sensitive patient information) and who has imposed them (e.g., an ethics committee). Please also provide contact information for a data access committee, ethics committee, or other institutional body to which data requests may be sent.<br>b) If there are no restrictions, please upload the minimal anonymized data set necessary to replicate your study findings as either Supporting Information files or to a stable, public repository and provide us with the relevant URLs, DOIs, or accession numbers. Please see http://www.bmj.com/content/340/bmj.c181.long for guidelines on how to de-identify and prepare clinical data for publication. For a list of acceptable repositories, please see http://journals.plos.org/plosone/s/data-availability#loc-recommended-repositories.	The data underlying this study belong to many third parties, Ministry of Health, State Pharmaceutical Corporation, State Pharmaceutical Manufacturing Corporation and private Pharmaceutical Companies. Raw data used to analyse antibiotic consumption in this manuscript is either state owned or private company/industry owned. Data were given to us under the agreement that the raw data will not be shared. Hence, we are unable to provide raw data in public domain. We confirm that we did not have any special access to the data that other researchers would not have. They have to approach these institutions with the letter from ethics review committee and administrative clearance
**REVIEWER 1**	
The manuscript addresses a timely topic which is very important for all the developing countries. It also stresses the possibility of antibiotic abuse (especially second line antibiotics) within the private sector which can be controlled with	Thank you very much. The typo has been deleted.

appropriate measures as discussed in the article. It is well written except for a few changes as per my comments. Statistically, they have only used percentages which is descriptive enough given the content of the article. Please delete the words that were highlighted in the Introduction and add the comment I stressed to the discussion.	
Add another limitation. The fact that you calculated the doses assuming only adults uses the antibiotics	Thank you for this. We have added this limitation and appropriate reference has been cited.
Grammatical and typo errors	Thank you and have been corrected
**REVIEWER 2**	
**General comments**	
The manuscript needs to be reformatted by referring the journal style, excessive bolding of words and excessive unnecessary abbreviations should be reduced, and appropriate citations, repeated editing and proof reading are required. In addition to making the paper mind-numbing, those things are very technical and reduce the readability of the paper, and hence, it needs careful revisions.	Thank you very much for the comment<br><br>We have carefully gone through the manuscript again and reformatted addressing the issues pointed out<br><br>We have revised the entiremanuscript and added the relevant citations
The background, objective, method, results and conclusions of manuscript need to be reformatted including make them bold.	Thank you very much<br>We have reformatted the subheadings as per the Journal style
**Detail comments/suggestions**	
**Title**	
I suggest the title need to be revised. I think it is a cross sectional study, which is a survey, but the title saying surveillance. Unlike your study, surveillance is an ongoing collection of information to detect changes or it is repeated survey.	Thank you very much for the comment<br><br>We have amended the title as<br><br>*A National survey of antibacterial consumption in Sri Lanka*
**Abstract**	
The abstract lacks background	Thank you very much<br>We have added background in the revised manuscript
The word WHO, in method section need to be written in full. You should first write the full term before you abbreviated. Full term do not need to be in caps and brackets, just abbreviations. Similarly for ABC in the	Thank you very much<br>We have addressed this issue in the abstract as well as in the rest of the manuscript whenever applicable

finding section of abstract.	
The sentence "Reserve and watch category antibacterials accounted for 46, 24 and 54% of the total, public and private sector consumption" not clear and needs revisions	Thank you very much<br>We apologize for the error<br>We have amended in the revised manuscript<br>Amended sentence is:<br>*Watch category antibacterials accounted for 46%, 24% and 54% of the total, public and private sector consumption respectively*
The aim of the study and the conclusion is mismatched. The aim is to describe and quantify, but the conclusion seems comparisons between public and private. The recommendation "Our study has provided the evidence that antibacterial surveillance is possible in resource limited countries and it must be made mandatory" needs revisions. Because, the recommendation is beyond the scope of the study, recommended to other resource limited countries are no appropriate, as the method including sampling procedure used in the study are not allowed to infer to other countries. I also suggest to not use the word mandatory since recommendation are not approached as obligatory	Thank you very much. We agree with the comment<br><br>We have amended the aim of the study as<br><br>*To quantify and describe the national antibacterial consumption in Sri Lanka and to examine any differences in the consumption between public and private sector*<br><br>Conclusion is amended as<br><br>*A disproportionately higher use of broad spectrum and Watch category antibacterials was observed in the private sector which needs further investigation. This national ~~annual~~ consumption survey* highlights the need and provides ~~*has provided*~~ *the opening for establishment of an ABC surveillance in Sri Lanka*
I not saw key words. Please add.	We have given the key words in the online system<br>These were the key words: antibacterials; consumption; ~~surveillance;~~ antibacterial resistance; utilization (if possible, we will edit the surveillance as survey in the system)
**Background**	
Revise the typo error (two point after the title) of "Introduction:"	Thank you very much<br>We have corrected this typo error
The paper generally needs serious citation revisions. For example, in the first paragraph only, from five sites that need citations, only one has citation and the rest four are not. Please, also add more evidences/citations for those cited also.	Thank you.<br>We have revised the paper extensively and added citations as appropriate
The third paragraph of introduction also missed citations and needs revised again.	Thank you, this has been done. As we have redone the entire paper this is now paragraph 4.

The last paragraph of introduction "therefore conducted a national surveillance of antibacterial consumption (ABC)" needs revisions.	Thank you, revised as given below<br><br>*The Sri Lanka Association of Clinical Pharmacology and Therapeutics (SLACPT), in collaboration the National Focal Point for combating AMR in Sri Lanka, therefore conducted this national survey of antibacterial consumption (ABC) for 2017.*
As suggested above to the title, this study is not surveillance rather a survey. The two concepts are quite different.	Thank you very much<br>We have amended the term surveillance to survey
What are the significance of the sentence "This paper outlines the methods adopted, key findings and recommendations for establishment of a national surveillance programme"	This sentence has been modified as below:<br><br>*This paper presents the methods adopted, discusses the key findings and the problems encountered when conducting the survey.*
The background section lacks and needs to revise based on updated prior literatures on global, regional and national picture about the antimicrobial consumption.	Thank you and the background section has been extensively revised with reference to other studies on global, regional and national AMC.
Authors also need to add contribution of this study and practical policy implication.	Thank you, we have added the following:<br><br>*Our findings would be helpful when planning for comprehensive national AMC surveys or surveillances.*
I couldn't see the aim/s and or objective of the study in this section that is mentioned in the abstract, authors need to expand those points including the rationale of the study.	Thank you very much<br>We have included the objective of the study in the last paragraph of the introduction
**Methods**	
There are many bolded disorganized subsections. No need to say background here. Please reformat them based on the journal style. For instance, study design, data source and sampling procedure in to one subsection. Variables of the study (outcome variable vs explanatory variables, including their definitions with appropriate citations, coding and categories in another subsection. Then, data entry, data analysis, and presentation of results as statistical analysis, the ethical issues in another subsection like that.	Thank you very much for the comment<br><br>We have reformatted the methods section with three sub section<br>Materials and Methods<br>1. Study design and data source<br>2. Measures of antibacterial consumption<br>3. Ethical considerations<br><br>Information given in the original manuscript had been reassigned to these three sub sections
Please elaborate a little more on the ethical	Thank you very much for the comment

issues. The ethical issues mentioned in the data source section need to be bring in to ethical consideration section.	We have given some additional information under ethical considerations<br><br>*Ethics Review Committee of Sri Lanka Medical Association exempted this survey from ethics review committee approval (ERC-18/14), Formal request letter with a copy of ethics review committee approval was sent to all the institutions who had the data. Investigators personally visited many of these institutions to explain the aim of survey. Data presented here are from the consenting institutions. Data comprised aggregate data of amount of antibacterials distributed, imported or manufactured by these institutions and not individual patient or hospital data.*
Please summarize data source and availability of data into one.	Thank very much<br>Please see our responses for the first comment in the methods
Authors mentioned as it indicates the 2017 national antimicrobial consumption. However, the data collection period or this specific study is not mentioned.	We have already given this information<br>Second sentence in the methods<br>It was done in 2018
I couldn't found about sampling technique and procedures use in the study. Who were the source and sampled population? How they were selected?	Based on the medicine supply system in Sri Lanka, the data sources we identified are expected to provide the data for the entire country. So, we did not employ any sampling technique<br>We have now given one statement to this effect in lines 25 and 26 under methods<br><br>*These data sources are expected to provide the data for the entire country. Hence, no sampling was done.*
Who were the data collectors? What are the tools?	Data comprised aggregate data<br>They were extracted electronically (Excel worksheet) or manually transferred from the paper format of data submitted by the institutions<br>Hence we have not used data collectors.<br><br>We have now given one statement to this effect in lines 30- 32 under methods<br><br>*Data from the MSD were electronically*

	transferred. All other data sources submitted data in paper format which were manually entered in the Excel worksheet.
**Methods- Additional change done by us**	
In the methods section we have given a formula about calculating defined daily doses. To improve the format of the section, we have converted the formula into text<br><br>*Number of DDDs consumed was calculated by dividing the total consumption (in grams) by DDDs (in grams).*	
**Results**	
The results need to be summarized with subsection to make clearer of the key findings.	Thank you. We have added subsections as appropriate
Please avoid the typo error (colon after the title of results	Thank you and done
**Results – Additional change/s done by us**	
1. We have combined results and discussion under one title as per the journal format<br>2. Additional column in table 2 - DDD per 1000s population<br>3. Additional column in table 4 to show the quality indicators for the country<br>4. Changed figure 2 to tables 5 and 6 to highlight the issues clearly	
**Discussion**	
The discussion is poor. It seems redundancy with results, and therefore, needs careful revisions; compare and contrast of your key findings with prior literatures and justify or discuss in details especially for inconsistency findings. It also have citations problem.	Thank you. The discussion has been extensively revised to compare and contrast our findings with prior literature. The citations have been updated.
**Conclusions**	
The conclusion is not specific and not aligned with the findings. Please first write the word in full before you abbreviated it such as LMICs. You recommend to LMICS, but I couldn't see any evidence in the method sections including sampling technique, that support for generalizability of the findings for LMICs. The conclusion is beyond the study's scope and not specific. Please revise it.	Thank you. We have revised the conclusions to reflect our findings.
**REVIEWER 3**	
This is a comprehensive study looking at antibiotic consumption patterns in a LMIC. It is well-written except for a few grammatical errors (Introduction - line 3, page 5 - line 10, page 20 - lines 1 and 6).	Thank you. The paper has been revised extensively and we have corrected the errors stated.
Data however is far from complete since many potential sources have not been able to	Agree and we have explained this in our limitations.

provide usable data (ie only 12/78 private importers have submitted usable data).	Although there were 78 importers registered with NMRA, the exact number of Companies imported antibacterials in 2017 was not available: Rough estimate from NMRA records was 40. Of them, we have got data from 12. Moreover, of the 5 leading importers, we have got data from 4. In addition, the private sector distribution data from SPC could have captured the data for the Companies who have not submitted data to us<br>We have mentioned in the results section, paragraph 1
A major factor that affects antibiotic consumption in the public hospitals in Sri Lanka is the highly limited formulary that is available for prescribers, compared to the private sector. This fact has not been taken in to account in the Discussion. Being able to 'locally purchase' antibiotics in the public sector does not equate to the choice of antibiotics available in the private sector.	Thank you for this suggestion. We have incorporated it to the discussion as below:<br><br>*A major factor that affects antibiotic consumption in the public hospitals in Sri Lanka is the highly limited formulary available to prescribers. The formulary is based on the WHO's and the country's EMLs and the medicines are made available strictly according to it to the public sector institutions. Even though prescribers have the facility of "local purchase" of medicines, this does not give them the same availability of medicines as those practicing in the private sector as the patients who seek care from these institutions are largely those with limited financial means.*
No information on antibiotic resistance patterns in the country is provided. Therefore, the usefulness and applicability of the prescription patterns is not explored in detail.	Thank you for this suggestion. We have added information on AMR patterns in Sri Lanka both in the Background and results and discussion sections.
**Additional changes done by us**	
a) Surveillance was changed to survey in all places	
