# Supplementary material for: A national survey of antibacterial consumption in Sri Lanka
Source: PLoS One. 2021 Sep 14;16(9):e0257424. doi: 10.1371/journal.pone.0257424 (PMC8439449; doi:10.1371/journal.pone.0257424)
Supplement: Attachment — Submitted filename: Response to reviewers.docx [file pone.0257424.s004.docx]

**PONE-D-21-11873
National surveillance of antibacterial consumption in Sri Lanka**

**Amended title: A National survey of antibacterial consumption in Sri Lanka**

**Response to Reviewers**

| **COMMENTS** | **RESPONSE FROM AUTHORS** |
| --- | --- |
| **ACADEMIC EDITOR** |  |
| Please ensure that your manuscript meets PLOS ONE's style requirements, including those for file naming. The PLOS ONE style templates can be found at  <https://journals.plos.org/plosone/s/file?id=wjVg/PLOSOne_formatting_sample_main_body.pdf> and  <https://journals.plos.org/plosone/s/file?id=ba62/PLOSOne_formatting_sample_title_authors_affiliations.pdf> | Thank you very much  We apologize for not following the journal style  We have now revised the whole manuscript based on the journal style guide for title page, main body, tables, figure and references |
| Please provide additional details regarding participant consent. In the ethics statement in the Methods and online submission information, please ensure that you have specified (1) whether consent was informed and (2) what type you obtained (for instance, written or verbal, and if verbal, how it was documented and witnessed). If your study included minors, state whether you obtained consent from parents or guardians. If the need for consent was waived by the ethics committee, please include this information.  If you are reporting a retrospective study of medical records or archived samples, please ensure that you have discussed whether all data were fully anonymized before you accessed them and/or whether the IRB or ethics committee waived the requirement for informed consent. If patients provided informed written consent to have data from their medical records used in research, please include this information. | Thank you  This is a retrospective study of medicine supplies/imports data  Ethics Review Committee of Sri Lanka Medical Association exempted this survey from review (ERC-18/14).  A formal letter of request with a copy of ethics review committee’s letter of exemption was sent to all the institutions who had the data. Investigators personally visited many of these institutions to explain the aim of survey. Data presented here are from the consenting institutions. Data comprised aggregate data of the amount of antibacterials distributed, imported or manufactured by these institutions and not individual patient or hospital data.  We have stated this under ethical considerations in the methods section |
| We note that you have indicated that data from this study are available upon request. PLOS only allows data to be available upon request if there are legal or ethical restrictions on sharing data publicly. For information on unacceptable data access restrictions, please see <http://journals.plos.org/plosone/s/data-availability#loc-unacceptable-data-access-restrictions> | Please see below (cover letter comment) |
| **Cover letter**   a) If there are ethical or legal restrictions on sharing a de-identified data set, please explain them in detail (e.g., data contain potentially identifying or sensitive patient information) and who has imposed them (e.g., an ethics committee). Please also provide contact information for a data access committee, ethics committee, or other institutional body to which data requests may be sent. b) If there are no restrictions, please upload the minimal anonymized data set necessary to replicate your study findings as either Supporting Information files or to a stable, public repository and provide us with the relevant URLs, DOIs, or accession numbers. Please see <http://www.bmj.com/content/340/bmj.c181.long> for guidelines on how to de-identify and prepare clinical data for publication. For a list of acceptable repositories, please see <http://journals.plos.org/plosone/s/data-availability#loc-recommended-repositories>. | The data underlying this study belong to many third parties, Ministry of Health, State Pharmaceutical Corporation, State Pharmaceutical Manufacturing Corporation and private Pharmaceutical Companies. Raw data used to analyse antibiotic consumption in this manuscript is either state owned or private company/industry owned. Data were given to us under the agreement that the raw data will not be shared. Hence, we are unable to provide raw data in public domain. We confirm that we did not have any special access to the data that other researchers would not have. They have to approach these institutions with the letter from ethics review committee and administrative clearance |
| **REVIEWER 1** |  |
| The manuscript addresses a timely topic which is very important for all the developing countries. It also stresses the possibility of antibiotic abuse (especially second line antibiotics) within the private sector which can be controlled with appropriate measures as discussed in the article. It is well written except for a few changes as per my comments. Statistically, they have only used percentages which is descriptive enough given the content of the article. Please delete the words that were highlighted in the Introduction and add the comment I stressed to the discussion. | Thank you very much. The typo has been deleted. |
| Add another limitation. The fact that you calculated the doses assuming only adults uses the antibiotics | Thank you for this. We have added this limitation and appropriate reference has been cited. |
| Grammatical and typo errors | Thank you and have been corrected |
|  |  |
| **REVIEWER 2** | |
| **General comments** | |
| The manuscript needs to be reformatted by referring the journal style, excessive bolding of words and excessive unnecessary abbreviations should be reduced, and appropriate citations, repeated editing and proof reading are required. In addition to making the paper mind-numbing, those things are very technical and reduce the readability of the paper, and hence, it needs careful revisions. | Thank you very much for the comment  We have carefully gone through the manuscript again and reformatted addressing the issues pointed out  We have revised the entiremanuscript and added the relevant citations |
| The background, objective, method, results and conclusions of manuscript need to be reformatted including make them bold. | Thank you very much  We have reformatted the subheadings as per the Journal style |
| **Detail comments/suggestions** | |
| **Title** | |
| I suggest the title need to be revised. I think it is a cross sectional study, which is a survey, but the title saying surveillance. Unlike your study, surveillance is an ongoing collection of information to detect changes or it is repeated survey. | Thank you very much for the comment  We have amended the title as  *A National survey of antibacterial consumption in Sri Lanka* |
| **Abstract** | |
| The abstract lacks background | Thank you very much  We have added background in the revised manuscript |
| The word WHO, in method section need to be written in full. You should first write the full term before you abbreviated. Full term do not need to be in caps and brackets, just abbreviations. Similarly for ABC in the finding section of abstract. | Thank you very much  We have addressed this issue in the abstract as well as in the rest of the manuscript whenever applicable |
| The sentence “Reserve and watch category antibacterials accounted for 46, 24 and 54% of the total, public and private sector consumption” not clear and needs revisions | Thank you very much  We apologize for the error  We have amended in the revised manuscript  Amended sentence is:  *Watch category antibacterials accounted for 46%, 24% and 54% of the total, public and private sector consumption respectively* |
| The aim of the study and the conclusion is mismatched. The aim is to describe and quantify, but the conclusion seems comparisons between public and private. The recommendation “Our study has provided the evidence that antibacterial surveillance is possible in resource limited countries and it must be made mandatory” needs revisions. Because, the recommendation is beyond the scope of the study, recommended to other resource limited countries are no appropriate, as the method including sampling procedure used in the study are not allowed to infer to other countries. I also suggest to not use the word mandatory since recommendation are not approached as obligatory | Thank you very much. We agree with the comment  We have amended the aim of the study as  *To quantify and describe the national antibacterial consumption in Sri Lanka and to examine any differences in the consumption between public and private sector*  Conclusion is amended as  *A disproportionately higher use of broad spectrum and Watch category antibacterials was observed in the private sector which needs further investigation. This national ~~annual~~ consumption survey* highlights the need and provides *~~has provided~~ the opening for establishment of an ABC surveillance in Sri Lanka* |
| I not saw key words. Please add. | We have given the key words in the online system  These were the key words: antibacterials; consumption; ~~surveillance;~~ antibacterial resistance; utilization (if possible, we will edit the surveillance as survey in the system) |
| **Background** | |
| Revise the typo error (two point after the title) of “Introduction:” | Thank you very much  We have corrected this typo error |
| The paper generally needs serious citation revisions. For example, in the first paragraph only, from five sites that need citations, only one has citation and the rest four are not. Please, also add more evidences/citations for those cited also. | Thank you. We have revised the paper extensively and added citations as appropriate |
| The third paragraph of introduction also missed citations and needs revised again. | Thank you, this has been done. As we have redone the entire paper this is now paragraph 4. |
| The last paragraph of introduction “therefore conducted a national surveillance of antibacterial consumption (ABC)” needs revisions. | Thank you, revised as given below  *The Sri Lanka Association of Clinical Pharmacology and Therapeutics (SLACPT), in collaboration the National Focal Point for combating AMR in Sri Lanka, therefore conducted this national survey of antibacterial consumption (ABC) for 2017.* |
| As suggested above to the title, this study is not surveillance rather a survey. The two concepts are quite different. | Thank you very much  We have amended the term surveillance to survey |
| What are the significance of the sentence “This paper outlines the methods adopted, key findings and recommendations for establishment of a national surveillance programme” | This sentence has been modified as below:  *This paper presents the methods adopted, discusses the key findings and the problems encountered when conducting the survey.* |
| The background section lacks and needs to revise based on updated prior literatures on global, regional and national picture about the antimicrobial consumption. | Thank you and the background section has been extensively revised with reference to other studies on global, regional and national AMC. |
| Authors also need to add contribution of this study and practical policy implication. | Thank you, we have added the following:  *Our findings would be helpful when planning for comprehensive national AMC surveys or surveillances.* |
| I couldn’t see the aim/s and or objective of the study in this section that is mentioned in the abstract, authors need to expand those points including the rationale of the study. | Thank you very much  We have included the objective of the study in the last paragraph of the introduction |
| **Methods** | |
| There are many bolded disorganized subsections. No need to say background here. Please reformat them based on the journal style. For instance, study design, data source and sampling procedure in to one subsection. Variables of the study (outcome variable vs explanatory variables, including their definitions with appropriate citations, coding and categories in another subsection. Then, data entry, data analysis, and presentation of results as statistical analysis, the ethical issues in another subsection like that. | Thank you very much for the comment  We have reformatted the methods section with three sub section  Materials and Methods   1. Study design and data source 2. Measures of antibacterial consumption 3. Ethical considerations   Information given in the original manuscript had been reassigned to these three sub sections |
| Please elaborate a little more on the ethical issues. The ethical issues mentioned in the data source section need to be bring in to ethical consideration section. | Thank you very much for the comment  We have given some additional information under ethical considerations  *Ethics Review Committee of Sri Lanka Medical Association exempted this survey from ethics review committee approval (ERC-18/14), Formal request letter with a copy of ethics review committee approval was sent to all the institutions who had the data. Investigators personally visited many of these institutions to explain the aim of survey. Data presented here are from the consenting institutions. Data comprised aggregate data of amount of antibacterials distributed, imported or manufactured by these institutions and not individual patient or hospital data.* |
| Please summarize data source and availability of data into one. | Thank very much  Please see our responses for the first comment in the methods |
| Authors mentioned as it indicates the 2017 national antimicrobial consumption. However, the data collection period or this specific study is not mentioned. | We have already given this information  Second sentence in the methods  It was done in 2018 |
| I couldn’t found about sampling technique and procedures use in the study. Who were the source and sampled population? How they were selected? | Based on the medicine supply system in Sri Lanka, the data sources we identified are expected to provide the data for the entire country. So, we did not employ any sampling technique  We have now given one statement to this effect in lines 25 and 26 under methods  *These data sources are expected to provide the data for the entire country. Hence, no sampling was done.* |
| Who were the data collectors? What are the tools? | Data comprised aggregate data  They were extracted electronically (Excel worksheet) or manually transferred from the paper format of data submitted by the institutions  Hence we have not used data collectors.  We have now given one statement to this effect in lines 30- 32 under methods  *Data from the MSD were electronically transferred. All other data sources submitted data in paper format which were manually entered in the Excel worksheet.* |
| **Methods- Additional change done by us** | |
| In the methods section we have given a formula about calculating defined daily doses. To improve the format of the section, we have converted the formula into text  *Number of DDDs consumed was calculated by dividing the total consumption (in grams) by DDDs (in grams).* | |
| **Results** | |
| The results need to be summarized with subsection to make clearer of the key findings. | Thank you. We have added subsections as appropriate |
| Please avoid the typo error (colon after the title of results | Thank you and done |
| **Results – Additional change/s done by us** | |
| 1. We have combined results and discussion under one title as per the journal format 2. Additional column in table 2 - DDD per 1000s population 3. Additional column in table 4 to show the quality indicators for the country 4. Changed figure 2 to tables 5 and 6 to highlight the issues clearly | |
| **Discussion** | |
| The discussion is poor. It seems redundancy with results, and therefore, needs careful revisions; compare and contrast of your key findings with prior literatures and justify or discuss in details especially for inconsistency findings. It also have citations problem. | Thank you. The discussion has been extensively revised to compare and contrast our findings with prior literature. The citations have been updated. |
| **Conclusions** | |
| The conclusion is not specific and not aligned with the findings. Please first write the word in full before you abbreviated it such as LMICs. You recommend to LMICS, but I couldn’t see any evidence in the method sections including sampling technique, that support for generalizability of the findings for LMICs. The conclusion is beyond the study’s scope and not specific. Please revise it. | Thank you. We have revised the conclusions to reflect our findings. |
| **REVIEWER 3** |  |
| This is a comprehensive study looking at antibiotic consumption patterns in a LMIC. It is well-written except for a few grammatical errors (Introduction - line 3, page 5 - line 10, page 20 - lines 1 and 6). | Thank you. The paper has been revised extensively and we have corrected the errors stated. |
| Data however is far from complete since many potential sources have not been able to provide usable data (ie only 12/78 private importers have submitted usable data). | Agree and we have explained this in our limitations.  Although there were 78 importers registered with NMRA, the exact number of Companies imported antibacterials in 2017 was not available: Rough estimate from NMRA records was 40. Of them, we have got data from 12. Moreover, of the 5 leading importers, we have got data from 4. In addition, the private sector distribution data from SPC could have captured the data for the Companies who have not submitted data to us  We have mentioned in the results section, paragraph 1 |
| A major factor that affects antibiotic consumption in the public hospitals in Sri Lanka is the highly limited formulary that is available for prescribers, compared to the private sector. This fact has not been taken in to account in the Discussion. Being able to 'locally purchase' antibiotics in the public sector does not equate to the choice of antibiotics available in the private sector. | Thank you for this suggestion. We have incorporated it to the discussion as below:  *A major factor that affects antibiotic consumption in the public hospitals in Sri Lanka is the highly limited formulary available to prescribers. The formulary is based on the WHO’s and the country’s EMLs and the medicines are made available strictly according to it to the public sector institutions. Even though prescribers have the facility of “local purchase” of medicines, this does not give them the same availability of medicines as those practicing in the private sector as the patients who seek care from these institutions are largely those with limited financial means.* |
| No information on antibiotic resistance patterns in the country is provided. Therefore, the usefulness and applicability of the prescription patterns is not explored in detail. | Thank you for this suggestion. We have added information on AMR patterns in Sri Lanka both in the Background and results and discussion sections. |
| **Additional changes done by us** |  |
| a) Surveillance was changed to survey in all places |  |
