# Supplementary material for: A national survey of antibacterial consumption in Sri Lanka
Source: PLoS One. 2021 Sep 14;16(9):e0257424. doi: 10.1371/journal.pone.0257424 (PMC8439449; doi:10.1371/journal.pone.0257424)
Supplement: Attachment — Submitted filename: Responses to Reviewers comments.docx [file pone.0257424.s006.docx]

| Journal Requirements: |  |
| --- | --- |
| Please review your reference list to ensure that it is complete and correct. If you have cited papers that have been retracted, please include the rationale for doing so in the manuscript text, or remove these references and replace them with relevant current references. Any changes to the reference list should be mentioned in the rebuttal letter that accompanies your revised manuscript. If you need to cite a retracted article, indicate the article’s retracted status in the References list and also include a citation and full reference for the retraction notice | Thank you  We have reviewed the reference list. It was complete and correct, none of the articles we have cited have been retracted. Unfortunately, WHO has migrated some of their documents and the links had changed after 18^th^ May 2021 which was the time of our initial submission. We have updated the links as of 9^th^ July 2021.  Some of the references from the WHO have compatibility issues with some web browsers and may not be seen with browsers such as Safari. |
| **Reviewer 3** | PONE-D-21-11873  These comments are for the older version PONE-D-21-11873 and have been addressed in our submission PONE-D-21-11873R1 |
| Introduction – highlighted words to be deleted | This has been addressed in the resubmission PONE-D-21-11873R1 |
| Add another limitation: the fact that you only calculated the doses assuming that only adults used the antibiotics. | This has been addressed in the resubmission PONE-D-21-11873R1 (Page 22)  Now, we have added under limitations also (Page 23) |
| **Reviewer 4** |  |
| PAGE 13: it is acknowledged that the public sector provides most inpatient care compared to the private sector, but it is not well explained what is the link/association between inpatient care and high ABC! | Thank you, Similar to many resource limited countries, in Sri Lanka too, use (and overuse) of ABAs is high in inpatient care. This is because majority of infectious diseases which need antibiotics (septicaemia, meningitis, complicated dengue, pneumonia, etc.) are admitted to hospitals, whereas only patients with mild infections (bacterial pharyngitis, bacterial otitis media, uncomplicated pneumonias and UTIs etc.) are treated as outpatient. In addition, there are limitations in doing bacteriological investigations in outpatient settings. Hence patients that need bacteriological investigations are admitted. Therefore, it is natural to expect high volume of ABC in public sector which handles majority of inpatient care in Sri Lanka. However, this was not the case in our results which showed a higher ABC in the private sector.  Therefore, we have modified the statement to reflect our observation more clearly Previous statement  Despite most of the inpatient care being provided by the public sector health care institutions, the ABC in the private sector (Table 1; data sources 1, 4 and 7) accounted for 246.76 million DDDs compared to 97.96 million DDDs distributed to the public sector by the MSD (data source 2).  Edited statement in the current submission (Page 12)  In Sri Lanka, most of the inpatient care is provided by the public sector while the outpatient care is shared between the public and private sectors [15]. Despite bulk of patient care being provided by the public sector, the ABC in the private sector (Table 1; data sources 1, 4 and 7) was 246.76 million DDDs compared to 97.96 million DDDs distributed to the public sector by the MSD (data source 2). |
| Did you ascertain the proportion of outpatient prescription of drugs with that of inpatient? If not, the argument on inpatient needs to be supported by specific references. | As we have used procurement data, we did not ascertain the proportion of outpatient and inpatient use. However, in our experience about healthcare structure in Sri Lanka, major infectious diseases (confirmed and suspected) are managed as inpatients in public sector in Sri Lanka. Despite this fact, public sector consumption was only 97.96 million DDDs. Public sector’s greater contribution to inpatient care is supported by reference [15] |
| The 74.5% of imports to the private sector is in favor of the idea “that the private sector use more drugs for outpatient care than inpatient care”. | Thank you and we agree. The following has been added to explain the situation in Sri Lanka. As there are no published studies in this area we have stated our experiences and used the words ‘could be”  Added paragraph (Page 12-13)  The higher ABC of private sector could be due to the significant proportion of outpatient care that is provided by the private sector which includes the private hospitals and independent family physicians in the community. The antibiotics prescribed by these sectors are obtained from the retail pharmacies in the private hospitals and in the community and will contribute towards the increased ABC of the private sector. |
| What could justify a higher use of macrolides, quinolones and other beta-lactams in the private sector compare to the public? As all these aspects will have policy implications, it is important to answer (or at least try) these points. | Thank you for the observation. While nothing can justify this high use of broad spectrum and Watch antibiotics by the private sector, we have tried to explain this in the discussion (page 21)as given below:  A significant finding from our survey is the disproportionately higher use of broad-spectrum antibiotics in the private sector when compared to the ABC of the public sector. The disparity is difficult to explain, especially as prescribers in both these sectors being largely the same. In addition, the National Antibiotic Guidelines for empirical treatment of infections [31] have been widely disseminated to the prescribers and they are expected to adhere to these irrespective of the place of practice. A major factor that affects antibacterial consumption in the public hospitals in Sri Lanka is the highly limited hospital formulary available to prescribers. The formulary is based on the country’s EMLs and the medicines are made available strictly according to it to the public sector institutions. Even though prescribers have the facility to “local purchase” medicines, this does not give them the same availability of medicines as those practicing in the private sector as the patients who seek care from these institutions are largely from lower income segments of the society. The freedom to prescribe any medicine that is available in the country and improved financial status of the patients who seek treatment from the private sector could contribute to the greater use of broad-spectrum and newer antibiotics in the private sector. |
| What could also explain the disproportionately higher use of broad spectrum antibacterial in the private sector than in the public? | Thank you for the comment, we have added the following in page 19 and the possible reasons are discussed in pages 21 in detail (see above comment)  While both sectors have consumed large amounts of oral and parenteral antibacterials in the Watch category, this is much more in the private sector. Notable is the disparity in the consumption of ceftriaxone, cefuroxime and meropenem. This is a concern as it would contribute significantly towards the spread of antibiotic resistant organisms in the country |
| Page 20 and 21. [REASONS FOR GREATER USE BRAOD SPECTRUM ANTIBACTERIAL IN THE PRIVATE SECTOR: lack of regulatory oversight, greater accessibility to antibacterial and improved financial status of patients.  REASONS FOR GREATER USE BRAOD SPECTRUM ANTIBACTERIAL IN THE PUBLIC SECTOR: inappropriate prescriptions of antibacterial, incorrect physician perception of the need for antibacterial, high patient volume, fear of bacterial super infection and the limited formulary available to prescriber.] It is difficult to understand how each of these factors affect the use in a different manner in the two different studied setting. | Thank you. We have explained the reasons in the discussion (Pages 19-21.) “lack of regulatory oversight” has been removed as it does not give the correct picture.  Paragraph in the current version:  A significant finding from our survey is the disproportionately higher use of broad-spectrum antibiotics in the private sector when compared to the ABC of the public sector. The disparity is difficult to explain, especially as prescribers in both these sectors being largely the same. In addition, the National Antibiotic Guidelines for empirical treatment of infections [31] have been widely disseminated to the prescribers and they are expected to adhere to these irrespective of the place of practice. A major factor that affects antibacterial consumption in the public hospitals in Sri Lanka is the highly limited hospital formulary available to prescribers. The formulary is based on the country’s EMLs and the medicines are made available strictly according to it to the public sector institutions. Even though prescribers have the facility to “local purchase” medicines, this does not give them the same availability of medicines as those practicing in the private sector as the patients who seek care from these institutions are largely from lower income segments of the society.  The freedom to prescribe any medicine that is available in the country and improved financial status of the patients who seek treatment from the private sector could contribute to the greater use of broad-spectrum and newer antibiotics in this setting.  This statement is based on two studies done to assess antibiotic prescription in the outpatient setting of the public sector in Sri Lanka.  We have modified the sentences as given below for clarity: (Page 21-22)  However, inappropriate prescriptions of antibacterials are seen in the outpatient management of respiratory infections in the public sector hospitals too [32,33]. Patients demand for antibiotics as a “quick fix” for infections, incorrect physician perception of the need for antibacterials, fear of bacterial superinfection in viral diseases and the high patient volume in the outpatient settings which limits time for assessment have led to a greater prescription of broad-spectrum antibiotics [ 33]. |
| Finally, is it possible to know if in both private and public hospitals, there are some guidelines on antibiotic usage that are put in place to guide physicians or pharmacists in healthcare facilities? The idea is to know if there are some protocols to follow before or during prescription. | Thank you and yes there are national guidelines for empirical and prophylactic use of antimicrobials [31]. We have added this. |
| **Reviewer #5 -** | PONE-D-21-11873R1 |
| Reviewer #5: This is an appreciable work done on a very important research area that acts as a compus for the AMR surveillance strategies. I have made some queries and comments in the annotated file and would appreciate that these are attended to ensure more clarity and for ease in understanding the data processing and interpretation for the complex area of AMC. The readers must be able to understand the data and study design in the context of the country where study is conducted. It is appreciable that the authors have addressed the comments of previous reviewers diligently | Thank you |
| Page 10 Para 1  Describe if any list was provided to the respondents or they were left to decide by themselves for medicines with antibacterial effect. It is presumed that the data was invited for all antibacterials irrespective of their EML listing status/.Please clarify | Thank you, Yes, data were requested irrespective of their EML/Formulary status. We provided the ATC code {antibacterials for systemic use (J01)} for those who had the data in ATC format (e.g. MSD, SPC). For others, we provided a list containing the names of antibacterials categorized under TC code antibacterials for systemic use (J01)  We have added the following sentence in page 8  Details of all antibacterials were requested irrespective of their EML or Sri Lankan Formulary listing status. We provided the ATC code (J01) for those who had the data in the ATC format and a list containing the names of antibacterials categorized under J01 for those who did not have the data in the ATC format. |
| Page 10 - **Methodology**  methodology states that data i calculated in DDDs using adult dosages. Were consumption data adjusted for syrups excluding them or keeping a percentage. What is the Sri Lankas population proportion for paediatric and geriatric patients | Thank you for the comment  In Sri Lanka, liquid preparations are not used in geriatric population as they are expensive. Since the dose required for elderly is higher, the requirement of liquid preparations could be many times more than that is required for children. Neither the country nor the great majority of patients can afford this. Even for children, liquid preparations are not uniformly available (*Indian J Pediatr 2021. Feb;88(2):178-179.doi: 10.1007/s12098-020-03409-6. Epub 2020 Jun 20.*  *Percentage of children (0-5 years) in Sri Lanka is about 8.5%*  Using the DDDs for adults when there is paediatric consumption also is an inherent limitation of drug utilization surveys. We have noticed this limitation in other published articles as well. Hence, we have not done any adjustment. We have stated this as a limitation (Page 22, 23) |
| Mention, in Limitation the concerns related to paediatric patients | Thank you, we had done this in PONE-D-21-11873R1 under limitations in page 22 and this is what we have said:  “However, the DDD may not reflect the prescribed daily dose (PDD) for individual patients and cannot be used to measure consumption in paediatric wards since the measure is based on adult dosing [36]. It also does not accurately measure antibacterial consumption in cases of renal or hepatic dysfunction, often underestimating the actual antibacterial usage [36].” |
| Please explain how you have identified duplication of data while the same consumption of supply might be mentioned by manufacturer/importer and the distributor. | Figure 1 shows the supply chain of medicines in Sri Lanka and it shows that there is minimal chance of duplication of data. MSD is the sole supplier of medicines to the public sector. Limitation was “non-responders” and “submission of incomplete data that could not be analysed” mainly the private importers. We have mentioned this in the Results (page 20) and limitations (page 22/23) |
| Mention the source (This is given near Aware Classification) | We have modified this as follows in page ….  AWaRe classification 2019 [9] Page 4 |
| Which AWaRe classification if from WHO was used . If it was AWaRe 207 what strategy was adopted for class assignment to medicine shared in access and watch group both | As cited, we have used the 2019 AWaRe classification |
| **Ethical Consideration** Page 10  at any place has it been mentioned that the institutional and personnel identity contributing to the data will be kept anonymous and coded names or ID will be used in data acquisition and processing | Thank you for the comment. We have not mentioned this. This national survey was done as a service requirement and the identity of the participating institutions was essential to identify the gaps. As such the participating institutions were not deidentified and they have consented to submit data with that understanding. This is the reason for us not to publicly share the raw data as requested by the Journal.  The personnel providing the data have done it in their official capacity and have been identified only as the Institution. |
| **Results and discussion** |  |
| Use lower case for companies please | Thank you, we have corrected these typos. |
| Though dosage form data had been collected but still no discussion and results were shared to help interpret the results and form comparison among the two sectors. | Thank you. We had discussed all our findings. Tables 5 & 6 compares the top 10 oral and parenteral ABC in the two sectors. The following has been added (page 19)  While both sectors have consumed large amounts of oral and parenteral antibacterials in the Watch category, this is much more in the private sector. Notable is the disparity in the consumption of ceftriaxone, cefuroxime and meropenem. This is a concern as it would contribute significantly towards the spread of antibiotic resistant organisms in the country. |
| Again the point that if the data is not adjustable to accommodate the paediatric doses (and only used adult DDDs) then why syrup and suspension formulations remained included the consumption data. The proportion could have been easily shared here in data to access the margin of error created, even if pooled data (Oral liquid forms inclusive data) was used for the discussion of the overall data. | Thank you As indicated earlier, liquid preparations are not extensively used due to cost difference This is the proportion of liquid preparations in different data sources (in million DDDs). DDDs for liquid preparations were also calculated using adult DDD \| Total \| Liquid; Distributed by SPC to private sector \| 163.04 \| 19.75; Distributed by MSD to public sector \| 97.96 \| 1.56; Imported by private sector \| 61.18 \| None; Distributed by SPMC to both to public and private sector \| 59.30 \| 3.2; Manufactured by SPMC \| 56.08 \| None; Distributed by SPC to MSD \| 31.43 \| 1.15; Distributed by SPC to its retail pharmacies (Rajya Osusala) \| 24.41 \| 1.13 Only a negligible amount has been contributed by the liquid preparations |
| It is understandable that the data acquisition in this area is a difficult task. It will be helpful that the supply chain process or hierarchy be depicted pictorially and so that its clear to identify the niche of each respondent organization and see if the data has chance of being duplicated | Thank you and we have added Figure 1: the antibiotic supply chain in Sri Lanka for clarity but would like to retain table 1 as it is important for the discussion. |
| At the moment its manufacturer, importer and distributor data under discussion. Countries vary in their distribution and supply chain processes. The Local purchase discussion do not fit into this context as this is presumably a retail and hospital level transaction | Thank you and we agree. We have deleted the section on local purchase. The antibiotics obtained as local purchase are from retail pharmacies and this could have been captured elsewhere. |
| Table 1  A graphic representation of the results as flow diagram would have been more helpful showing various sources and receiving sectors | Thank you and we have added Figure 1: the antibiotic supply chain in Sri Lanka for clarity but would like to retain table 1 as it is important for the discussion. |
| References | New reference 31 has been added and all references have been checked to ensure that they are current and are cited appropriately. |
