# Supplementary material for: A national survey of antibacterial consumption in Sri Lanka
Source: PLoS One. 2021 Sep 14;16(9):e0257424. doi: 10.1371/journal.pone.0257424 (PMC8439449; doi:10.1371/journal.pone.0257424)
Supplement: Attachment — Submitted filename: Response to Reviewers.docx [file pone.0257424.s007.docx]

| **Comment** | **Response** |
| --- | --- |
| Please provide detail limitation of study after discussion | Thank you  During the last revision, we had already included the paragraph given below at the end of the discussion. We hope this is adequate.  No changes have been made now  *“An important limitation of our study was the inability to capture all national data. This was largely due to incomplete and inadequate record keeping by the Customs and private importers. The inability of the SPMC to provide consumption data based on the sector to which it supplied added to the incomplete national consumption data. For meaningful interpretation of data, the total numbers of DDDs derived as consumption estimates should be adjusted for the population to which the data apply. Despite these limitations we have adjusted for the population (DID) to compare with similar studies as there is no separate DDD for children (21). Therefore, we had to use the DDDs for adults in the calculations although both adults and children would have consumed the antibacterials. “* |
| Please indicate generalizability of findings | Thank you  We have added this statement at the end of discussion  **This is the first time an attempt has been made to document the national consumption of antibacterials in Sri Lanka. While there are some limitations and the actual consumption could be an under estimation, we are confident that the pattern of antibacterial use documented in this paper is unlikely to change even if we have all the whole data on antibacterial consumption. The generalizability of our findings would depend on the systems in place to regulate and survey antibacterial consumption. The paper highlights the need for better regulation of antibacterial consumption and the need for robust surveillance systems. The latter could be both labour and resource intense to LMICs like Sri Lanka.** |
| Please provide detail recommendation as separate heading for policy makers | Thank you  We have added the following paragraphs at the end of the body of the manuscript. A new reference is added (38)  **Recommendations for policy makers**  **We strongly recommend the establishment of robust and sustainable surveillance systems to periodically survey and monitor antibacterial consumption. A central body to coordinate the activities of antibacterial consumption is crucial. Surveillance systems should be developed, and adequate funding and resources to collect and analyze data should be made available. All data should be coded at the point of entry using the ATC classification which would help in analysis of data and to compare the consumption trends with other countries.**  **The data on ABC should be linked with that of AMR to identify trends of antibacterial use and changes in antibacterial sensitivity patterns. The ABC should be reviewed annually to identify trends of use and to regularize antibacterial consumption. Based on the surveillance data, national policies and guidelines for antibacterial use should be developed and measures should be in place to ensure that they are adhered to. Linking up with the WHO programme that has been introduced for LMICs [38] is important to compare the country’s activities with others.** |
